# Na_V_1.1 contributes to the cell cycle of human mesenchymal stem cells by regulating AKT and CDK2

**DOI:** 10.1242/jcs.261732

**Published:** 2024-10-10

**Authors:** Mohammed Fouad Zakaria, Hiroki Kato, Soichiro Sonoda, Kenichi Kato, Norihisa Uehara, Yukari Kyumoto-Nakamura, Mohammed Majd Sharifa, Liting Yu, Lisha Dai, Haruyoshi Yamaza, Shunichi Kajioka, Fusanori Nishimura, Takayoshi Yamaza

**Affiliations:** ^1^ Department of Molecular Cell Biology and Oral Anatomy, Kyushu University Graduate School of Dental Science, Fukuoka 812-8582, Japan; ^2^ Department of Periodontology, Faculty of Dental Science, Kyushu University, Fukuoka 812-8582, Japan; ^3^ Department of Nursing, Fukuoka School of Health Sciences, Fukuoka 814-0005, Japan; ^4^ Department of Pediatric Dentistry, Kyushu University Graduate School of Dental Science, Fukuoka 812-8582, Japan; ^5^ Department of Pharmacy in Fukuoka, International University of Health and Welfare, Okawa 831-8501, Japan

**Keywords:** Na_V_1.1, *SCN1A*, CDK2, AKT, Mesenchymal stem cells, Cell cycle

## Abstract

Non-excitable cells express sodium voltage-gated channel alpha subunit 1 gene and protein (known as *SCN1A* and Na_V_1.1, respectively); however, the functions of Na_V_1.1 are unclear. In this study, we investigated the role of *SCN1A* and Na_V_1.1 in human mesenchymal stem cells (MSCs). We found that *SCN1A* was expressed in MSCs, and abundant expression of Na_V_1.1 was observed in the endoplasmic reticulum; however, this expression was not found to be related to Na^+^ currents. *SCN1A*-silencing reduced MSC proliferation and delayed the cell cycle in the S phase. *SCN1A* silencing also suppressed the protein levels of CDK2 and AKT (herein referring to total AKT), despite similar mRNA expression, and inhibited AKT phosphorylation in MSCs. A cycloheximide-chase assay showed that *SCN1A*-silencing induced CDK2 but not AKT protein degradation in MSCs. A proteolysis inhibition assay using epoxomicin, bafilomycin A1 and NH_4_Cl revealed that both the ubiquitin–proteasome system and the autophagy and endo-lysosome system were irrelevant to CDK2 and AKT protein reduction in *SCN1A*-silenced MSCs. The AKT inhibitor LY294002 did not affect the degradation and nuclear localization of CDK2 in MSCs. Likewise, the AKT activator SC79 did not attenuate the *SCN1A*-silencing effects on CDK2 in MSCs. These results suggest that Na_V_1.1 contributes to the cell cycle of MSCs by regulating the post-translational control of AKT and CDK2.

## Teaser

**Summary:** Na_V_1.1, encoded by *SCN1A*, plays an important role in DNA replication during the S phase of the cell cycle of mesenchymal stem cells by regulating AKT and CDK2.

## INTRODUCTION

Voltage-gated Na^+^ (Na_V_) channels are expressed on the plasma membrane of excitable cells, such as neurons, skeletomyocytes and cardiomyocytes (de Lera Ruiz and Kraus, 2015). The nine Na_V_ channels (Na_V_1.1 to Na_V_1.9) are regulated by membrane potential and stimulate Na^+^ influx to generate action potentials in an excitable cell. Na_V_ channels form heteromeric complexes of one large α-subunit (260 kDa) with two small β-subunits (33–36 kDa) (Catterall, 2000). The α-subunits, which are multi-spanning transmembrane proteins, functionally define Na_V_ channel subtypes as they contain the voltage sensors and ion-conducting pore (de Lera Ruiz and Kraus, 2015). α-Subunits exhibit different patterns of cellular expression and function. Among the nine α-subunit-coding genes, variants in sodium voltage-gated channel alpha subunit type 1 (*SCN1A*, which encodes Na_V_1.1) have been reported to be associated with several human neurological disorders, such as autism spectrum disorder, epilepsy and hemiplegic migraine (Meisler et al., 2021).

Recent studies have revealed the unique non-canonical cellular localization and functions of Na_V_ channels in non-excitable cells (Black and Waxman, 2013). Na_V_ channels located on the intracellular membranous organelles contribute to multiple cellular functions, including phagocytosis, motility, release of bioactive molecules, regulation of Na^+^/K^+^-ATPase activity, and metastatic activity in astrocytes, microglia, macrophages, dendritic cells and cancer cells. However, little is known about the molecular mechanism underlying the non-canonical cellular functions of Na_V_ channels in non-excitable cells.

The cell cycle regulates cell proliferation, survival, differentiation and aging. The progression and transition of the cell cycle phases is tightly controlled by the activity of a family of cyclin-dependent kinases (CDKs) and their respective cyclins (Matthews et al., 2022). CDK2 is a member of the CDK family of proteins and plays an important role in controlling transition through the G0-G1 phase and progression through the S phase of the cell cycle (Seo et al., 2006; Fagundes and Teixeira, 2021). CDK2 activation mediated by cyclin E proteins can lead to the phosphorylation of the substrate, the retinoblastoma protein (RB1), which is associated with E2F transcription factors, to suppress transcriptional activity. The phosphorylation of RB1 relieves E2F to allow the transcriptional activity of genes for the promotion of essential cell cycle processes, such as DNA replication and mitosis (Seo et al., 2006; Pennycook et al., 2020). Voltage-gated K^+^ channels (K_V_s) have been reported to participate in cell cycle regulation by cooperating with other proteins in a channel activity-dependent or -independent manner (Urrego et al., 2014). However, the cellular metabolic regulation of the CDK2 protein itself in non-excitable cells via Na_V_ channels remains unclear.

Growth factor receptor tyrosine kinase (RTK)-induced mitogenic signaling molecules, such as extracellular signal-regulated kinase 1 and 2 (referred to collectively here as ERK1/2, also known as MAPK3 and MAPK1, respectively) and AKT (herein referring to total AKT), regulate cell cycle progression via cyclin–CDK complexes (Steelman et al., 2004; Stern et al., 2022). The involvement of ion channels has been reported as one of the mechanisms of cell cycle regulation (Blackiston et al., 2009; Rao et al., 2015). However, very little is known about the involvement of mitogenic signal pathways in regulating the cell cycle of non-excitable cells via Na_V_ channels.

Mesenchymal stem cells (MSCs) are typical non-excitable cells (Sundelacruz et al., 2015) and are defined as self-renewing and multipotent (Dimarino et al., 2013). The divided progenies support tissue maintenance and repair throughout human life. According to recent investigations, MSC-based therapies are attractive and potential options for the clinic, owing to the proliferative features of MSCs, as well as their differentiation capacities. The proliferation of MSCs is tightly regulated by the cell cycle (Pauklin and Vallier, 2013; Muhr and Hagey, 2021). However, little is known about the mechanisms that regulate the MSC cell cycle. A recent study reported that electrical stimulation regulates osteogenic differentiation of MSCs through Na_V_s and K_V_s (Zhang et al., 2016). K_V_s participate in the cell cycle progression and DNA synthesis of proliferative MSCs (Zhang et al., 2014), as well as other non-excitable breast cancer cells (Hou et al., 2021). mTORC1-induced Na_V_1.1 participates in the regulation of senescence via Ca^2+^influx-controlled NFAT–ATF3–p53 signaling in MSC-committed preosteoblasts (Chen et al., 2022). However, the involvement of Nav1.1 in the proliferation, cell cycle progression and DNA synthesis of MSCs remains unclear (Chen et al., 2022). In this study, we examined the role of Na_V_1.1 in regulating the cell cycle of normal human MSCs. Functional knockdown of Na_V_1.1 was found to induce cell cycle arrest in the S phase in human MSCs via the reduction and dysfunction of AKT and CDK2. Our results indicate that Na_V_1.1 plays an important role in tight cell cycle regulation of normal human MSCs via AKT and CDK2.

## RESULTS

### MSCs express *SCN1A*

MSCs were isolated from the dental pulp tissues of human deciduous teeth; these cells met the minimum criteria for MSCs (Dominici et al., 2006). The isolated cells could form plastic adherent colonies containing fibroblast-like cells. The cells displayed a typical immunophenotype that was positive for CD146 (MCAM), CD105 (ENG), CD73 (NT5E), CD90 (THY1) and stage-specific embryonic antigen 4, and negative for CD34, CD45 (PTPRC), CD14, CD19 and human leukocyte antigen DR. The cells also exhibited mesenchymal multipotency, transforming into adipocytes, chondrocytes and osteoblast-like cells (Fig. S1).

**Fig. 1.**
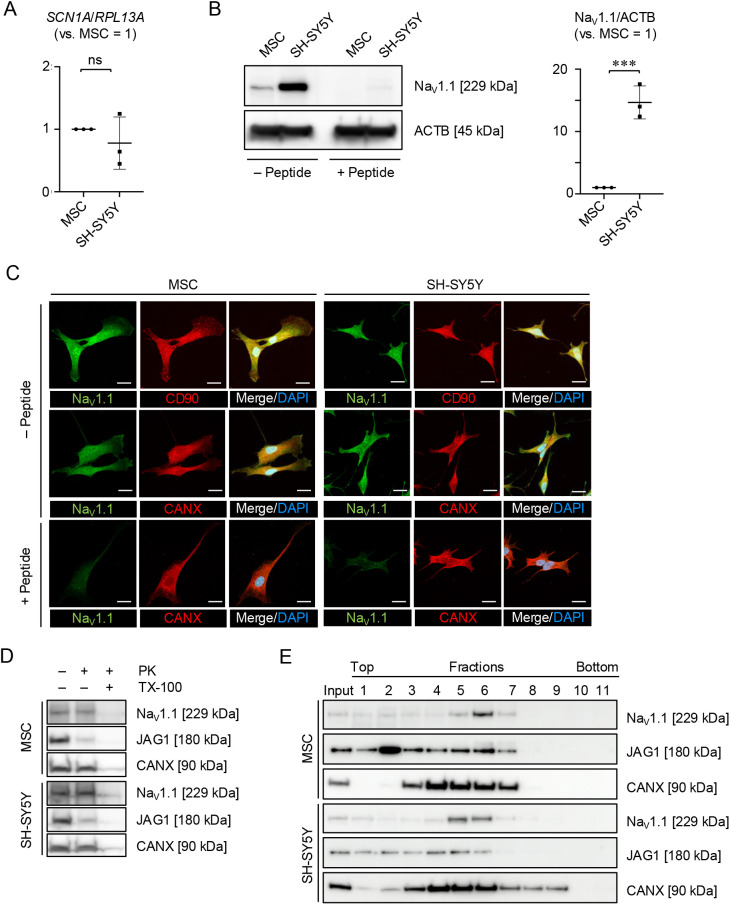
**MSCs express sodium voltage-gated channel alpha subunit 1.** (A) Expression of sodium voltage-gated channel alpha subunit 1 gene (*SCN1A*) in MSCs and SH-SY5Y based on RT-qPCR. The results are presented as the ratios of the expression of *SCN1A* to that of ribosomal protein L13A (*RPL13A*) (versus MSCs=1). (B) Representative WB images of the expression of sodium voltage-gated channel alpha subunit 1 protein (Na_V_1.1) in MSCs and SH-SY5Y. ACTB was used as the internal control. The membrane was incubated with NaV1.1 antibody in the absence or presence of blocking peptide. The graph shows the ratios of Na_V_1.1 expression in MSCs and SH-SY5Y based on WB analysis in the absence of blocking peptide. The results are presented as the ratio of the expression of Na_V_1.1 to that of ACTB (versus MSCs=1). (C) Representative fluorescence images of the localization of Na_V_1.1, CD90 and calnexin (CANX) in MSCs and SH-SY5Y cells based on immunofluorescence. The cells were incubated with Na_V_1.1 antibody in the absence or presence of blocking peptide. The nuclei were stained with 4′,6-diamidino-2-phenylindole dihydrochloride (DAPI). Merge indicates merged images. Scale bars: 10 µm. (D) Representative WB images of the expression of Na_V_1.1, JAG1 and CANX in MSCs based on the proteinase K (PK) protection assay. The cells were treated with or without PK (100 µg/ml) in the presence or absence of Triton X-100 (TX-100, 0.1%) for 30 min. CANX and JAG1 were used for the ER and cell surface protein controls, respectively. (E) Representative WB images of the localization of Na_V_1.1, JAG1 and CANX in MSCs and SH-SY5Y based on density gradient centrifugation. (A–E) SH-SY5Y, human neuroblastoma cell line cells. (A,B) *n*=3/group. Data are mean±s.d. Significance was determined using an independent two-tailed Student's *t*-test; ***P<0.001; ns, no significance. (C–E) Data shown are representative of three independent experiments.

**Fig. 2.**
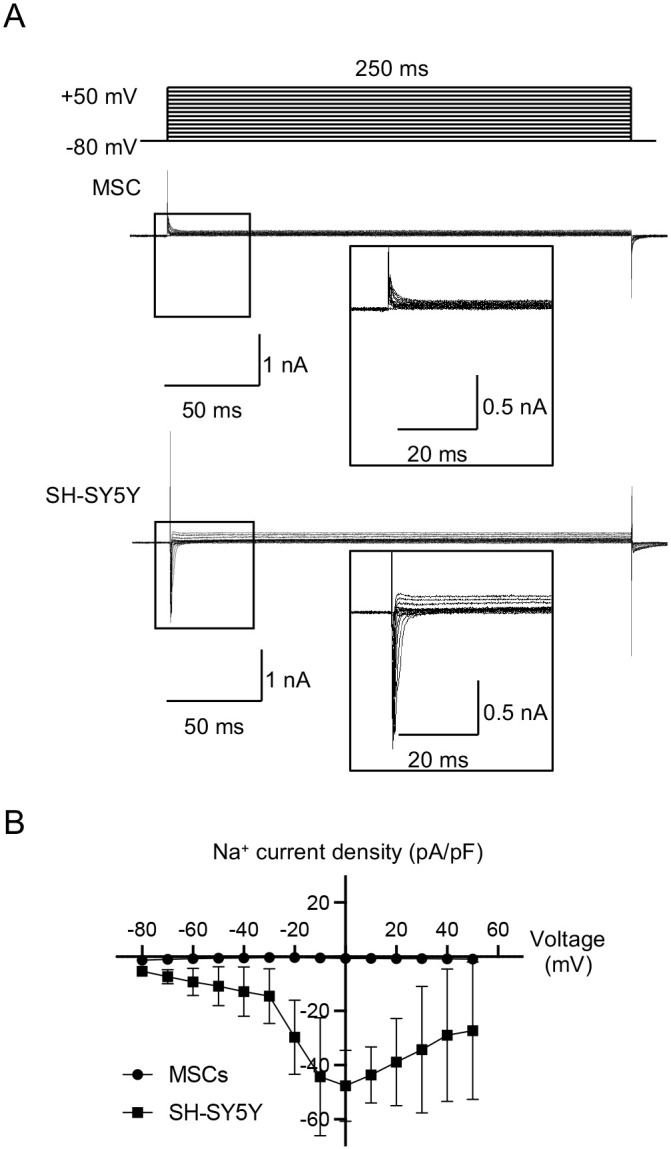
**Voltage-gated Na^+^ current is not detected in MSCs.** (A,B) Voltage-dependent current traces in MSCs and SH-SY5Y cells. Voltage-gated currents were recorded in MSCs and SH-SY5Y cells by blocking an outward K^+^ current using a cesium-based pipet solution. (A) Voltage protocol (top) and representative current traces recorded in MSCs (middle) and SH-SY5Y cells (bottom). The voltage-gated current was traced by 250 ms steps from −80 mV up to +50 mV at 10-mV intervals. The insets show magnified traces of the area outlined on the left (see voltage protocol, truncated at 50 ms). (B) Current density–voltage relation (I–V) in MSCs and SH-SY5Y. *n*=5/group. Data are mean±s.d.

First, we investigated the expression of the *SCN1A* gene and Na_V_1.1 protein in MSCs. SH-SY5Y cells were used as positive controls. SH-SY5Y cells are a human neuroblastoma-derived cell line morphologically characterized as neuroblast-like and non-polarized cell bodies (Biedler et al., 1978). Reverse transcription–quantitative polymerase chain reaction (RT-qPCR) and western blot (WB) analyses revealed that MSCs express *SCN1A* mRNA and Na_V_1.1 protein, as observed in SH-SY5Y cells (Long et al., 2008) (Fig. 1A,B). MSCs exhibited a similar gene expression level to SH-SY5Y cells; however, the protein level in MSCs was lower than that in SH-SY5Y. We examined the localization of Na_V_1.1 using immunofluorescence staining. CD90 is known as a potential cell surface marker of MSCs and neuroblastomas (Ferreira-Facio et al., 2013; Sonoda et al., 2021). Na_V_1.1 was, at least partially, colocalized with CD90 in MSCs and SH-SY5Y cells (Fig. 1C). MSCs and SH-SY5Y cells displayed high colocalization of Na_V_1.1 with calnexin, which is a rough endoplasmic reticulum (ER) protein marker (Fig. 1C). We obtained neutralized antibody to anti-Na_V_1.1 antibody by reacting with the blocking peptide corresponding to the epitope of the antibody and verified the immunoreactive specificity by WB analysis and immunofluorescence (Fig. 1B,C). Further proteinase K protection assay and density gradient centrifugation revealed that MSCs and SH-SY5Y cells displayed a very small amount of surface membranous Na_V_1.1 and a large amount of internalized Na_V_1.1 (Fig. 1D,E). We then analyzed whether MSCs and SH-SY5Y cells expressed functional Na_V_s associated with cell excitability using the whole-cell patch-clamp technique. The currents were recorded by using Cs^+^ containing intracellular solution to minimize K^+^ currents. Transient inward currents were recorded in SH-SY5Y cells (Fig. 2A,B). The transient inward currents were blocked by tetrodotoxin (TTX) in SH-SY5Y cells (Fig. S2). Meanwhile, MSCs did not display such transient inward currents (Fig. 2A,B). Furthermore, the current was not only recorded in MSCs by using two types of classical blockers to K_V_s, including tetraethylammonium chloride (TEA) and 4-aminopyridine (4-AP), but also compensated for using a P/4 leak subtraction protocol (Fig. S3). These electrophysiological findings suggested that MSCs did not display a transient Na^+^ inward current. Therefore, these results indicate that MSC-expressing Na_V_1.1 displayed a unique subcellular localization and exhibited less canonical function for Na^+^ influx into MSCs. This suggests that *SCN1A* is involved in non-canonical function(s) in MSCs under physiological conditions.

### *SCN1A* silencing induces S-phase delay of the cell cycle of MSCs

**Fig. 3.**
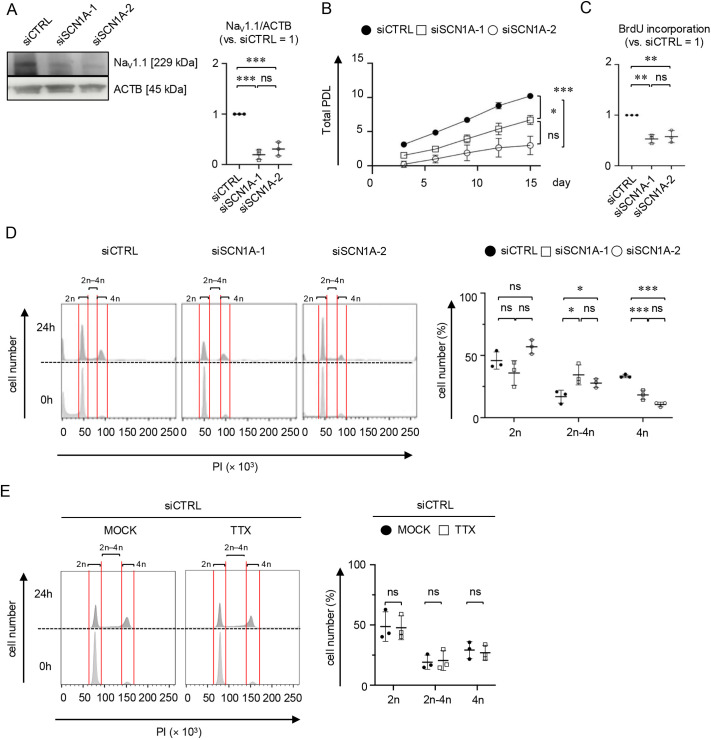
***SCN1A* silencing induces cell cycle arrest at the S-phase in MSCs.** *SCN1A* was silenced in MSCs via transfection with siRNAs against *SCN1A* (siSCN1A; i.e. siSCN1A-1 and siSCN1A-2) and control siRNA (siCTRL). (A) Representative WB images of the expression of Na_V_1.1 in MSCs. The graph shows the ratio of Na_V_1.1 expression in MSCs based on WB analysis. The results are presented as the ratio of Na_V_1.1 to ACTB expression in the indicated MSCs (versus siCTRL=1). (B) Cell proliferation assay. MSCs were sequentially passaged five times every 3 days. The population doubling level (PDL) was calculated at each passage. Graph shows the total PDL at each passage. (C) BrdU incorporation into MSCs. The results are presented as the ratios of the BrdU incorporation rate in the indicated MSCs (versus siCTRL=1). (D,E) FCM of MSCs with PI staining. (D) The indicated MSCs were maintained for 24 h under serum-depleted conditions. MSCs were analyzed at 0 h and 24 h after serum stimulation. (E) siCTRL-MSCs were also analyzed in the presence of 500 nM TTX and H_2_O (MOCK). Representative histograms of the DNA contents (2n, 2n–4n and 4n) in MSCs are shown. The graphs show the percentage of MSCs in each DNA content category based on FCM with PI staining. Data are mean±s.d. *n*=3/group. Significance was determined using two-way ANOVA with Tukey's post-hoc test. *P<0.05; **P<0.01; ***P<0.005; ns, no significance.

Na_V_ channels participate in the cell cycle progress of non-excitable cells, including human colorectal cancer cells and cervical cancer cells (Sui et al., 2021; Sanchez-Sandoval and Gomora, 2019). To determine whether Na_V_1.1 contributes to cell cycle progression in MSCs, we generated *SCN1A*-silenced MSCs by transfecting the cells with two types of small interfering RNA (siRNAs) for *SCN1A*, i.e. siSCN1A-1 and siSCN1A-2, and the non-targeting siRNA siCONT. WB analysis revealed that both siSCN1A-1- and siSCN1A-2-transfected MSCs (siSCN1A-MSCs) showed decreased levels of Na_V_1.1 protein compared with siCONT-transfected MSCs (siCTRL-MSCs) (Fig. 3A). We then investigated cell proliferation using a sequential cell expansion assay. siSCN1A-MSCs exhibited reduced proliferative activity compared with siCTRL-MSCs (Fig. 3B). The bromodeoxyuridine (BrdU) incorporation assay revealed that siSCN1A-MSCs showed decreased BrdU accumulation compared with siCTRL-MSCs (Fig. 3C). Cell cycle analysis using flow cytometry (FCM) and propidium iodide (PI) staining revealed that siSCN1A-MSCs had an increased S-phase (2n–4n DNA content) population and a decreased G2-M-phase (4n DNA content) population, but a similar G0-G1 phase (2n DNA content) population compared with siCTRL-MSCs (Fig. 3D). TTX treatment did not affect the DNA content of siCTRL-MSCs by FCM using PI staining (Fig. 3E). These results suggest that Na_V_1.1 participates in an S-phase progress in MSCs by the reduction of Na^+^ influx-independent *de novo* DNA replication.

### *SCN1A* silencing reduces the expression of CDK2 and AKT in MSCs

**Fig. 4.**
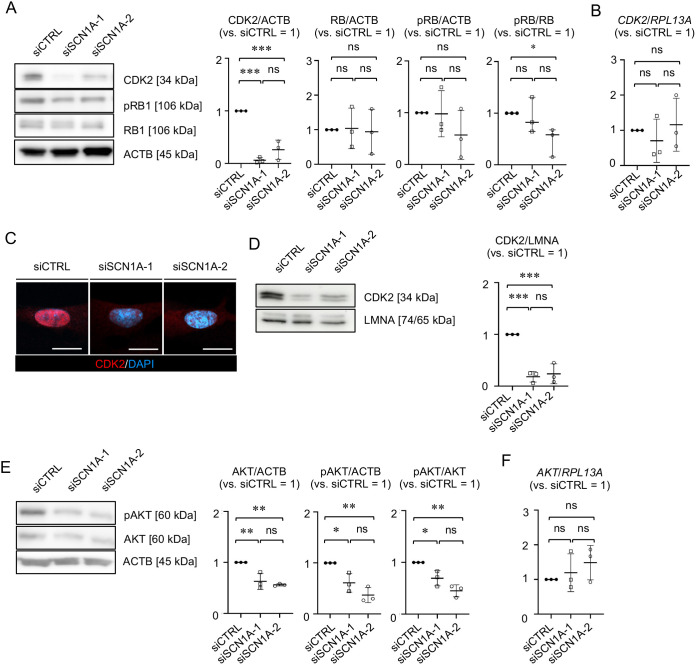
***SCN1A* silencing reduces the expression of CDK2 and AKT in MSCs.** (A) Representative WB images of the expression of CDK2 (RB1 and pRB1), in MSCs. The graphs show the ratios of CDK2, RB1 and pRB1 in the indicated MSCs based on WB analysis. The results are presented as the ratios of the expression of CDK2, RB1 and pRB1 to that of ACTB (versus siCTRL=1). (B) Expression of *CDK2* in the indicated MSCs based on RT-qPCR. The results are shown as the ratios of the expression of *CDK2* to that of *RPL13A* (versus siCTRL=1). (C) Representative immunofluorescence images of the localization of CDK2 in MSCs based on immunofluorescence. The nuclei are stained with DAPI. Scale bars: 10 µm. (D) Representative WB images of the expression of nuclear CDK2 in MSCs. The graph shows the ratios of the expression of CDK2 to that of Lamin A/C (LMNA) in the indicated MSCs (versus siCTRL=1) based on WB analysis. LMNA was used as the internal control of nuclear fraction. (E) Representative WB images of the expression of AKT and pAKT in the indicated MSCs. The graphs show the ratio of the expression of AKT and pAKT to that of ACTB, and of pAKT to AKT (versus siCTRL=1) based on WB analysis. (F) Expression of *AKT* in MSCs based on RT-qPCR. The results are shown as the ratio of *AKT* to *RPL13A* expression in the indicated MSCs (versus siCTRL=1). (A–E) siCTRL, siCTRL-treated group; siSCN1A-1, siSCN1A-1-treated group; siSCN1A-2, siSCN1A-2-treated group. (A,B,D–F) Data are mean±s.d. *n*=3/group. Significance was determined using two-way ANOVA with Tukey's post-hoc test. *P<0.05; **P<0.01; ***P<0.001; ns, no significance. Images in C are representative of three independent experiments.

We investigated the effect of *SCN1A-*silencing on CDK2, RB1 and phosphorylated RB1 (pRB1) in MSCs. WB analysis revealed that siSCN1A-MSCs had similar levels of RB1 and pRB1 compared with siCTRL-MSCs (Fig. 4A). CDK2 expression was suppressed in siSCN1A-MSCs compared with siCTRL-MSCs (Fig. 4A) but was similar between siSCN1A-MSCs and siCTRL-MSCs based on RT-qPCR (Fig. 4B). Immunofluorescence analysis revealed that the nuclear accumulation of CDK2 was lower in siSCN1A-MSCs than in siCTRL-MSCs (Fig. 4C). WB analysis using the nuclear protein fraction (nuclear WB analysis) supported a reduction in nuclear CDK2 in siSCN1A-MSCs compared with siCTRL-MSCs (Fig. 4D). Based on the cell cycle results obtained using FCM, SCN1A regulates the S-phase progression via CDK2 in MSCs, suggesting that SCN1A participates in the self-renewal and proliferation of MSCs via CDK2. We examined the effect of *SCN1A* silencing on mitogenic signaling pathways, including p38 signaling, ERK1/2, catenin β1 (CTNNB1), phosphoinositide 3-kinase (PI3K)–AKT, SRC proto-oncogene non-receptor tyrosine kinase (SRC) and cAMP response element-binding protein (CREB, also known as CREB1). WB analysis revealed that the expression levels of total AKT protein and phosphorylated AKT (pAKT) were lower in siSCN1A-MSCs than in siCTRL-MSCs (Fig. 4E), whereas levels of AKT mRNA were similar between siSCN1A-MSCs and siCTRL-MSCs based on RT-qPCR (Fig. 4F). Meanwhile, the expression levels of other signaling molecules, including p38, ERK1/2, CTNNB1, SRC, CREB, and their phosphorylated forms, did not change between siSCN1A-MSCs and siCTRL-MSCs (Fig. S4). These results indicate that *SCN1A* silencing may cause abnormal post-translational modulation of AKT and CDK2 in MSCs.

### The ubiquitin–proteasome system and the autophagy and endo-lysosome system are not involved in the degradation of AKT and CDK2 in *SCN1A*-silenced MSCs

**Fig. 5.**
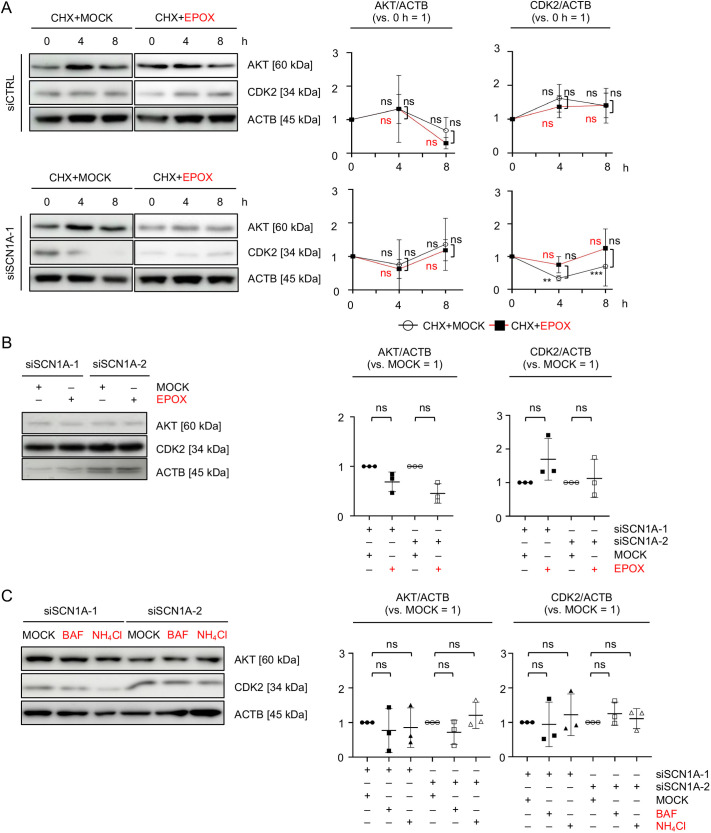
**The reduction of AKT and CDK2 is independent of the ubiquitin–proteasome and autophagy/endo-lysosome systems in *SCN1A*-silenced MSCs.** (A) Representative WB images of the expression of AKT and CDK2 in MSCs at 0, 4 and 8 h during CHX chasing (10 µg/ml) in the presence of MOCK (DMSO) and EPOX (10 µM). The graphs show ratios of the expression of AKT and CDK2 to that of ACTB in MSCs during the CHX chase (versus 0 h=1). CHX+MOCK, CHX and MOCK (DMSO)-treated group; CHX+EPOX, CHX and EPOX treated group. (B) Representative WB images of the expression of AKT and CDK2 in MSCs based on EPOX treatment alone (10 µM) for 8 h. The results represent the ratio of AKT and CDK2 expression in MSCs based on EPOX treatment alone. The graphs show the ratios of the expression of AKT and CDK2 to that of ACTB (versus MOCK=1). MOCK, MOCK (DMSO) treated group; EPOX, EPOX alone treated group. (C) Representative WB images of the expression of AKT and CDK2 in MSCs treated with bafilomycin A1 (BAF; 50 nM) and NH_4_Cl (20 mM) for 6 h. The results represent the ratio of AKT and CDK2 expression in MSCs based on treatment with BAF and NH_4_Cl. BAF, BAF-treated group; NH_4_Cl, NH_4_Cl-treated group. (A–C) siCTRL, siCTRL-treated group; siSCN1A-1, siSCN1A-1-treated group; siSCN1A-2, siSCN1A-2-treated group. (A–C) Data are mean±s.d. *n*=3/group. Significance was determined using an independent two-tailed Student's *t*-test. **P<0.01; ***P<0.001; ns, no significance.

To understand the mechanism of AKT and CDK2 reduction in *SCN1A*-silenced MSCs, we focused on the protein degradation activity in *SCN1A*-silenced MSCs. We performed cycloheximide (CHX) chase analysis with a proteasome inhibitor, epoxomicin (EPOX), referred to as CHX+EPOX chase, and examined the expression of AKT and CDK2 in siSCN1A-MSCs compared with siCTRL-MSCs using WB analysis. As the control, dimethyl sulfoxide (DMSO)-treated MSCs were used, referred to as CHX+MOCK chase. AKT levels did not change in both siCTRL- and siSCN1A-MSCs in a time-dependent manner during the CHX+MOCK and CHX+EPOX chase (Fig. 5A). The levels of CDK2 did not change in siCTRL-MSCs in a time-dependent manner during the CHX+MOCK and CHX+EPOX chase (Fig. 5A). The levels of CDK2 reduced in siSCN1A-MSCs in a time-dependent manner during the CHX+MOCK chase; however, the levels did not change substantially throughout the CHX+EPOX chase (Fig. 5A). By WB analysis, EPOX treatment alone did not affect to the AKT and CDK2 levels in siSCN1A-MSCs compared with MOCK treatments at 8 h after treatment (Fig. 5B). Further WB analysis evaluated that lysosomal enzyme inhibitor treatment using bafilomycin A1 (BAF) and NH_4_Cl had no effect on the AKT and CDK2 levels in siSCN1A-MSCs compared with the MOCK (DMSO) treatment at 6 h after treatment (Fig. 5C). These findings suggest that the ubiquitin–proteasome system and the autophagy and endo-lysosome (autophagy/endo-lysosome) system are not involved in the degradation of AKT and CDK2 expression in *SCN1A*-silenced MSCs.

### The ubiquitin–proteasome system regulates the nuclear translocation of CDK2, but the autophagy/endo-lysosome system does not, in *SCN1A*-silenced MSCs

**Fig. 6.**
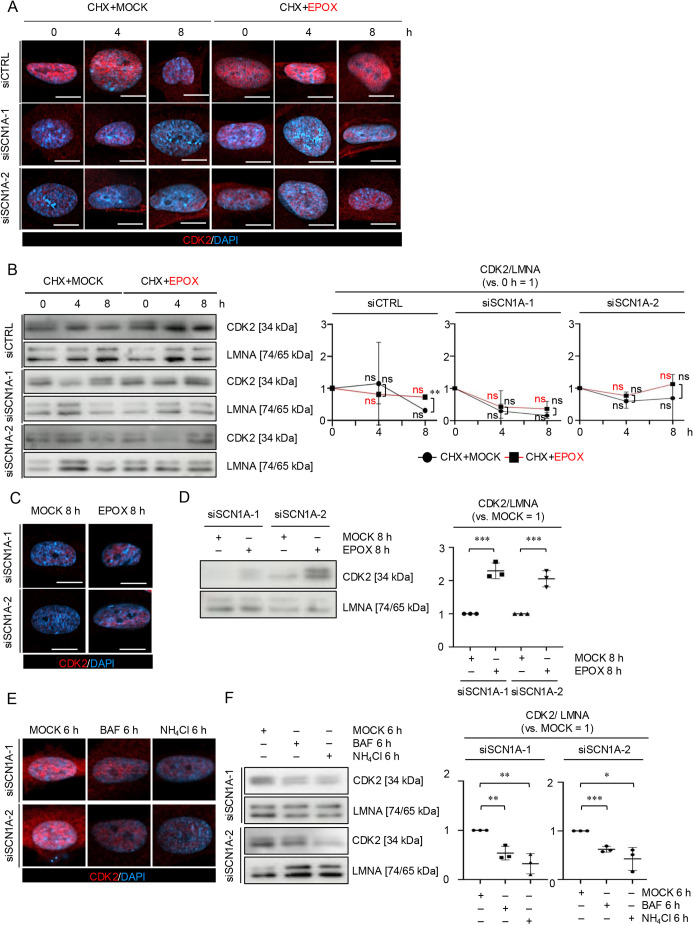
**The nuclear translocation of CDK2 is regulated by the ubiquitin–proteasome systems in *SCN1A*-silenced MSCs.** (A–F) The indicated MSCs were treated with CHX in the presence of either DMSO (MOCK) or EPOX for 0, 4 and 8 h (A,B); with MOCK and EPOX alone (C,D); or with MOCK, BAF and NH_4_Cl (E,F). (A,C,E) Representative immunofluorescence images of the localization of CDK2 in the indicated MSCs based on immunofluorescence analysis. The nuclei were stained with DAPI. Scale bars: 10 µm. (B,D,F) Representative WB images of the expression of nuclear CDK2 in MSCs. The graphs show the ratios of the expression of CDK2 to that of LMNA (versus 0 h=1, B; versus MOCK=1, D,F). (A–F) siCTRL, siCTRL-treated group; siSCN1A-1, siSCN1A-1-treated group; siSCN1A-2, siSCN1A-2-treated group. (A,B) CHX+MOCK, CHX and MOCK (DMSO)-treated group; CHX+EPOX, CHX and EPOX-treated group. (C,D) EPOX, EPOX alone treated group. (E,F) BAF, BAF treated group; NH_4_Cl, NH_4_Cl treated group. (B,D,F) Data are mean±s.d. *n*=3/group. Significance was determined using an independent two-tailed Student's *t*-test. *P<0.05; **P<0.01; ***P<0.001; ns, no significance. Images in A,C,E are representative of three independent experiments.

We then analyzed the nuclear localization of CDK2 in *SCN1A*-silenced MSCs after CHX, EPOX, BAF and NH_4_Cl treatment. Immunofluorescence and nuclear WB analysis revealed that nuclear CDK2 was increased in siCTRL-MSCs 8 h after CHX+EPOX treatment compared with CHX+MOCK treatment (Fig. 6A,B). However, the nuclear CDK2 levels did not change in siSCN1A-MSCs in a time-dependent manner during the CHX+MOCK and CHX+EPOX chase (Fig. 6A,B). Further immunofluorescence and nuclear WB analysis demonstrated that nuclear CDK2 was increased in siSCN1A-MSCs compared with in siSCN1A-MSCs 6 h after EPOX treatment alone (Fig. 6C,D), but was decreased in siSCN1A-MSCs 6 h after BAF or NH_4_Cl treatment (Fig. 6E,F). These findings suggest that the ubiquitin–proteasome system participates in the nuclear translocation of CDK2, but the autophagy/endo-lysosome system does not, in *SCN1A*-silenced MSCs.

### PI3K–AKT activity does not regulate the *SCN1A-*silencing-induced degradation and nuclear translocation of CDK2 in MSCs

**Fig. 7.**
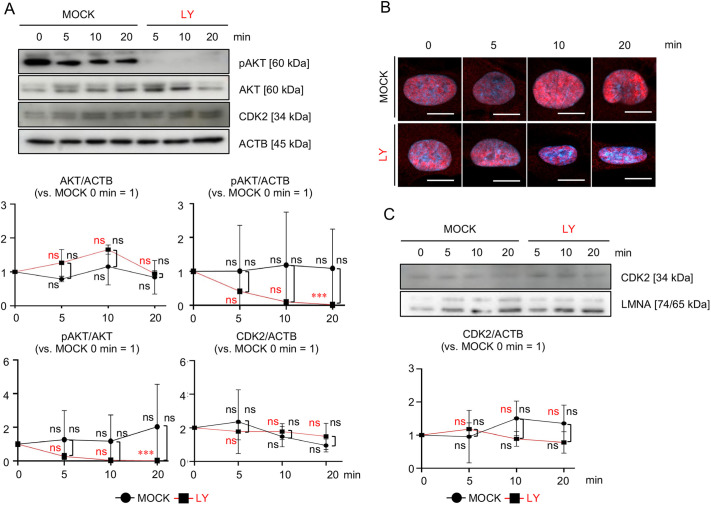
**AKT inhibition does not affect the degradation and nuclear localization of CDK2 in MSCs.** (A–C) MSCs were treated with LY (50 µM) for 0, 5, 10 and 20 min. Representative WB images of the expression of AKT, pAKT and CDK2 in MSCs. Graphs show the ratios of AKT, pAKT and CDK2 expression to ACTB, and the ratio of pAKT to AKT, in MSCs based on WB analysis (versus MOCK 0 min=1). (B) Representative fluorescence images of the nuclear localization of CDK2 (red) in MSCs. The nuclei were stained with DAPI (blue). Scale bars: 10 µm. (C) Representative WB images of the expression of nuclear CDK2 in MSCs. The graph shows the ratios of nuclear CDK2 to that of LMNA (versus MOCK 0 min=1) in MSCs based on WB analysis. MOCK, DMSO-treated group; LY, LY-treated group. (A,C) Data are mean±s.d. *n*=3/group. Significance was determined using an independent two-tailed Student's *t*-test. ***P<0.005; ns, no significance. Images in B are representative of three independent experiments.

**Fig. 8.**
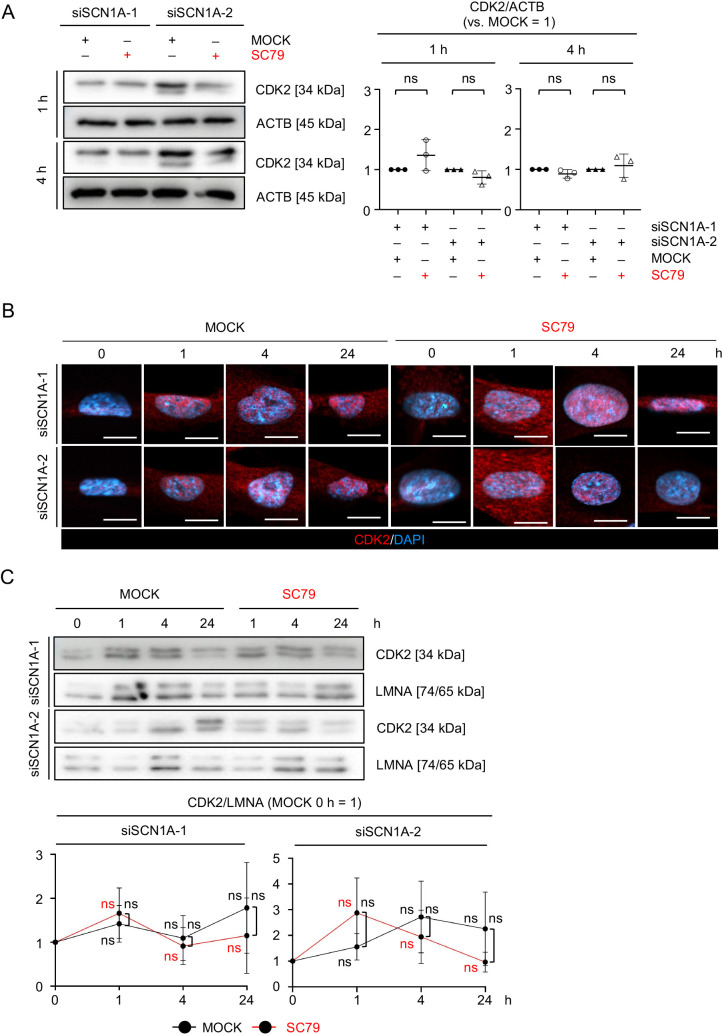
**AKT activation does not affect the reduction and nuclear delocalization of CDK2 in *SCN1A*-silenced MSCs.** (A–C) *SCN1A*-silenced MSCs were pulse-treated with SC79 (10 µM) for 1 h and subsequently maintained under SC79-free conditions for 3 and 23 h (total of 4 and 24 h). (A) Representative WB images of the expression of CDK2 in the indicated MSCs at 1 h and 4 h in the SC79 pulse–chase experiment. The graphs show the ratios of CDK2 expression to that of ACTB (versus MOCK=1) in MSCs at 1 h and 4 h in the SC79 pulse–chase experiment based on WB analysis. (B) Representative fluorescence images of the subcellular localization of CDK2 in MSCs at 0, 1, 4 and 24 h in the SC79 pulse–chase experiment based on immunofluorescence analysis. The nuclei were stained with DAPI. Scale bars: 10 µm. (C) Representative WB images of the expression of nuclear CDK2 in MSCs. The graphs show the ratios of nuclear CDK2 to that of LMNA (versus MOCK 0 h=1) in MSCs based on WB analysis. siSCN1A-1, siSCN1A-1-treated group; siSCN1A-2, siSCN1A-2-treated group; MOCK, DMSO-treated group; SC79, SC79-treated group. (A,C) Data are mean±s.d. *n*=3/group. Significance was determined using an independent two-tailed Student's *t*-test. ns, no significance. Images in B are representative of three independent experiments.

We investigated the effect of AKT phosphorylation on CDK2 kinetics in *SCN1A*-silenced MSCs. MSCs were treated with LY294002 (herein referred to as LY), a PI3K–AKT pathway inhibitor (Zeng and Zhou, 2008), for 5, 10 and 20 min, and its control, MOCK (DMSO), referred to as LY-MSCs and MOCK-MSCs, respectively. WB analysis revealed that AKT phosphorylation was reduced in LY-MSCs compared with MOCK-MSCs after LY treatment, but total AKT and CDK2 did not change during LY treatment (Fig. 7A). Immunofluorescence and nuclear WB analysis revealed that nuclear CDK2 levels were not changed in LY-MSCs compared with MOCK-MSCs (Fig. 7B,C). siSCN1A-MSCs were pulse-treated for 1 h with SC79, an AKT phosphorylation activator (Jo et al., 2012). WB analysis revealed that total CDK2 levels did not change between MOCK (DMSO) and SC79-treated siSCN1A-MSCs at 1 and 4 h (Fig. 8A). Immunofluorescence and nuclear WB analysis revealed that nuclear CDK2 levels also did not change between MOCK and SC79-treated siSCN1A-MSCs at 1, 4 and 24 h. (Fig. 8B,C). These findings suggest that AKT activity is not involved in the degradation and nuclear translocation of CDK2 in MSCs.

## DISCUSSION

The present study evaluated the lower electrophysiological activity and unique subcellular expression of Na_V_1.1 in MSCs, indicating that these channels function in an independent manner. The cell cycle regulates MSC proliferation (Liu et al., 2017, 2019; Deveale et al., 2022). However, the molecular mechanisms underlying the regulation of MSCs remain unclear. Here, Na_V_1.1 was found to be expressed in MSCs. *SCN1A* knockdown affected cell proliferation and the S-phase progression of the cell cycle. *SCN1A* knockdown caused a reduction in CDK2, AKT and pAKT levels, and nuclear delocalization of CDK2 in MSCs. The proteasome inhibitor EPOX restored the nuclear delocalization of CDK2 in siSCN1A-MSCs, but failed to do so during CHX chasing. The PI3K–AKT inhibitor LY did not cause the reduction and nuclear delocalization of CDK2 in MSCs. The AKT activator SC79 did not restore the reduction and nuclear delocalization of CDK2 in siSCN1A-MSCs. Thus, we showed, for the first time, a new function of Na_V_1.1 in the proliferation of MSCs through S-phase progression via the AKT and CDK2.

Self-renewal and subsequent proliferation are critical features of stem cells that ensure regulation of the appropriate number of stem cells for the development, maintenance, and repair of organs and tissues throughout human life (Hagey et al., 2016, 2020; Clevers and Watt, 2018; Post and Clevers, 2019). Incorrect repair and unrepair of DNA damage cause proliferative dysregulation, including senescence, apoptosis and the malignant transformation of stem cells (Al Zouabi and Bardin, 2020). Recent studies revealed that G2-M-phase-specific regulators play a direct role in the self-renewal of stem cells (Gonzales et al., 2015; Hagey et al., 2020). CDK2 complexed with CCNA2 targets S-phase substrates, such as cell division cycle 6, chromatin licensing DNA replication factor 1 (CDT1) and mini-chromosome maintenance helicases in the nucleus, and subsequently regulates DNA replication (Wuarin and Nurse, 1996; Petersen et al., 1999; Paolinelli et al., 2009). Recently, activated CDK2 was reported to repair DNA damage (Lee and Desiderio, 1999; Müller-Tidow et al., 2004). As *SCN1A* knockdown in MSCs caused a S-phase delay associated with CDK2 protein reduction, these findings suggest that Na_V_1.1 participates in the self-renewal and proliferation of MSCs via CDK2.

In the present study, *SCN1A* knockdown reduced the protein levels of CDK2 and AKT but not their mRNA levels in MSCs, indicating that Na_V_1.1 controls the post-translational degradation of CDK2 and AKT proteins in MSCs. Although CDK2 is degraded by the ubiquitin–proteasome and autophagy/endo-lysosome systems (Ying et al., 2018; Zhang et al., 2022a,b), the present study indicates no relevance of these two proteolytic systems to CDK2 proteolysis in siSCN1A-MSCs. Another degradation system, the calpain system, can regulate cell cycle regulators, such as cyclin D1 and TP53 (Mellgren et al., 1996; Choi et al., 1997; Zhang et al., 1997), suggesting that Na_V_1.1 suppresses CDK2 degradation by proteases other than the proteasomes and lysosomal proteinases in MSCs. However, the Nav1.1-mediated post-translational control of AKT remains unclear.

The present findings in siSCN1A-MSCs indicated that Na_V_1.1 controls CDK2 translocation to the nucleus in MSCs by immunofluorescence and nuclear WB analysis. Nuclear and cytosolic oscillations of CDK2 consistently occur in proliferating cells in a cell cycle-dependent manner; cytosolic CDK2 enters the nucleus in the G1 phase and replicates DNA correctly in the S phase (Blanchard et al., 2000; Dietrich et al., 1997). This kinase subsequently exits the nucleus during late S phase to permit mitosis (Maddika et al., 2008). AKT regulates the G1-S and G2-M transition of the cell cycle by activating various cell cycle regulators, including cyclin D1, cyclin-dependent kinase inhibitor 1A (CDKN1A), CDKN1B, glycogen synthase kinase 3β, FOXO3 and MYC (Medema et al., 2000; Appleman et al., 2002; Kops et al., 2002; Schmidt et al., 2002). AKT is also involved in the nuclear export of CDK2 during S-G2 transition (Maddika et al., 2008). The present results in LY- or SC79-treated siSCN1A-MSCs indicate that in AKT activity is not involved in Na_V_1.1-mediated maintenance and nuclear translocation of CDK2 in MSCs. Given the present findings in protease inhibitor-treated siSCN1A-MSCs, Na_V_1.1 may control the nuclear translocation of CDK2 in MSCs via a factor degraded in the ubiquitin–proteasome system, not in the autophagy/endo-lysosome system.

Ion channels are expressed on the cell surface of neurons to generate action potentials, and various quality controls tightly regulate their cell surface expression (Mercier et al., 2018). ER quality control prevents the trafficking of transmembrane proteins via ER-retention and retrieval (ER-retention/retrieval) signals of the proteins (Nilsson et al., 1989; Zerangue et al., 1999; Wang et al., 2002; Qiu et al., 2009). The RRR ER-retention/retrieval signal in the cytoplasmic domains of Na_V_1.8 (encoded by *SCN8A*) causes ER retention of this channel, and Na_V_β3 (encoded by *SCN3B*) regulates the surface expression of Na_V_1.8 by masking the ER-retention/retrieval signals (Zhang et al., 2008). The transmembrane segments of Na_V_1.8 also retain Na_V_1.8 in the ER (Li et al., 2010). Protein kinase A stimulates the trafficking of Na_V_1.5 (encoded by *SCN5A*) from ER to plasma membrane in rat cardiomyocytes (Hallaq et al., 2006). ER-retention/retrieval signals have not been determined in Na_V_1.1, although Na_V_β1 (encoded by *SCN1B*) interacts with the C-terminal cytoplasmic domain of Na_V_1.1 (Spampanato et al., 2004). The ER retention mechanism of Na_V_1.1 in MSCs was not evaluated in this study. Therefore, further studies on the ER retention mechanism of Na_V_1.1 in MSCs are necessary to understand the non-canonical function of Na_V_1.1.

Na_V_1.1 is predominantly expressed in the gamma-aminobutyric acidergic (GABAergic) neurons (Ogiwara et al., 2007; Tai et al., 2014). The molecular mechanism underlying Dravet Syndrome (DS) caused by variants in *SCN1A* remains unclear (Claes et al., 2001). A recent transcriptome analysis revealed that DS-specific GABAergic cells derived from human induced pluripotent stem cells exhibit enhanced expression of forkhead box M1 (*FOXM1*) and *E2F1* (Schuster et al., 2019). These transcription factors control pathways involved in cell cycle progression (Fischer et al., 2016). We showed that *SCN1A* knockdown suppresses proliferation and abnormal S-phase progression associated with the degradation and nuclear delocalization of CDK2 in MSCs. MSCs exhibit the potential to transform into GABAergic neurons (Cho et al., 2016; Yang et al., 2022). These findings suggest that the aberrant cell cycle caused by *SCN1A* variants may contribute to the development of DS.

Taken together, our findings indicate that Na_V_1.1 plays an important role in DNA replication during the S phase of the cell cycle of MSCs via AKT and CDK2. Further analysis of the effects of pathological *SCN1A* variants on the post-translational control of the AKT and CDK2 in MSCs will enable a better understanding of the molecular mechanism and the development of a novel therapeutic option for DS.

## MATERIALS AND METHODS

### Ethics statement and human participants

Human deciduous teeth were collected from discarded clinical samples of a 7-year-old healthy donor after tooth extraction in the Pediatric Dentistry and Special Needs Dentistry at Kyushu University Hospital (Fukuoka, Japan). Handling of human samples was approved by the Kyushu University Institutional Review Board for Human Genome/Gene Research (protocol number 678-03). Written informed consent was obtained from the guardians of the pediatric donors. All experimental procedures were performed according to the relevant guidelines and regulations, as well as the principles expressed in the Declaration of Helsinki.

### Reagents

Epoxomicin (EPOX) was purchased from Selleckchem, and cycloheximide (CHX), LY294002, proteinase K (PK) and Triton X-100 (TX-100) were purchased from Fujifilm Wako Pure Chemicals. ProLong Glass mounting medium was purchased from Thermo Fisher Scientific and 4′,6-diamidino-2-phenylindole (DAPI) and Hoechst 33258 (Hoechst) were purchased from Dojindo. SC79 and bafilomycin A1 (BAF) were purchased from Cayman Chemicals. Ammonium chloride (NH_4_Cl) was purchased from Nacalai Tesque. OptiPrep was obtained from Sesrumwerk Bernburg.

### Antibodies

The antibodies used in this study are summarized in Tables S1 and S2. For neutralization of the anti-Na_V_1.1 antibody, blocking peptide corresponding to the epitope of rabbit anti-Na_V_1.1 antibody (Alomone Labs) was purchased from Alomone Labs. The blocking peptide was mixed with the anti-Na_V_1.1 antibody in 5% BSA (Fujifilm Wako Pure Chemicals) in PBS and incubated for 1 h at 25°C. The ratio of blocking peptide and antibody was 1:1 for WB and 1:5 for immunofluorescence as indicated in the manufacturers' instruction. The cocktail was applied for the following experiment.

### Human MSC culture

Human MSCs were isolated from the dental pulp tissues of human deciduous teeth using the colony-forming unit fibroblast (CFU-F) method, as previously described (Iwanaka et al., 2020; Sonoda et al., 2022). The dental pulp tissues were treated with 3 mg/ml collagenase type I (Worthington Biochemicals) and 4 mg/ml dispase II (Fujifilm Wako Pure Chemicals) in phosphate-buffered saline (PBS) containing 2 mM CaCl_2_ for 60 min at 37°C. After the cells were separated using a 70 μm cell strainer, the obtained single-suspended cells were seeded on a T-75 culture flask and cultured in a complete growth medium (CGM) at 37°C in a humidified 5% CO_2_ incubator. CGM consisted of 15% fetal bovine serum (FBS; Merck), 100 µM L-ascorbic acid 2-phosphate (Fujifilm Wako Pure Chemicals), 250 μg/ml amphotericin B (Fujifilm Wako Pure Chemicals), 100 U/ml penicillin (Nacalai Tesque) and 100 μg/ml streptomycin (Nacalai Tesque) in Eagle's minimal essential medium α modification (αMEM; Fujifilm Wako Pure Chemicals). Non-adherent cells were removed 1 day after seeding. Two weeks after seeding, the attached colony-forming cells were observed under a microscope and passaged for expansion in CGM. The medium was replaced twice weekly. Cells passaged less than ten times were used for subsequent analyses.

### SH-SY5Y culture

SH-SY5Y cells were obtained from the European Collection of Authenticated Cell Cultures (Salisbury, UK). The cells were cultured in 10% FBS (Merck), 250 μg/ml amphotericin B (Fujifilm Wako Pure Chemicals), 100 U/ml penicillin (Nacalai Tesque) and 100 μg/ml streptomycin (Nacalai Tesque) in Dulbecco's modified Eagle medium (DMEM)/Ham's F12 (Fujifilm Wako Pure Chemicals) at 37°C in a humidified 5% CO_2_ incubator according to the manufacturer's instructions. The medium was replaced twice weekly.

### CFU-F assay

Isolated MSCs were seeded at 2×10^2^ cells per dish in 10 cm dishes and incubated in CGM. Non-adherent cells were removed 1 day after seeding, and the cells were subsequently maintained in CGM for 14 days. The cells were fixed with 4% paraformaldehyde (Merck) in 0.1 M PBS (pH 7.4) for 10 min at 25°C. After washing with distilled water, the culture samples were incubated in 2% Toluidine Blue solution (Merck) at 25°C overnight. Colony images were captured using an EPSON GTX-970 scanner.

### Immunophenotype of MSCs

The immunophenotype of MSCs was analyzed by FCM. MSCs (2×10^5^) were stained with 1 µg/ml R-phycoerythrin (R-PE)-conjugated antibodies or isotype-matched antibodies (Thermo Fisher Scientific). The stained cells were analyzed using a BD FACSVerse flow cytometer (BD Biosciences). The number of positive cells was determined using the BD FACSuite software (BD Biosciences) relative to the corresponding control cells stained with isotype-matched antibodies or PBS, in which a false-positive rate of <1% was accepted. The antibodies used for FCM are listed in Table S1.

### Mesenchymal multipotency of MSCs

MSCs were seeded at a density of 1×10^5^ cells/well in six-well culture plates and cultured in CGM. After reaching confluence, the cells were cultured under mesenchymal lineage-specific induction conditions to generate adipocytes, chondrocytes and osteoblasts. To induce adipocytes, MSCs were maintained for 6 weeks in CGM supplemented with 500 µM isobutyl-methylxanthine (Merck), 60 µM indomethacin (Merck), 0.5 µM hydrocortisone (Merck) and 10 µM insulin (Merck). The cultures were stained with 0.3% Oil red-O (Merck) to detect lipid droplets. Adipocyte-specific genes, including adiponectin (*ADIPOQ*), CCAAT/enhancer binding protein alpha (*C/EBPA*) and fatty acid binding protein 4 (*FABP4*), were analyzed using RT-qPCR. To induce chondrocytes, the cells were maintained for 6 weeks in 15% FBS (Merck), 100 μM L-ascorbic acid 2-phosphate (Fujifilm Wako Pure Chemicals), 2 mM L-glutamine (Nacalai Tesque), 2 mM sodium pyruvate (Nacalai Tesque), 1% insulin-transferrin-selenium mixture (Thermo Fisher Scientific), 100 nM dexamethasone (Merck), 10 ng/ml transforming growth factor β3 (PeproTech), 250 µg/ml amphotericin B (Fujifilm Wako Pure Chemicals), 100 U/ml penicillin (Thermo Fisher Scientific) and 100 µg/ml streptomycin (Thermo Fisher Scientific) in DMEM (Thermo Fisher Scientific). The cultures were stained with Alcian Blue (Merck) to detect the cartilage matrix. Chondrocyte-specific genes, including aggrecan (*ACAN*), collagen type X alpha1 chain (*COL10A1*), and *S*RY-box transcription factor 9 (*SOX9*), were analyzed via RT-qPCR. To induce osteoblast differentiation, the cells were maintained for 4 weeks in CGM supplemented with 10 nM dexamethasone (Merck), 2 mM β-glycerophosphate (Merck) and 100 µM L-ascorbic acid 2-phosphate (Fujifilm Wako Pure Chemicals). The medium was replaced twice weekly. One week after induction, osteoblast-specific genes, including alkaline phosphatase (*ALP*, also known as *ALPL*), bone gamma-carboxyglutamate protein (*BGLAP*) and RUNX family transcription factor 2 (*RUNX2*), were analyzed via RT-qPCR. Four weeks after induction, the cultures were stained with 1% Alizarin Red-S (Merck) to detect the mineralized matrix.

### Density gradient centrifugation

All steps for density gradient centrifugation were performed at 4°C to obtain the fractions of the plasma membrane and ER from total cell homogenate using OptiPrep, which is a density gradient medium containing 60% (w/v) iodixanol, according to the manufacturer's application sheet S21. MSCs and SH-SY5Y cells were cultured under sub-confluent conditions in CGM. The cells were collected with a cell scraper (SPL Life Science) and centrifuged at 800 ***g*** for 5 min. The cell pellets were suspended in 10 times the volume of homogenization buffer containing 0.25 M sucrose, 1 mM ethylenediaminetetraacetic acid (EDTA) and 10 mM 4-(2-hydroxyethyl)-1-piperazineethanesulfonic acid (HEPES)-NaOH (pH 7.4) and homogenized by passing 20 times through a 27-gauge needle (Terumo). The homogenates were then centrifuged at 2000 ***g*** for 10 min and the supernatants were collected. The supernatants were loaded on each continuous 2–25% (w/v) iodixanol gradient and centrifuged at 200,000 ***g*** for 3 h by using a himac CP80NX (Hitachi) equipped with a rotator P40ST (Hitachi). Eleven equal-volume fractions were collected from the top to the bottom and were precipitated with 10% trichloroacetic acid (Fujifilm Wako Pure Chemicals) at 4°C for 30 min and subsequently centrifuged at 12,000 ***g*** for 10 min at 4°C. The pellet was washed with ice-cold acetone twice and resuspended with NuPAGE LDS sample buffer (Thermo Fisher Scientific) containing 3 M Urea (Nacalai Tesque). The samples were incubated at 70°C for 10 min and analyzed by WB.

### Gene silencing

To silence SCN1A, two types of small interfering RNA (siRNAs) for *SCN1A*, hs.Ri.SCN1A.13.2 and hs.Ri.SCN1A.13.3 (Integrated DNA Technologies), were transfected into MSCs using Lipofectamine RNAiMAX (Thermo Fisher Scientific) and referred to as SCN1A-1-MSCs and SCN1A-2-MSCs, respectively. Negative control DsiRNA (Integrated DNA Technologies)-transfected MSCs (siCONT-MSCs) were used as negative controls.

### PK protection assay

MSCs and SH-SY5Y cells were cultured under sub-confluent conditions in CGM. The cells were treated with 1 mM EDTA in PBS at 25°C for 15 min for detachment from the culture dishes. Thereafter, the cells were incubated for 30 min at 4°C in the absence or presence of 100 μg/ml PK and 0.1% TX-100. The samples were precipitated with 10% trichloroacetic acid (Fujifilm Wako Pure Chemicals) at 4°C for 30 min and subsequently centrifuged at 12,000 ***g*** for 10 min at 4°C. The pellet was washed with ice-cold acetone twice and resuspended with NuPAGE LDS sample buffer (Thermo Fisher Scientific) containing 3 M urea (Nacalai Tesque). The samples were incubated at 70°C for 10 min and analyzed by WB.

### Whole-cell patch-clamp electrophysiological analysis

MSCs and SH-SY5Y cells were cultured on untreated glass coverslips at a density of 1×10^4^ cells per cm^2^. Whole-cell patch-clamp recordings were obtained in voltage-clamp mode using Axopatch 200A or 200B amplifiers (Molecular Devices). The tip resistance was 4.5–6 MΩ. Current–voltage relationships were examined using 250 ms step depolarizations to test potentials ranging from −80 mV to +50 mV, from a holding potential of −80 mV. Data were continuously recorded and analyzed using ClampX 10.7 (Molecular Devices) and ClampFit software (Molecular Devices). The Na^+^ current was normalized to the cell capacitance. P/4 leak subtraction protocol was performed using ClampX 10.7 software. All the experiments were performed at room temperature (25°C).

### Recording buffers

The intracellular solution (pipet solution) was composed of 140 mM CsCl, 10 mM ethylene glycol tetraacetic acid (EGTA), 1.5 mM MgCl_2_, 10 mM glucose and 10 mM HEPES (pH 7.2). The pH was adjusted with Tris. The extracellular solution (bath solution) was composed of 135 mM NaCl, 5 mM KCl, 2 mM CaCl_2_, 1 mM MgCl_2_, 10 mM glucose and 10 mM HEPES (pH 7.4). The pH was adjusted with 1 N NaOH. Tetrodotoxin (TTX; 500 nM; Fujifilm Wako Pure Chemicals) was used when Na^+^ current blocking was required. TEA (10 mM; Fujifilm Wako Pure Chemicals) and 4-AP (5 mM; Fujifilm Wako Pure Chemicals) were used when K^+^ current blocking was performed.

### Gene and protein expression of *SCN1A* and Na_V_1.1 in MSCs

The expression of *SCN1A* in MSCs was analyzed using RT-qPCR. The expression and localization of Na_V_1.1 in MSCs were analyzed using WB and immunofluorescence. SH-SY5Y cells were used as positive controls.

### Cell proliferation assay

MSCs were seeded at 1×10^5^ cells per dish in 100 mm dishes and cultured for 1 day in CGM. 2 days after siRNA transfection, the cultured MSCs were collected and the cells were counted using a TC20 cell counter (Bio-Rad). These three steps were repeated five times. The population doubling level (PDL) at each passage was calculated using the following equation: PDL=log2 (harvested cell number at the end of the growth period)/(seeded cell number at the initial growth period). The total score indicated the PD scores at each passage.

### Bromodeoxyuridine incorporation assay

Cells were incubated with BrdU labeling solution for 24 h at 37°C in a humidified incubator containing 5% CO_2_ and examined using a Cell Proliferation ELISA BrdU (colorimetric) kit (Roche), according to the manufacturer's instructions. The cells were fixed with FixDenat and incubated with anti-BrdU-POD for 90 min at room temperature. The cells were then incubated with a substrate solution for 5 min, and the absorbance was measured at 370 nm using a Multiskan GO plate reader (Thermo Fisher Scientific).

### Cell cycle analysis

When the cells reached 50% confluence, the MSCs were maintained in serum-free αMEM for 24 h. The cells were then cultured in CGM for 24 h and harvested for FCM. TTX (500 nM; Fujifilm Wako Pure Chemicals) was applied to MSCs for 24 h when Na^+^ current blocking was performed. For the cell cycle assay, MSCs (2×10^5^) were fixed with 70% ice-cold ethanol and stained at 37°C for 60 min with 0.5 mg/ml PI (Abcam) diluted in PBS containing 0.5 µg/ml RNase (Abcam) for dye permeability. The stained cells were measured using a BD FACSVerse flow cytometer (BD Biosciences) and analyzed using the BD FACSuite software (BD Biosciences). Because PI dye binds the double-strand DNA, the histograms by FCM display DNA contents, including two independent peak populations and the intermediated population: the left-side peak, intermediated; right-side peak, populations indicate 2n, 2-4n, and 4n of DNA contents in cells.

### Nuclear fractionation

MSCs were cultured under sub-confluent conditions in CGM. The cells were treated with 1 mM EDTA in PBS at 25°C for 15 min for detachment from the culture dishes. Thereafter, the cells were treated with an NE-PER Nuclear and Cytoplasmic Extraction Kit (Thermo Fisher Scientific) according to the manufacturer's instructions. Briefly, the cells were suspended in the cytoplasmic extraction reagent (CER) I and were incubated for 10 min at 4°C. CER II was added to the mixture and it was incubated for 1 min at 4°C. The mixture was centrifuged at 15,000 ***g*** for 5 min at 4°C. The pellets were re-suspended in the nuclear extraction reagent and incubated for 40 min at 4°C. The mixture was centrifuged at 15,000 ***g*** for 10 min at 4°C and nuclear fraction was collected as supernatant.

### CHX chase analysis and EPOX treatment

The MSCs were washed three times with PBS when 50% confluency was achieved. MSCs were treated with 10 µg/ml CHX in the absence or presence of 10 µM EPOX. The cells were harvested at 0, 4 and 8 h after CHX treatment. MSCs were treated with 10 µ M EPOX for 8 h. The cells were analyzed using WB and immunofluorescence analyses.

### BAF, NH_4_Cl and LY294002 treatment

The MSCs were washed three times with PBS when 50% confluency was achieved. Thereafter, the MSCs were treated with 50 nM BAF and 20 mM NH_4_Cl for 6 h. MSCs were stimulated with 50 µM LY294002 for 5, 10 and 20 min. The cells were analyzed using WB and immunofluorescence analyses.

### SC79 pulse–chase analysis

The MSCs were washed three times with PBS when 50% confluency was achieved. MSCs were pulse-treated with 10 µM SC79 for 1 h, washed with PBS, and chased under SC79-free conditions. The MSCs were harvested at 0, 1, 4 and 24 h during the pulse/chase period and analyzed using WB and immunofluorescence analyses.

### Immunofluorescence microscopy

Cells were cultured on glass coverslips and fixed with 4% paraformaldehyde (Merck) in 0.1 M sodium phosphate buffer (pH 7.4) for 10 min at room temperature. The cells were then permeabilized with 0.1% TX-100 for 10 min. After blocking with 2% bovine serum albumin (BSA; Fujifilm Wako Pure Chemicals) in PBS for 20 min at room temperature, some cells were incubated with primary antibodies against Na_V_1.1, calnexin, CDK2 and CD90 overnight at 4°C, and then incubated with Alexa Fluor-conjugated secondary antibody for 60 min at room temperature. The other cells were incubated with neutralized antibody to anti-Na_V_1.1 antibody instead of anti-Na_V_1.1 antibody to indicate the specificity of anti-Na_V_1.1 antibody. The nuclei were stained with 1 µg/ml DAPI and the cells were mounted. Fluorescent images were captured using an Axio Imager 2 up-light microscope (Zeiss) equipped with a 63×/1.4 NA oil differential interference contrast Plan-Apochromat objective (Zeiss), optical sectioning Apotome 2 (Zeiss), and AxioCam 506 mono camera (Zeiss). The *z*-stack images were obtained at each 0.25 µm step and captured using the Zeiss ZEN 2010 acquisition software. The fluorescent images were captured under an optimal exposure time that was standardized to each reference in each experiment. The primary and secondary antibodies used for immunofluorescence microscopy are listed in Table S2.

### RNA extraction and RT-qPCR

Total RNA was extracted from cells using an RNeasy Mini Kit (Qiagen). First-strand cDNA was synthesized using the ReverTra Ace qPCR RT Master Mix (Toyobo). The primer sequences used in this study are listed in Tables S3 and S4. RT-qPCR was performed using FastStart Universal SYBR Green Master Mix (Merck) or FastStart Universal Probe Master Mix (Merck) and a Light Cycler 96 system (Roche). The relative expression of the target genes was analyzed using the comparative threshold cycle method based on normalization to that of ribosomal protein L13A (*RPL13A*) or 18S ribosomal RNA (18S).

### Western blotting

Cells were collected and centrifuged for 5 min at 800 ***g*** and 4°C. The cell pellets were resuspended in PBS and sonicated for 1 min using a BIORUPTOR (COSMO BIO). Total protein was extracted using radioimmunoprecipitation assay (RIPA) buffer supplemented with a protease inhibitor cocktail (Nacalai Tesque) and phosphatase inhibitor, PhoSTOP (Roche). RIPA consisted of 150 mM NaCl (Nacalai Tesque), 1% NP-40 (Nacalai Tesque), 0.5% sodium deoxycholate (Nacalai Tesque) and 0.1% sodium dodecyl sulfate (SDS; Nacalai Tesque) in 50 mM Tris-HCl buffer (pH 7.4) (Nacalai Tesque). The protein samples were incubated in NuPAGE LDS sample buffer (Thermo Fisher Scientific) at 70°C for 10 min. The protein samples (20 µl) were separated on an 8-12.5% SDS-polyacrylamide gel at 100 V for 45-60 min and transferred to an Immobilon-P polyvinylidene fluoride membrane (Merck; 0.45 µm) using a Criterion Blotter (Bio-Rad) at 100 V for 30 min or a Mini Trans-Blot Cell transfer (Bio-Rad) at 300 mA for 60 min. The membranes were blocked in 5% BSA (FUJIFILM Wako Pure Chemicals) or 5% skim milk (Nacalai Tesque) in Tris-buffered saline for 60 min at room temperature and subsequently incubated with the appropriate primary antibody overnight at 4°C. The membranes were incubated with horseradish peroxidase-conjugated secondary antibodies for 60 min at room temperature. Immune reactions were enhanced using ImmunoStar Zeta (Fujifilm Wako Pure Chemicals) and images were captured using an Amersham ImageQuant 800 imaging system (Cytiva). Some membranes were incubated with neutralized antibody to anti-Na_V_1.1 antibody instead of with anti-Na_V_1.1 antibody to indicate the specificity of the anti-Na_V_1.1 antibody. The membranes were re-probed using WB stripping solution (Nacalai Tesque) and reacted with antibodies against actin β (ACTB), which was used as an internal control. The membranes were then incubated with horseradish peroxidase-conjugated secondary antibodies. Finally, band intensity was quantified using ImageJ software (National Institutes of Health). The primary and secondary antibodies used for WB are listed in Table S2. The original WB data are shown as Figs S5-S28.

### Statistical analysis

The results obtained from each group were used as the average of at least triplicate determinations. The results of WB analysis per group were obtained as the ratio of the expression of the target group to that of each control group. All data are expressed as mean±s.d. of the averages per group. Comparisons between two groups were performed using an independent two-tailed Student's *t*-test. Multigroup comparisons were analyzed using one-way repeated measures analysis of variance, followed by Tukey's post-hoc test. *P*<0.05 was considered to indicate statistical significance. All statistical analyses were performed using PRISM 10 software (GraphPad Software).

## Supplementary Material



10.1242/joces.261732_sup1 Supplementary information
