# Supplementary material for: NaV1.1 contributes to the cell cycle of human mesenchymal stem cells by regulating AKT and CDK2
Source: J Cell Sci. 2024 Oct 10;137(19):jcs261732. doi: 10.1242/jcs.261732 (PMC11491812; doi:10.1242/jcs.261732)
Supplement: Supplementary information [file joces-137-261732-s1.pdf]

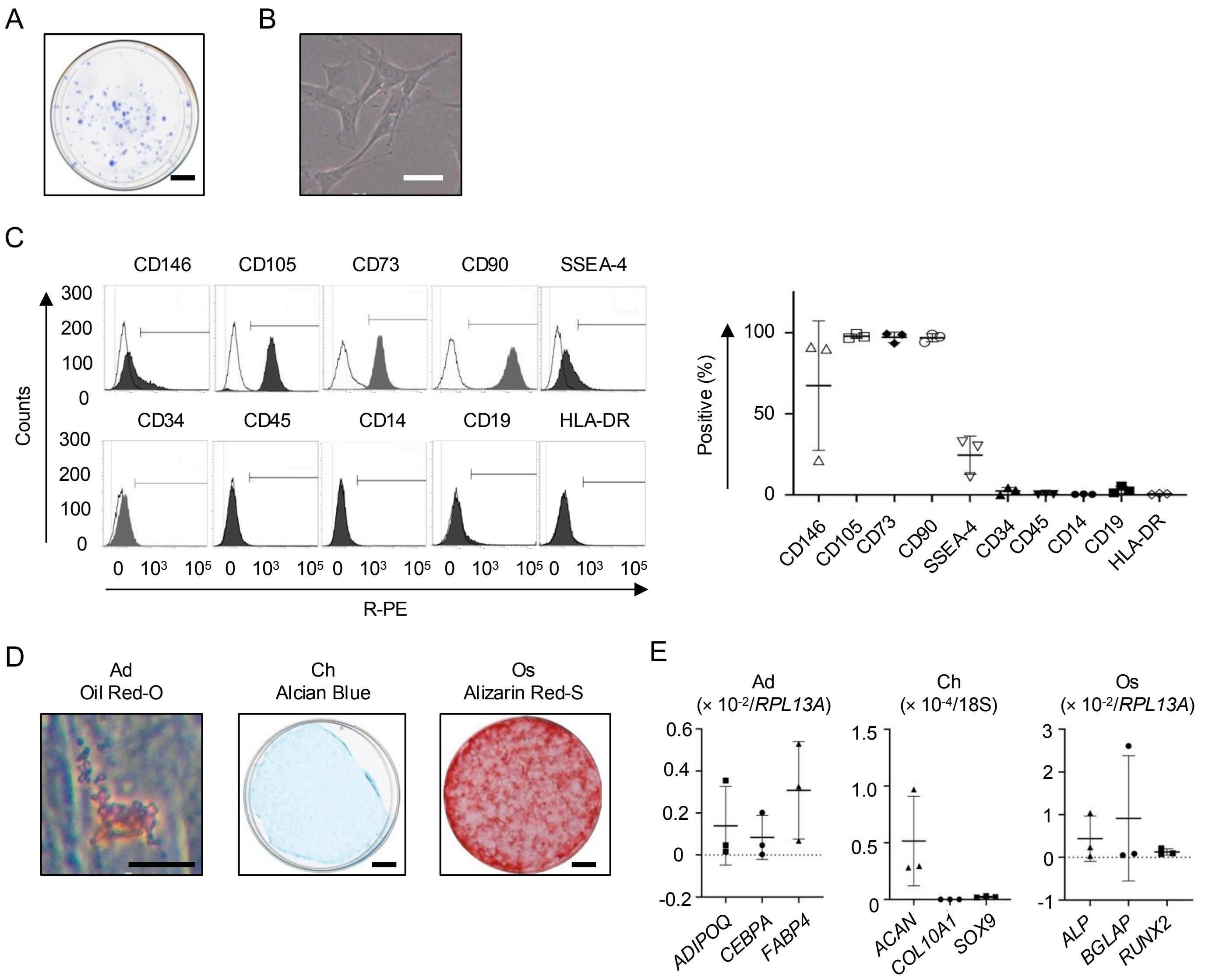

Fig. S1. Stem cells from human exfoliated deciduous teeth exhibit mesenchymal stem cell characteristics.(A) A representative image of attached colony formation of human mesenchymal stem cells (MSCs) based on toluidine blue staining. (B) A representative phase-contrast light micrograph of MSCs. (C) Representative histograms of the expression of cell surface markers on MSCs based on flow cytometry (FCM). White area: histograms stained with the control antibody; black area: histograms stained with antibodies against cell surface markers. R-PE, R-Phycoerythrin. The graph shows positive ratios (%) of the cell surface markers in MSCs based on FCM analysis. (D, E) Mesenchymal multipotency for transformation into adipocytes (Ad), chondrocytes (Ch), and osteoblasts (Os) of MSCs. Representative images of lipid accumulation, cartilaginous matrix formation, and calcified nodule deposition based on oil red, alcian blue, and alizarin red staining (D). Expression of the adipocyte-, chondrocyte-, and osteoblast-specific genes in MSCs based on reverse transcription-quantitative polymerase chain reaction (E). *Ribosomal protein L13A* (*RPL13A*) and 18S ribosomal RNA (18S) were used as internal controls. The results are presented as ratios relative to the expression of *RPL13A* (/*RPL13A*, Ad and Os) and 18S (/18S, Ch) in each group. *ACAN*, *aggrecan*; *ADIPQO*, *adiponectin*; *ALP*, *alkaline phosphatase*; *BGLAP*, *bone-gamma-carboxyglutamate protein*; *CEBPA*, *CCAAT enhancer binding protein alpha*; *COL10A1*, *collagen type X alpha-1 chain*; *FABP4*, *fatty acid binding protein 4*; *RUNX2*, *runt-related transcription factor 2*; *SOX9, SRY-box 9* (E). (A, B, D) Scale bars, 10 mm (A, Ch and Os in D) and 10 µm (B, Ad in D). (C, E) Data are presented as mean ± SD. n = 3/group.

Journal of Cell Science • Supplementary information

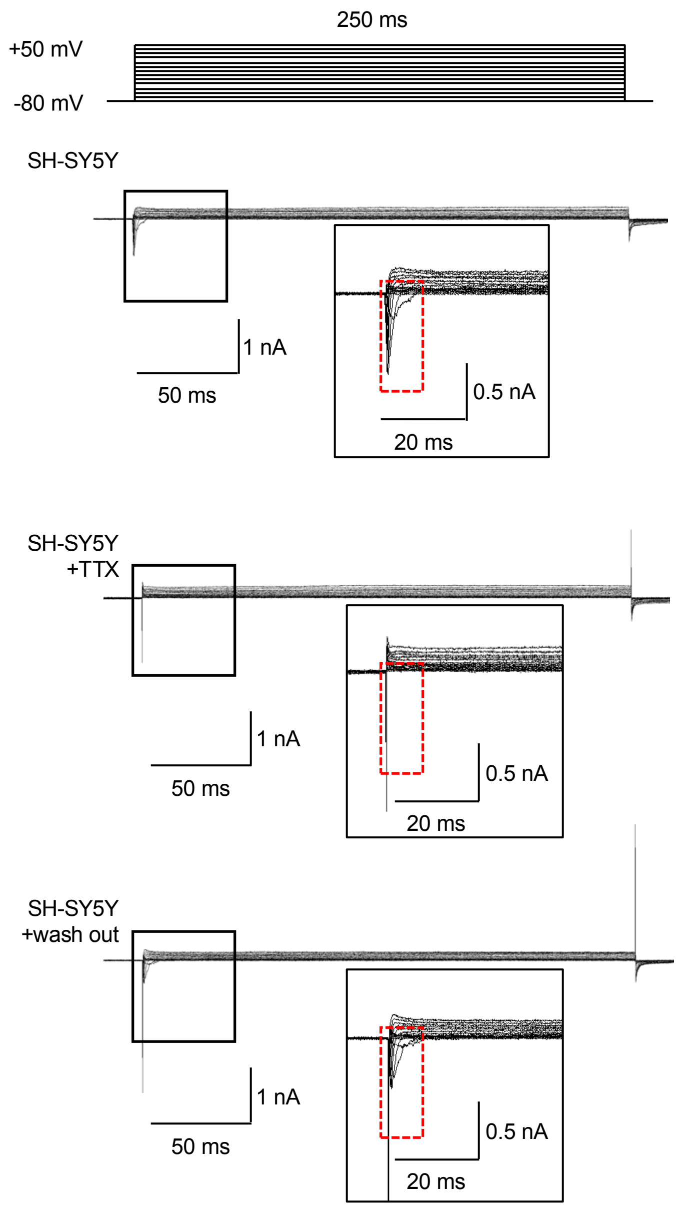

Fig. S2. Voltage-dependent sodium current in SH-SY5Y. Voltage protocol (top) and representative current traces were recorded in SH-SY5Y cells under blocking an outward potassium current using cesium-based pipet solution, under the presence of voltage-gated sodium channel blocker tetrodotoxin (+TTX, 500 nM), and under a condition with TTX removed (+wash out). The voltage-gated current was traced by 250 ms steps from –80 mV up to +50 mV at 10-mV intervals. The inserts were high-magnified traces of the corresponding box (see voltage protocol, truncated at 50 ms).

Journal of Cell Science • Supplementary information

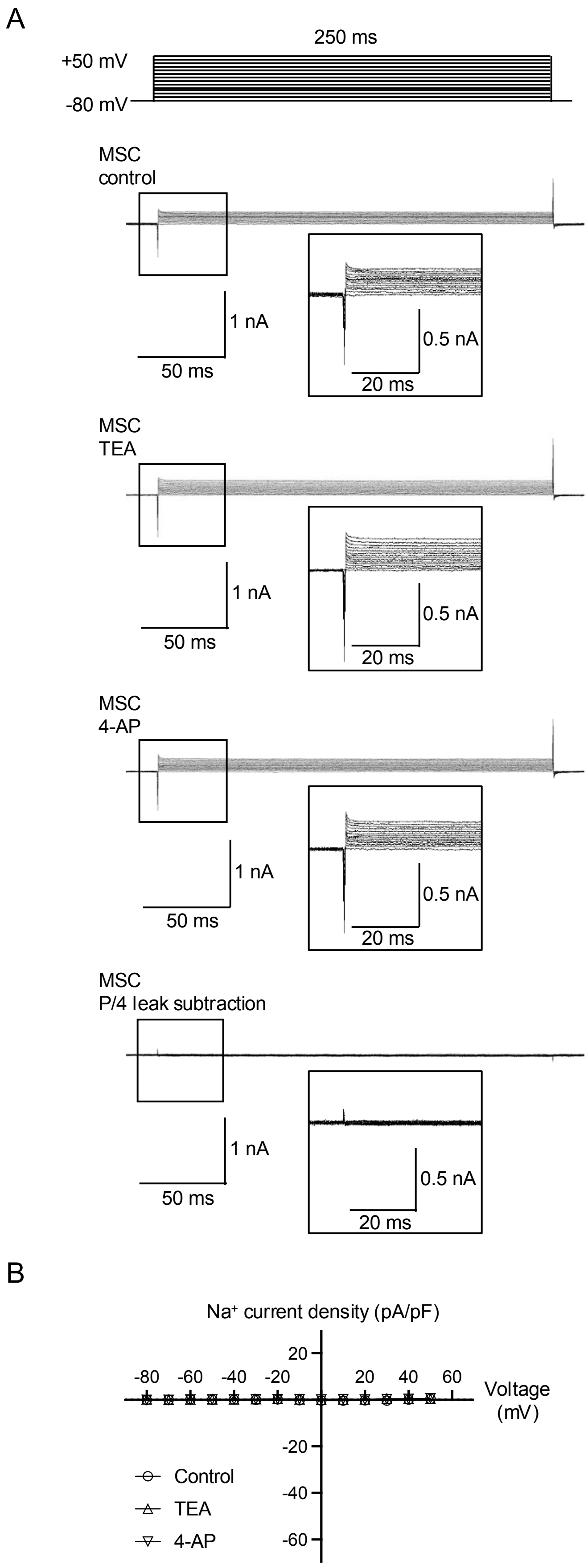

Fig. S3. Effect of K^{+} channel blockers during current recording in MSCs. (A) Voltage protocol (top) and representative current traces were recorded in MSCs under normal conditions (control), in the presence of 10 mM tetraethylammonium (TEA) or 5 mM 4-aminopyridine (4-AP) and under leakage subtracted condition using P/4 protocol (P/4 leak subtraction). The voltage-gated current was traced by 250 ms steps from –80 mV up to +50 mV at 10-mV intervals. The inserts were high-magnified traces of the corresponding box. (B) Current density-voltage relation (I-V) in MSCs. n = 5/group. Data are presented as mean ± SD.

Journal of Cell Science • Supplementary information

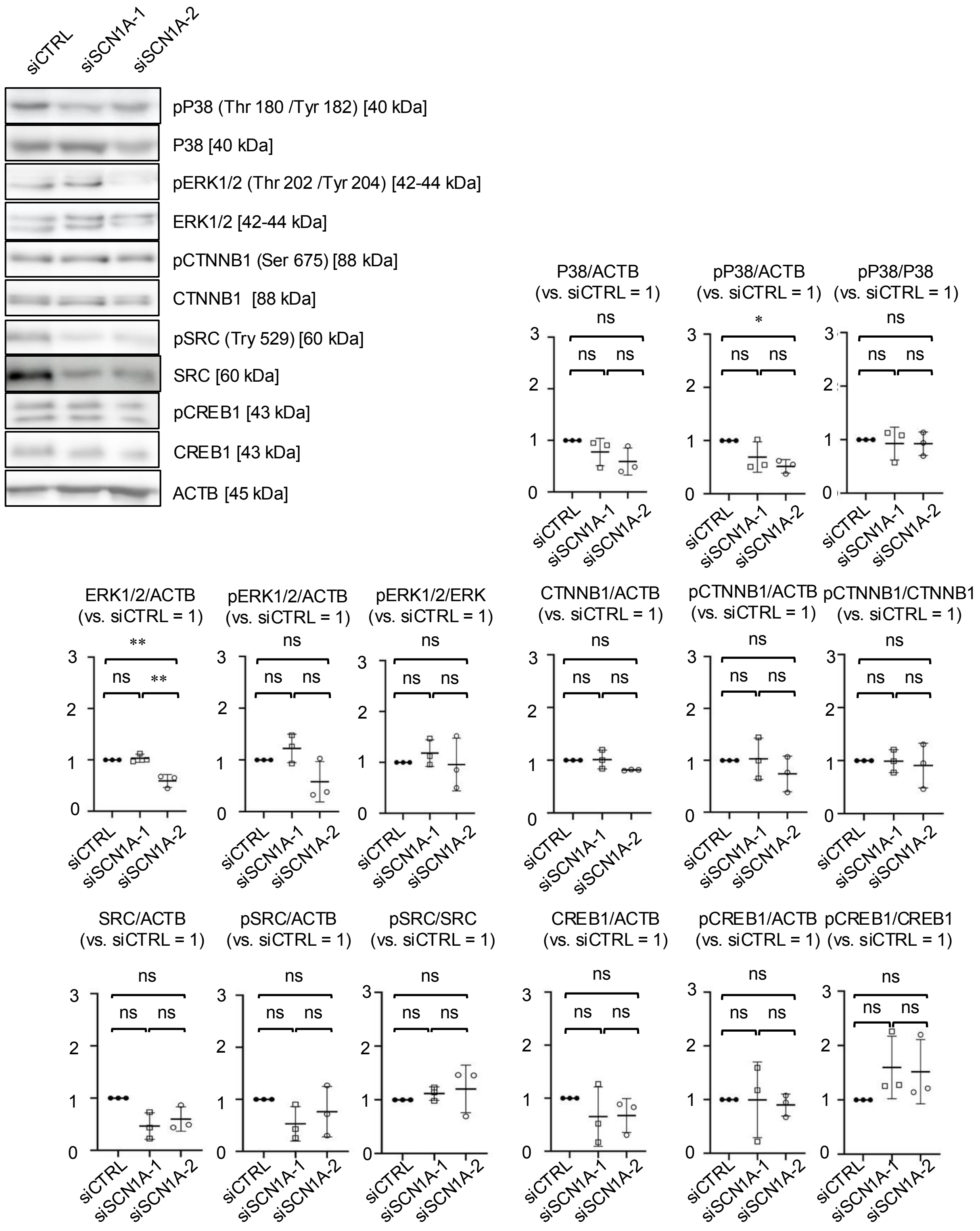

Fig. S4. Expression of intercellular signal molecules in SCN1A silenced MSCs. *SCN1A* is silenced in MSCs via the transfection of small interferent RNA (siRNA) for SCN1A (siSCN1A), siSCN1A-1 and siSCN1A-2, and their control siRNA (siCTRL). Representative Western blot (WB) images of the expression of P38, phosphorylated P38 (pP38), extracellular signal-regulated kinase (ERK), phosphorylated ERK (pERK), catenin beta 1 (CTNNB1), and phosphorylated CTNNB1 (p CTNNB1), SRC, phosphorylated SRC (pSRC), cyclic AMP-responsive element-binding protein 1 (CREB), and phosphorylated CREB (pCREB) in MSCs. The graphs show the ratios of the expression of P38, pP38, ERK, pERK, CTNNB1, pCTNNB, SRC, pSRC, CREB1, and pCREB1 in MSCs based on WB. Actin beta (ACTB) was used as the internal control. The results are shown as the ratios of the expression of P38, pP38, ERK, pERK, CTNNB1, pCTNNB, SRC, pSRC, CREB1, and pCREB1 to the expression of ACTB in siCTRL-treated MSCs (vs. siCTRL = 1). Data are presented as mean ± SD. *n* = 3/group. Significance was determined using two-way ANOVA with Tukey's post hoc test; * $p < 0.05$. ns, no significance. siCTRL, siCTRL-treated group; siSCN1A-1, siSCN1A-1-treated group; siSCN1A-2, siSCN1A-2-treated group.

Journal of Cell Science • Supplementary information

Fig. 1B

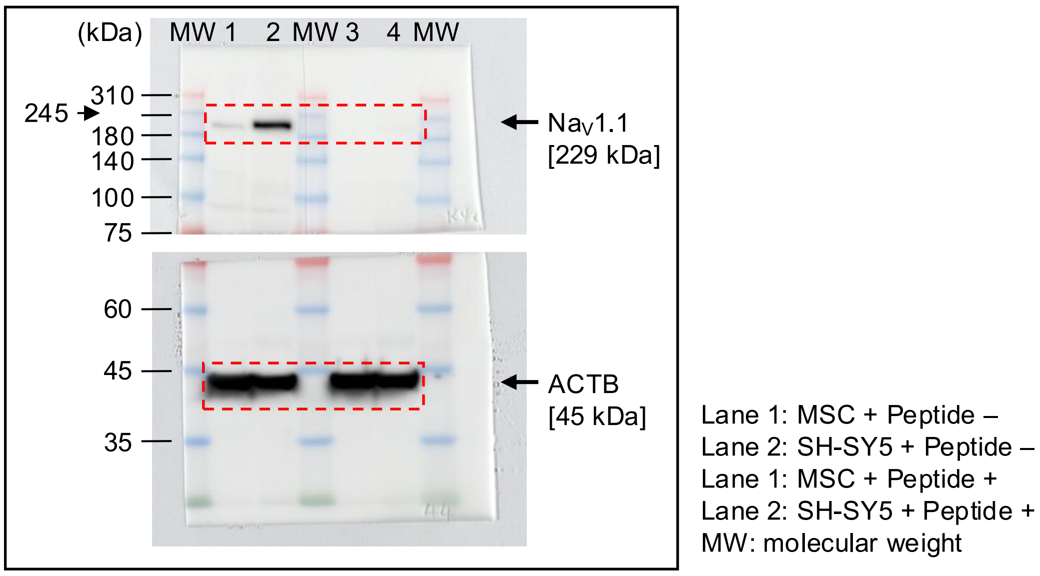

Fig. S5. Full western blot images of Fig. 1B

Journal of Cell Science • Supplementary information

Fig. 1D

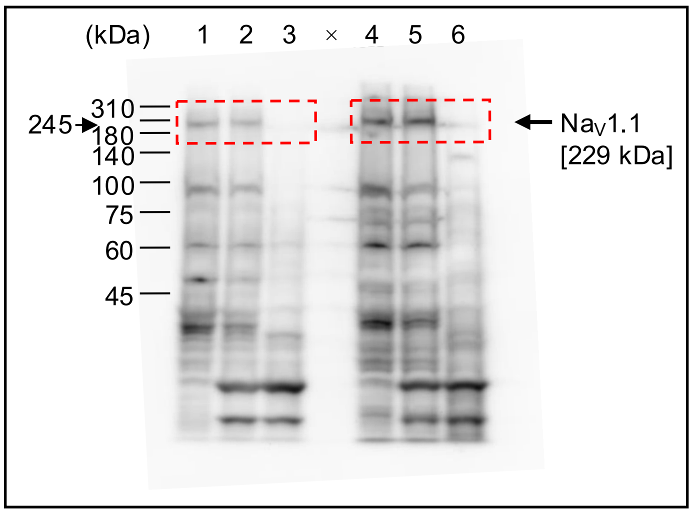

Lane1: MSC PK– TX-100–
Lane2: MSC PK+ TX-100–
Lane3: MSC PK+ TX-100+
Lane4: SH-SY5Y PK– TX-100–
Lane5: SH-SY5Y PK+ TX-100–
Lane6: SH-SY5Y PK+ TX-100+

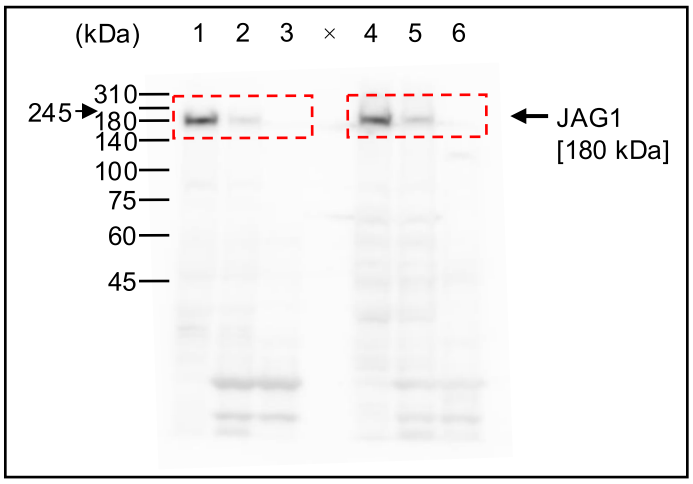

Lane1: MSC PK– TX-100–
Lane2: MSC PK+ TX-100–
Lane3: MSC PK+ TX-100+
Lane4: SH-SY5Y PK– TX-100–
Lane5: SH-SY5Y PK+ TX-100–
Lane6: SH-SY5Y PK+ TX-100+

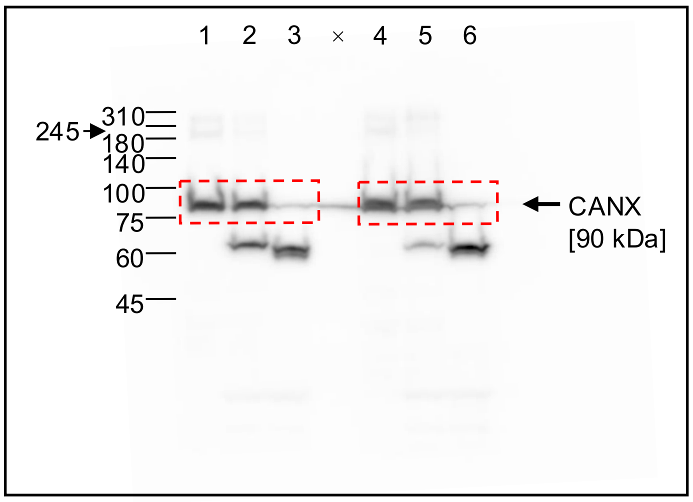

Lane1: MSC PK– TX-100–
Lane2: MSC PK+ TX-100–
Lane3: MSC PK+ TX-100+
Lane4: SH-SY5Y PK– TX-100–
Lane5: SH-SY5Y PK+ TX-100–
Lane6: SH-SY5Y PK+ TX-100+

Fig. S6. Full western blot images of Fig. 1D

Journal of Cell Science • Supplementary information

Fig. 1E

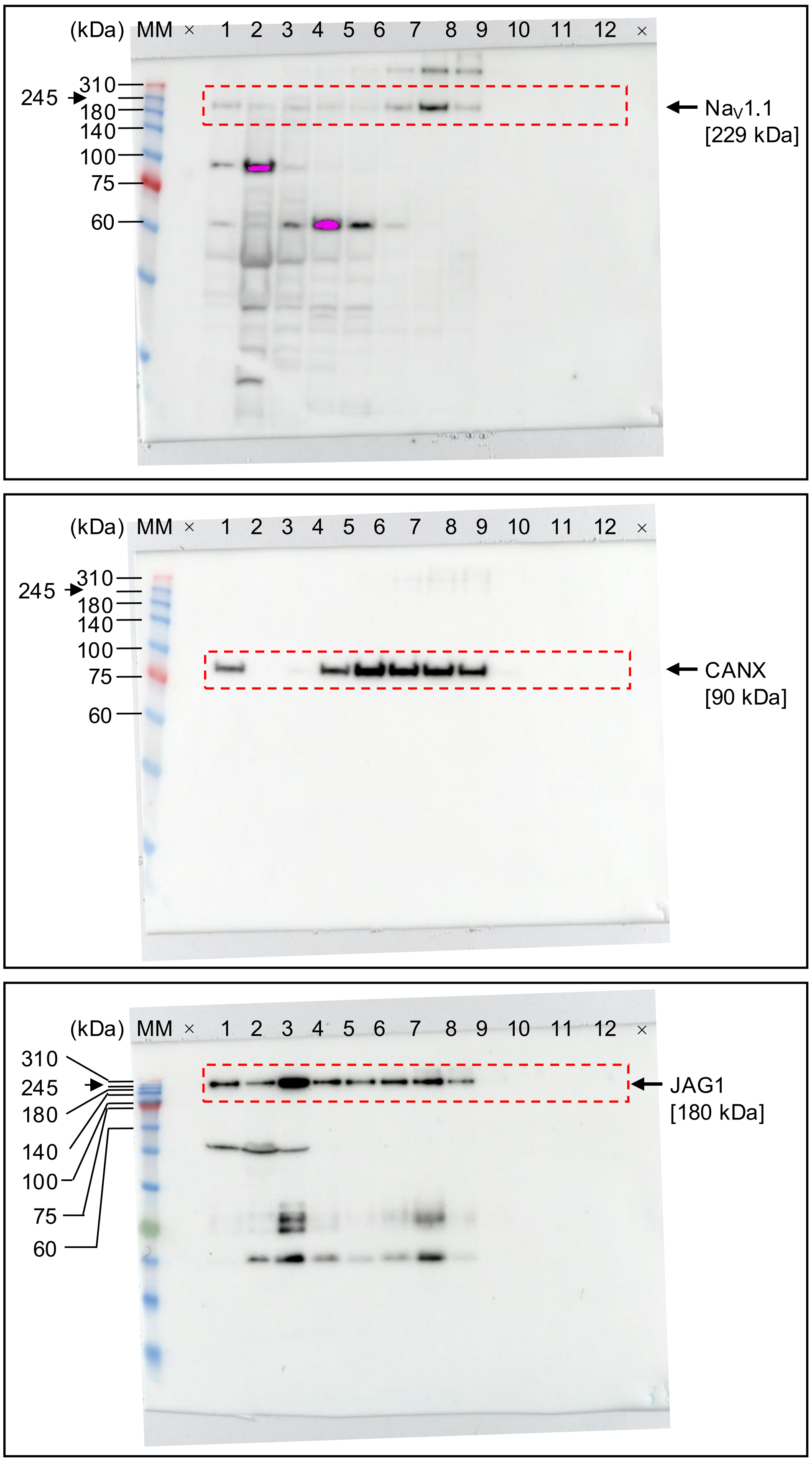

Lane 1: Input
Lane 2: Fraction 1
Lane 3: Fraction 2
Lane 4: Fraction 3
Lane 5: Fraction 4
Lane 6: Fraction 5
Lane 7: Fraction 6
Lane 8: Fraction 7
Lane 9: Fraction 8
Lane 10: Fraction 9
Lane 11: Fraction 10
Lane s12: Fraction 11

Lane 1: Input
Lane 2: Fraction 1
Lane 3: Fraction 2
Lane 4: Fraction 3
Lane 5: Fraction 4
Lane 6: Fraction 5
Lane 7: Fraction 6
Lane 8: Fraction 7
Lane 9: Fraction 8
Lane 10: Fraction 9
Lane 11: Fraction 10
Lane s12: Fraction 11

Lane 1: Input
Lane 2: Fraction 1
Lane 3: Fraction 2
Lane 4: Fraction 3
Lane 5: Fraction 4
Lane 6: Fraction 5
Lane 7: Fraction 6
Lane 8: Fraction 7
Lane 9: Fraction 8
Lane 10: Fraction 9
Lane 11: Fraction 10
Lane s12: Fraction 11

Fig. S7. Full western blot images of Fig. 1E

Journal of Cell Science • Supplementary information

J. Cell Sci.: doi:10.1242/jcs.261732: Supplementary information

Fig. 1E continued

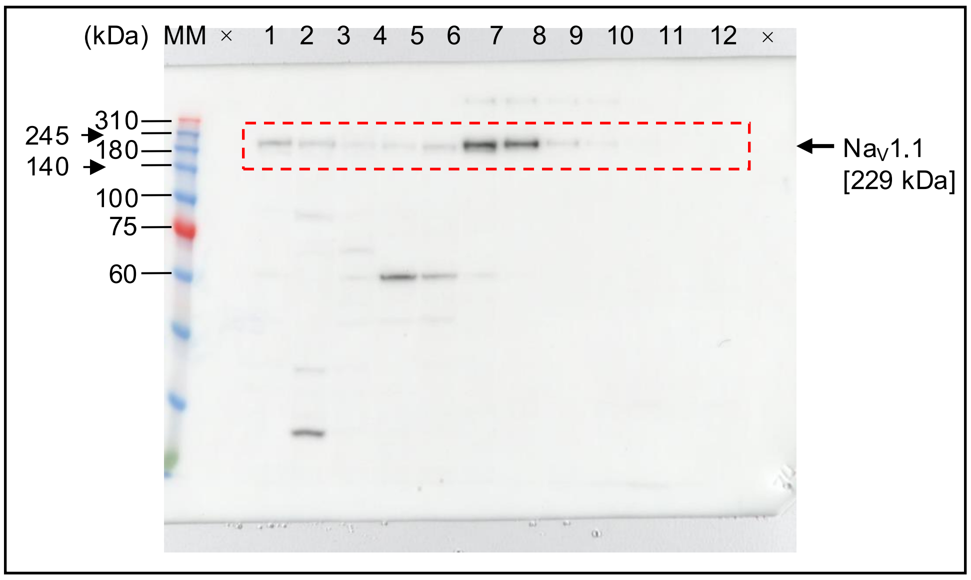

Lane1: Input
Lane2: Fraction 1
Lane3: Fraction 2
Lane4: Fraction 3
Lane5: Fraction 4
Lane6: Fraction 5
Lane7: Fraction 6
Lane8: Fraction 7
Lane9: Fraction 8
Lane10: Fraction 9
Lane11: Fraction 10
Lane12: Fraction 11

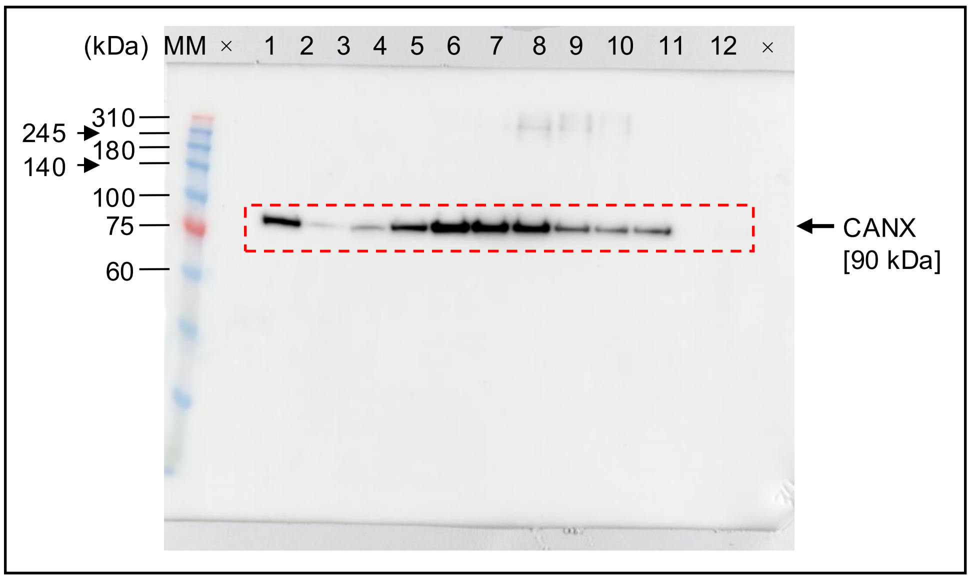

Lane1: Input
Lane2: Fraction 1
Lane3: Fraction 2
Lane4: Fraction 3
Lane5: Fraction 4
Lane6: Fraction 5
Lane7: Fraction 6
Lane8: Fraction 7
Lane9: Fraction 8
Lane10: Fraction 9
Lane11: Fraction 10
Lane12: Fraction 11

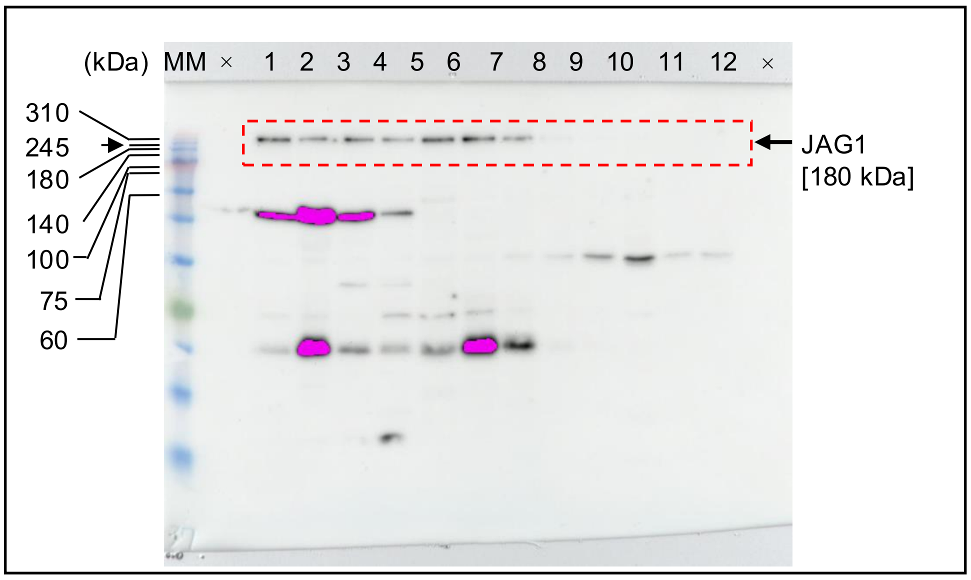

Lane1: Input
Lane2: Fraction 1
Lane3: Fraction 2
Lane4: Fraction 3
Lane5: Fraction 4
Lane6: Fraction 5
Lane7: Fraction 6
Lane8: Fraction 7
Lane9: Fraction 8
Lane10: Fraction 9
Lane11: Fraction 10
Lane12: Fraction 11

Fig. S8. Full western blot images of Fig. 1E (continued)

Journal of Cell Science • Supplementary information

Fig. 3A

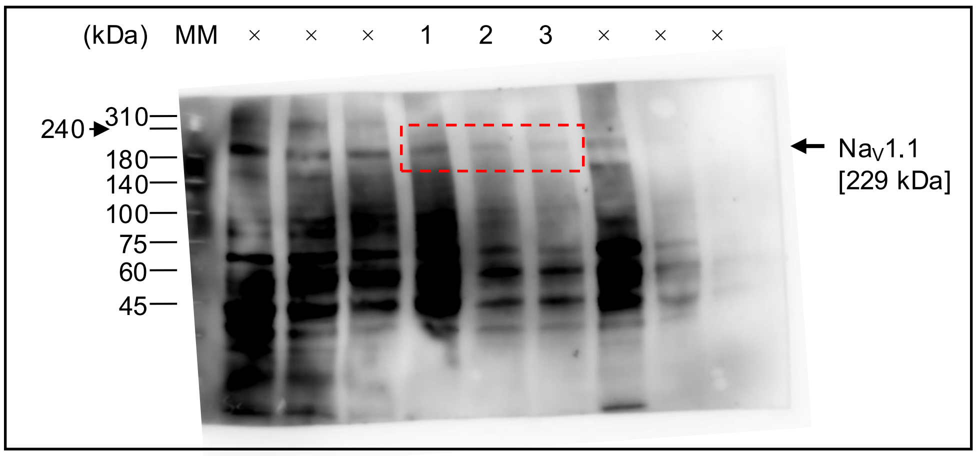

Lane 1: siCTRL
Lane 2: siSCN1A-1
Lane 3: siSCN1A-2
MW: molecular weight

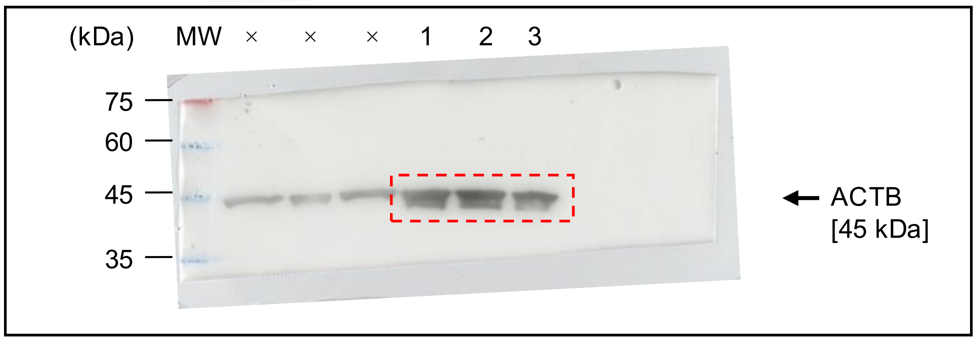

Lane 1: siCTRL
Lane 2: siSCN1A-1
Lane 3: siSCN1A-2
MW: molecular weight

Fig. S9. Full western blot images of Fig. 3A

Journal of Cell Science • Supplementary information

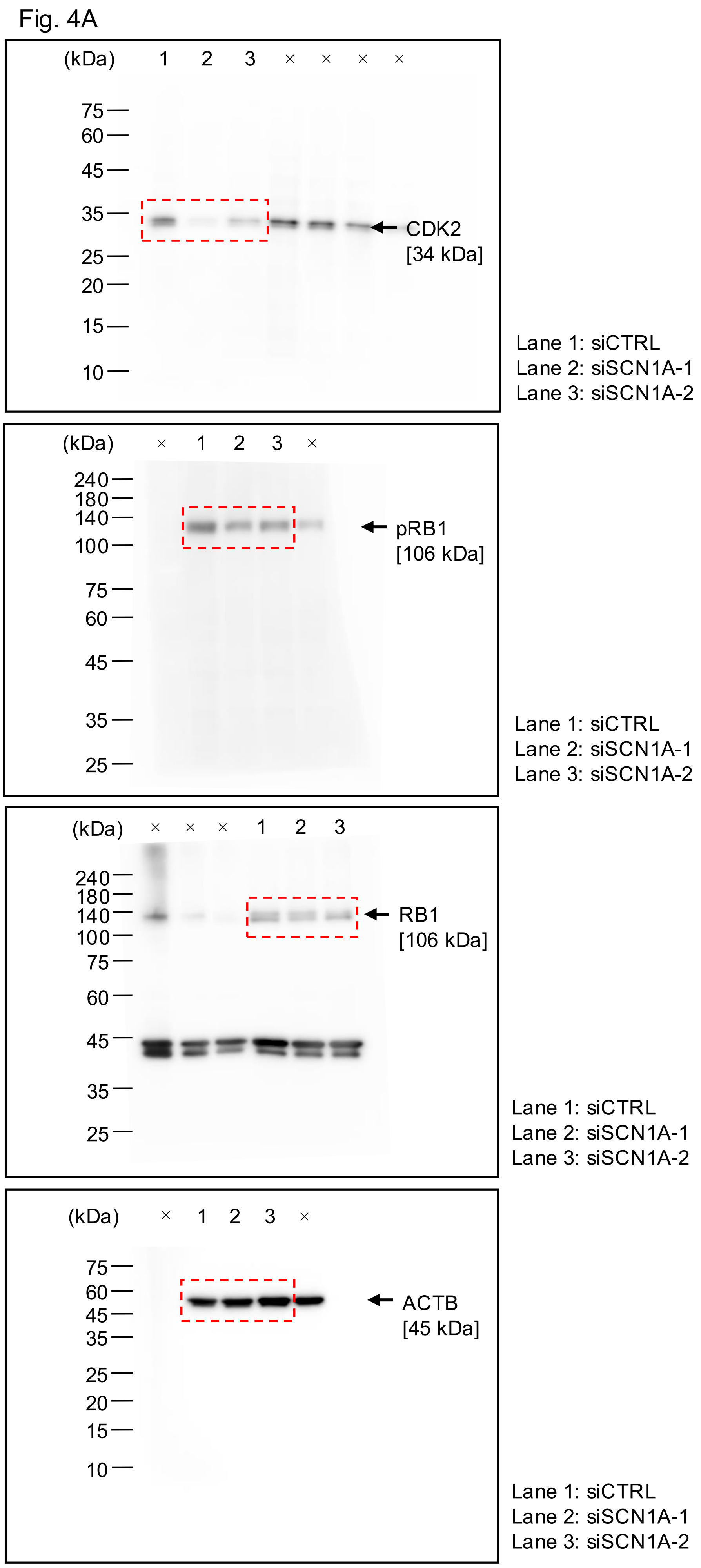

Fig. S10. Full western blot images of Fig. 4A

Journal of Cell Science • Supplementary information

Fig. 4D

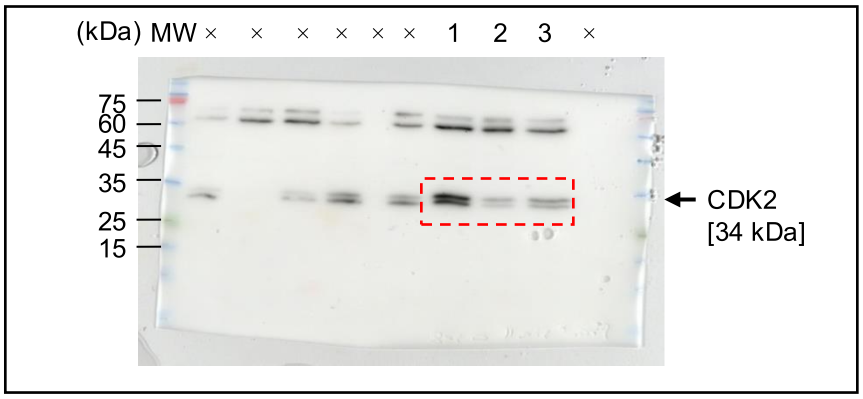

Lane 1: siCTRL
Lane 2: siSCN1A-1
Lane 3: siSCN1A-2
MW: molecular weight

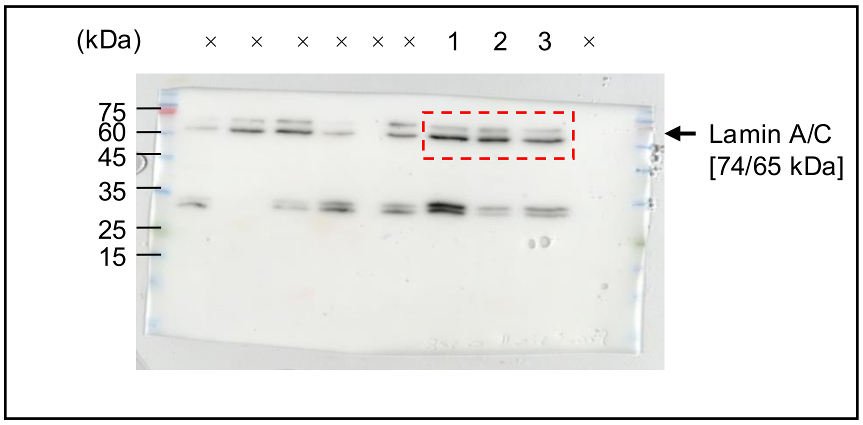

Lane 1: siCTRL
Lane 2: siSCN1A-1
Lane 3: siSCN1A-2
MW: molecular weight

Fig. S11. Full western blot images of Fig. 4D

Fig. 4E

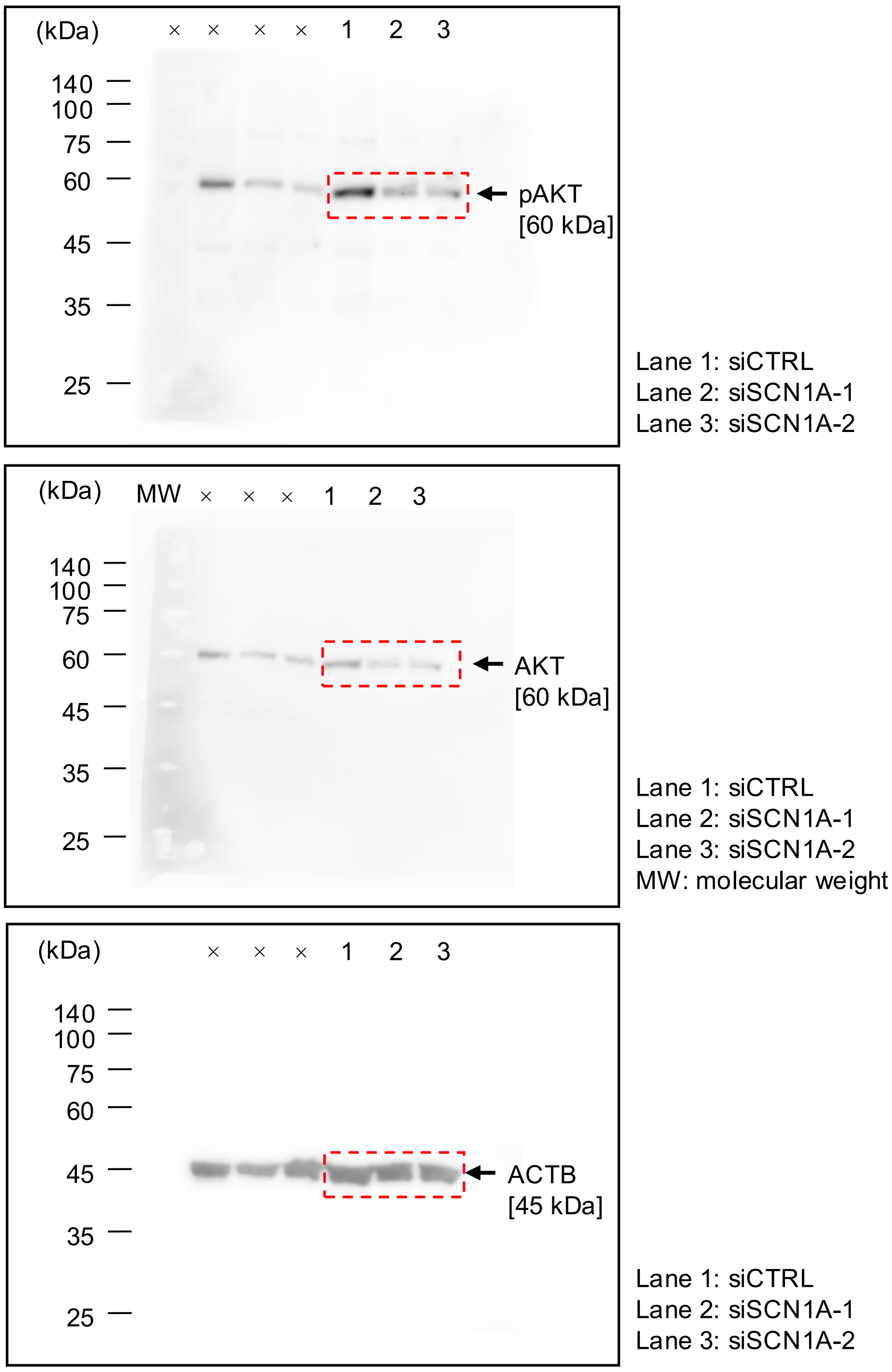

Fig. S12. Full western blot images of Fig. 4E

Journal of Cell Science • Supplementary information

Fig. 5A

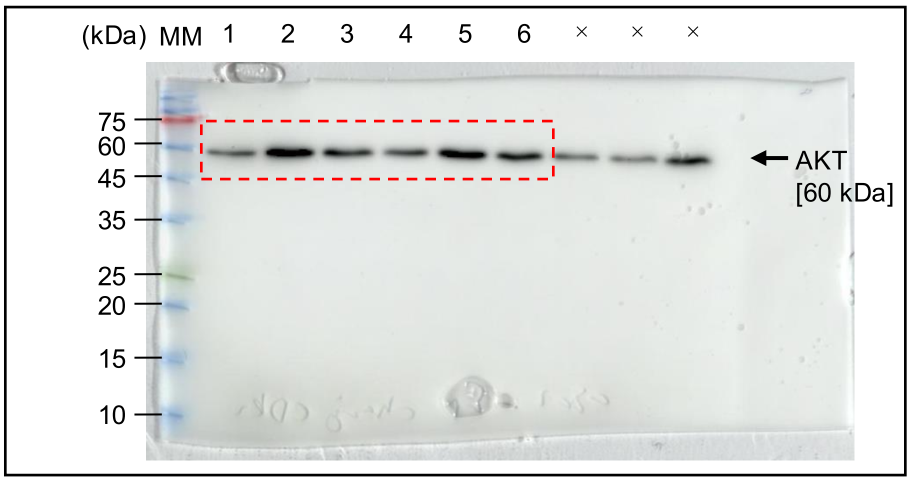

Lane 1: siCTRL CHX+MOCK 0h
Lane 2: siCTRL CHX+MOCK 4h
Lane 3: siCTRL CHX+MOCK 8h
Lane 4: siSCN1A-1 CHX+MOCK 0h
Lane 5: siSCN1A-1 CHX+MOCK 4h
Lane 6: siSCN1A-1 CHX+MOCK 8h
MW: molecular weight

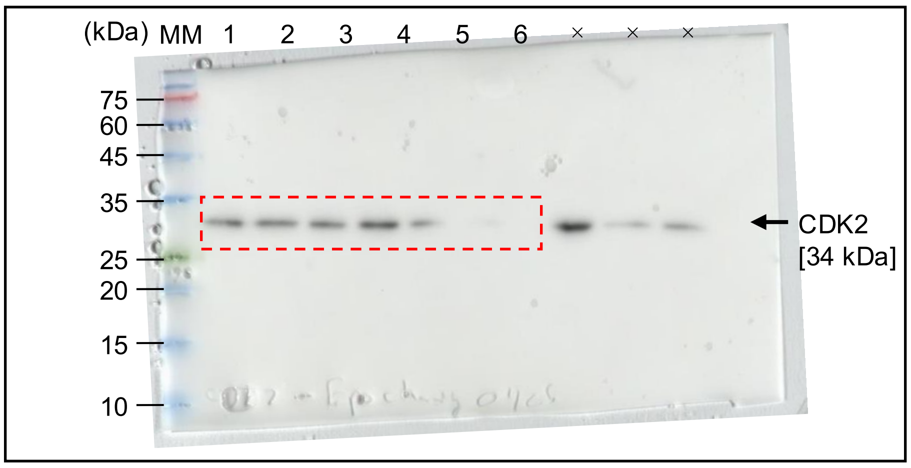

Lane 1: siCTRL CHX+MOCK 0h
Lane 2: siCTRL CHX+MOCK 4h
Lane 3: siCTRL CHX+MOCK 8h
Lane 4: siSCN1A-1 CHX+MOCK 0h
Lane 5: siSCN1A-1 CHX+MOCK 4h
Lane 6: siSCN1A-1 CHX+MOCK 8h
MW: molecular weight

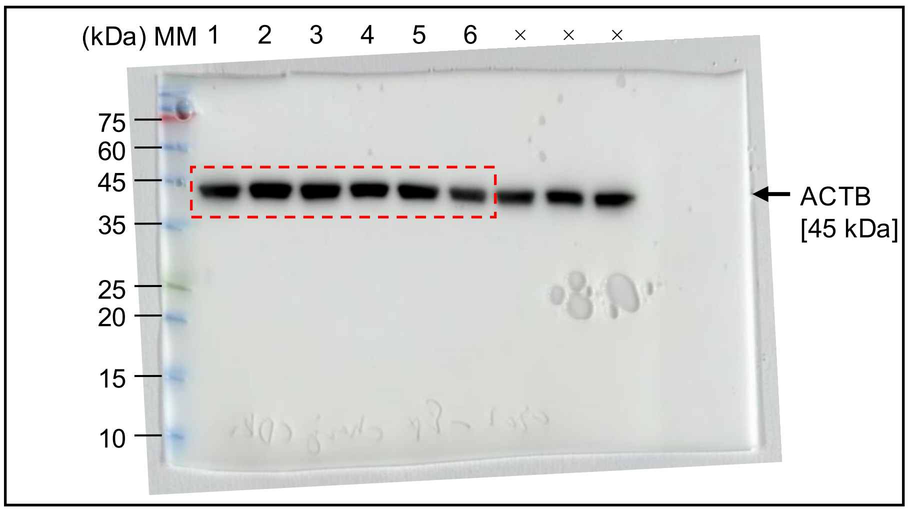

Lane 1: siCTRL CHX+MOCK 0h
Lane 2: siCTRL CHX+MOCK 4h
Lane 3: siCTRL CHX+MOCK 8h
Lane 4: siSCN1A-1 CHX+MOCK 0h
Lane 5: siSCN1A-1 CHX+MOCK 4h
Lane 6: siSCN1A-1 CHX+MOCK 8h
MW: molecular weight

Fig. S13. Full western blot images of Fig. 5A

Journal of Cell Science • Supplementary information

Fig. 5A continued

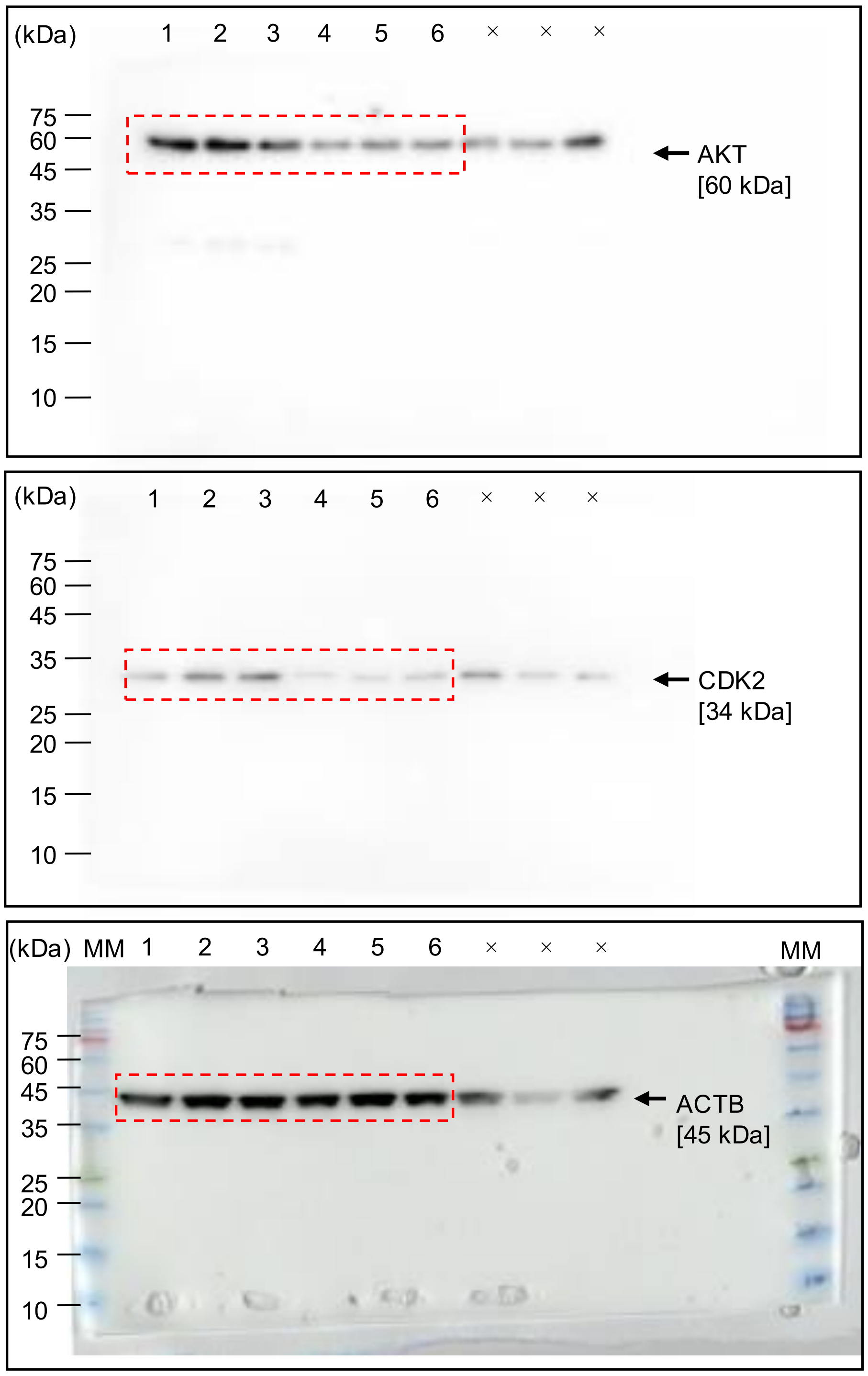

Lane 1: siCTRL CHX+EPOX 0h
Lane 2: siCTRL CHX+EPOX 4h
Lane 3 : siCTRL CHX+EPOX 8h
Lane 4: siSCN1A-1 CHX+EPOX 0h
Lane 5: siSCN1A-1 CHX+EPOX 4h
Lane 6: siSCN1A-1 CHX+EPOX 8h

Lane 1: siCTRL CHX+EPOX 0h
Lane 2: siCTRL CHX+EPOX 4h
Lane 3 : siCTRL CHX+EPOX 8h
Lane 4: siSCN1A-1 CHX+EPOX 0h
Lane 5: siSCN1A-1 CHX+EPOX 4h
Lane 6: siSCN1A-1 CHX+EPOX 8h

Lane 1: siCTRL CHX+EPOX 0h
Lane 2: siCTRL CHX+EPOX 4h
Lane 3 : siCTRL CHX+EPOX 8h
Lane 4: siSCN1A-1 CHX+EPOX 0h
Lane 5: siSCN1A-1 CHX+EPOX 4h
Lane 6: siSCN1A-1 CHX+EPOX 8h
MW: molecular weight

Fig. S14. Full western blot images of Fig. 5A (continued)

Fig. 5B

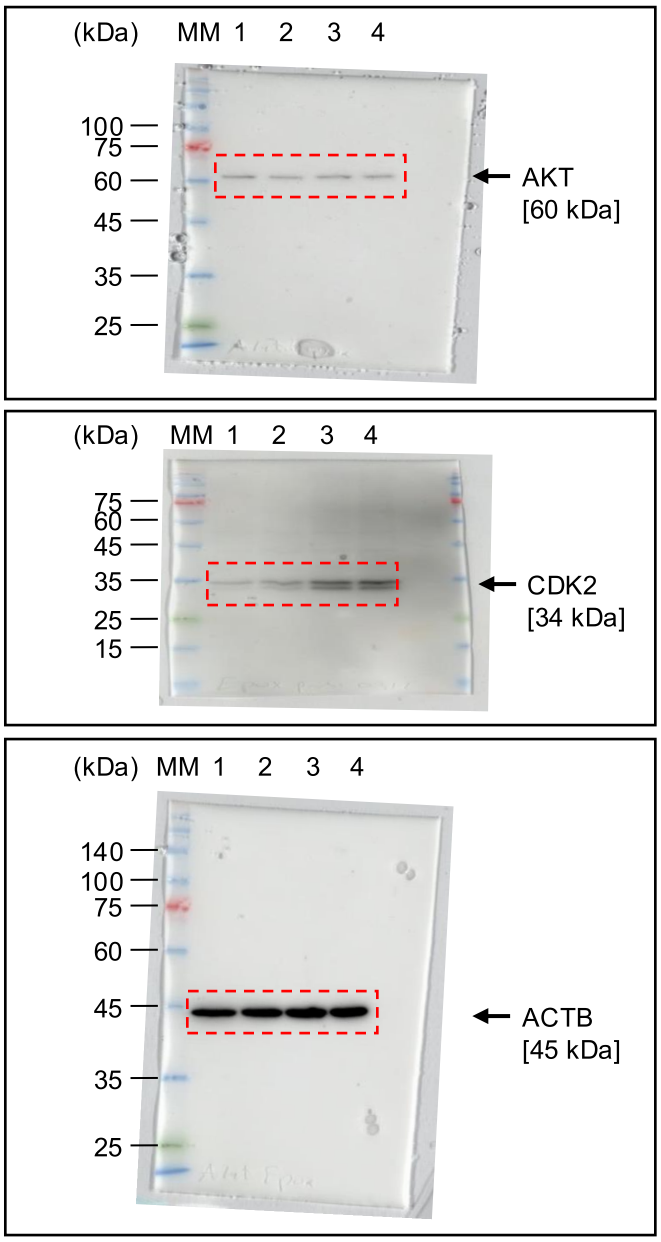

Lane 1: siSCN1A-1+MOCK
Lane 2: siSCN1A-1+EPOX
Lane 3: siSCN1A-2+MOCK
Lane 4: siSCN1A-2+EPOX
MW: molecular weight

Lane 1: siSCN1A-1+MOCK
Lane 2: siSCN1A-1+EPOX
Lane 3: siSCN1A-2+MOCK
Lane 4: siSCN1A-2+EPOX
MW: molecular weight

Lane 1: siSCN1A-1+MOCK
Lane 2: siSCN1A-1+EPOX
Lane 3: siSCN1A-2+MOCK
Lane 4: siSCN1A-2+EPOX
MW: molecular weight

Fig. S15. Full western blot images of Fig. 5B

Journal of Cell Science • Supplementary information

Fig. 5C

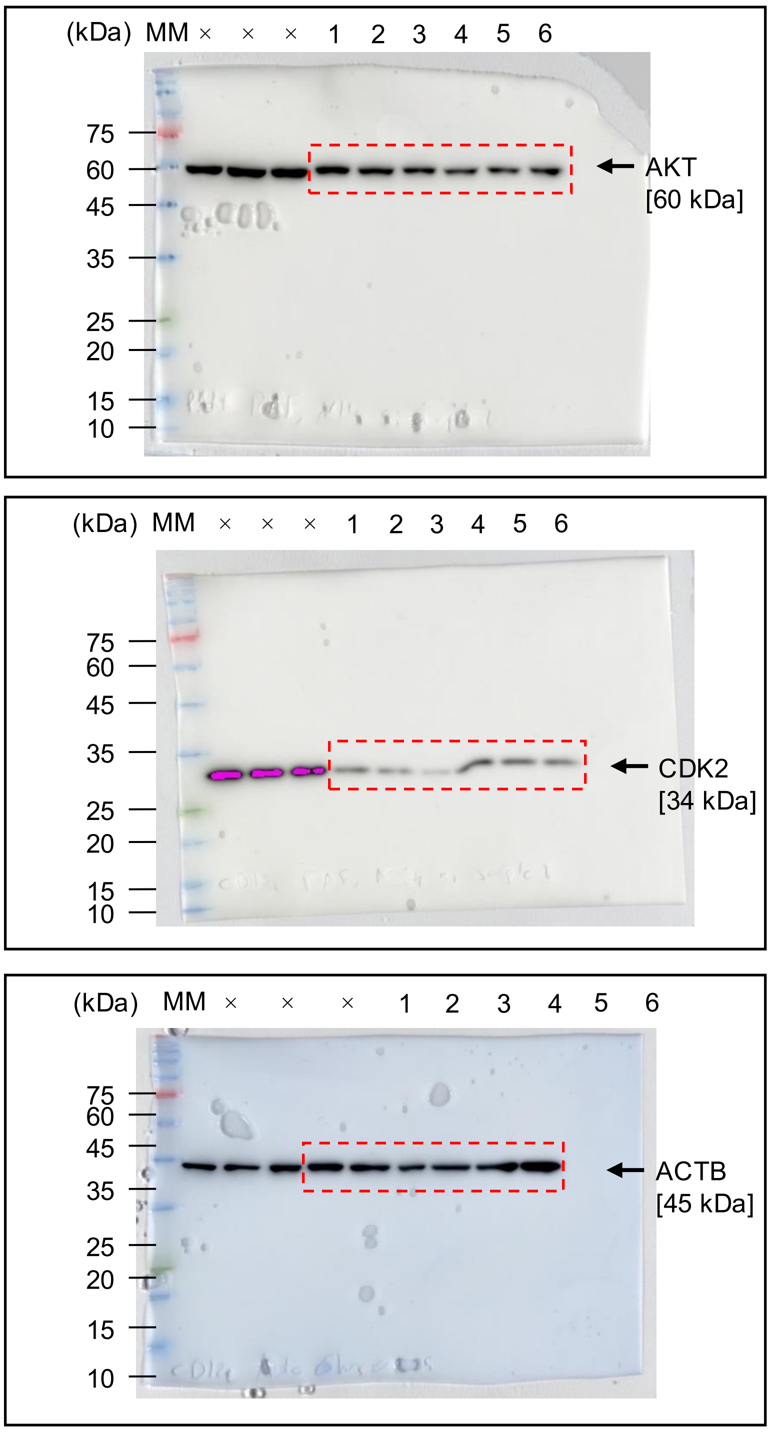

Lane 1: siSCN1A-1+Mock
Lane 2: siSCN1A-1+BAF
Lane 3: siSCN1A-1+NH4Cl
Lane 4: siSCN1A-2+Mock
Lane 5: siSCN1A-2+BAF
Lane 6: siSCN1A-2+NH4Cl
MW: molecular weight

Lane 1: siSCN1A-1+Mock
Lane 2: siSCN1A-1+BAF
Lane 3: siSCN1A-1+NH4Cl
Lane 4: siSCN1A-2+Mock
Lane 5: siSCN1A-2+BAF
Lane 6: siSCN1A-2+NH4Cl
MW: molecular weight

Lane 1: siSCN1A-1+Mock
Lane 2: siSCN1A-1+BAF
Lane 3: siSCN1A-1+NH4Cl
Lane 4: siSCN1A-2+Mock
Lane 5: siSCN1A-2+BAF
Lane 6: siSCN1A-2+NH4Cl
MW: molecular weight

Fig. S16. Full western blot images of Fig. 5C

Journal of Cell Science • Supplementary information

Fig. 6B (siCTRL)

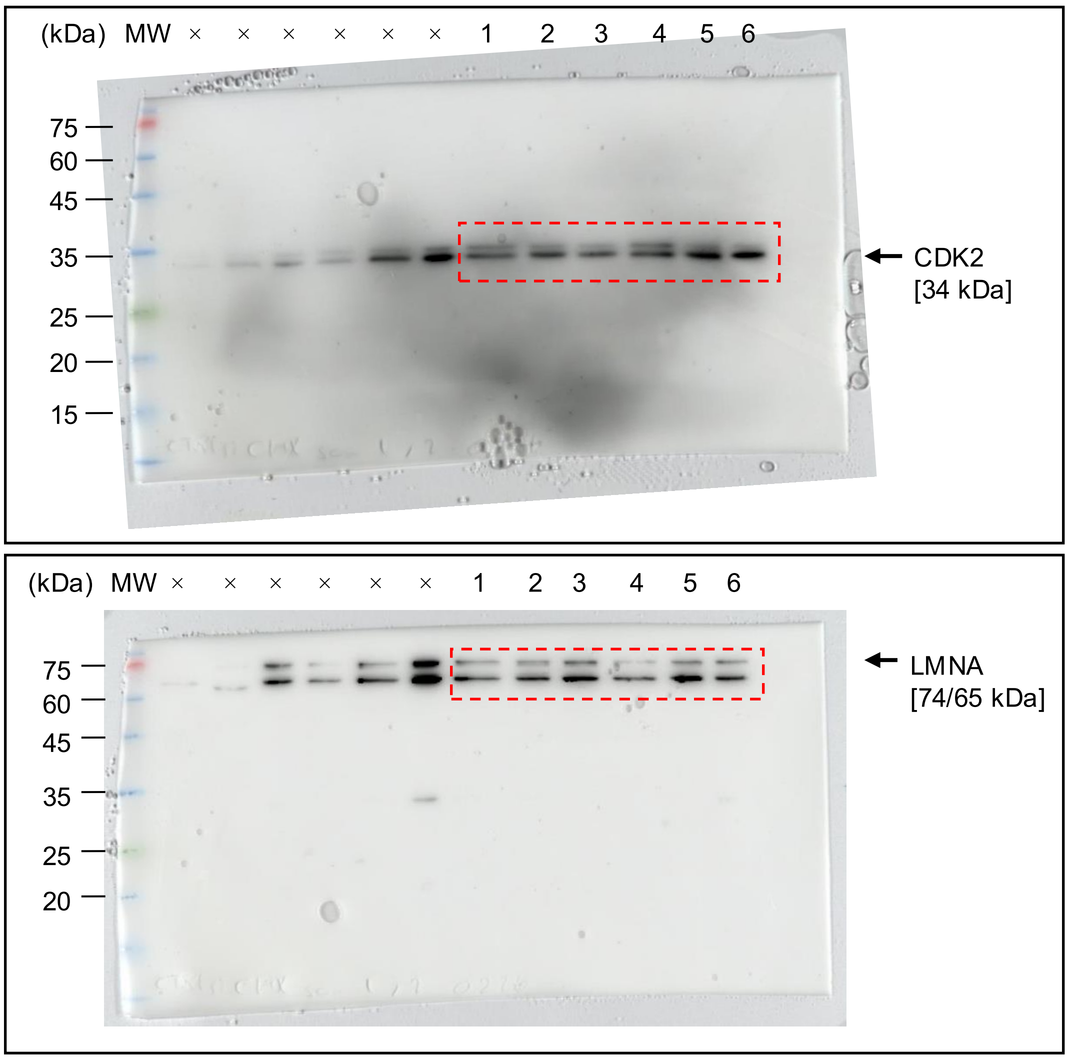

Lane 1: CHX+MOCK 0h
Lane 2: CHX+MOCK 4h
Lane 3: CHX+MOCK 8h
Lane 4: CHX+EPOX 0h
Lane 5: CHX+EPOX 4h
Lane 6: CHX+EPOX 8h
MW: molecular weight

Lane 1: CHX+MOCK 0h
Lane 2: CHX+MOCK 4h
Lane 3: CHX+MOCK 8h
Lane 4: CHX+EPOX 0h
Lane 5: CHX+EPOX 4h
Lane 6: CHX+EPOX 8h
MW: molecular weight

Fig. S17. Full western blot images of Fig. 6B

Fig.6B continued 1 (siSCN1A-1)

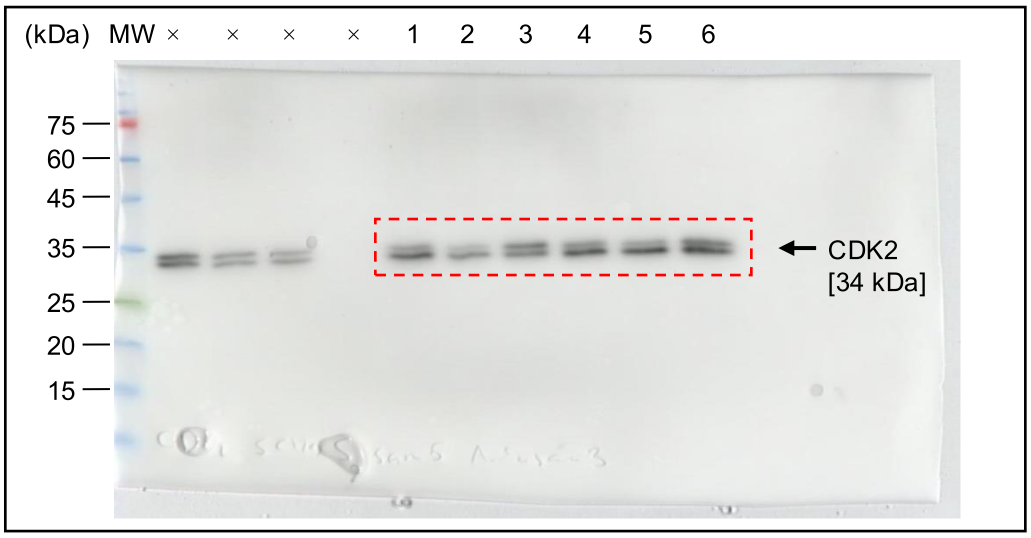

Lane 1: CHX+MOCK 0h
Lane 2: CHX+MOCK 4h
Lane 3: CHX+MOCK 8h
Lane 4: CHX+EPOX 0h
Lane 5: CHX+EPOX 4h
Lane 6: CHX+EPOX 8h
MW: molecular weight

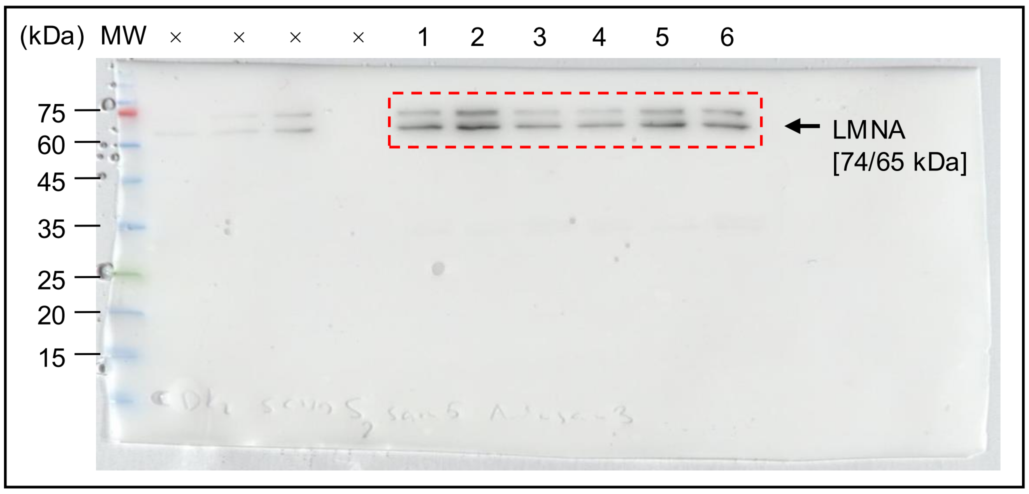

Lane 1: CHX+MOCK 0h
Lane 2: CHX+MOCK 4h
Lane 3: CHX+MOCK 8h
Lane 4: CHX+EPOX 0h
Lane 5: CHX+EPOX 4h
Lane 6: CHX+EPOX 8h
MW: molecular weight

Fig. S18. Full western blot images of Fig. 6B (continued 1)

Journal of Cell Science • Supplementary information

Fig.6B continued 2 (siSCN1A-2)

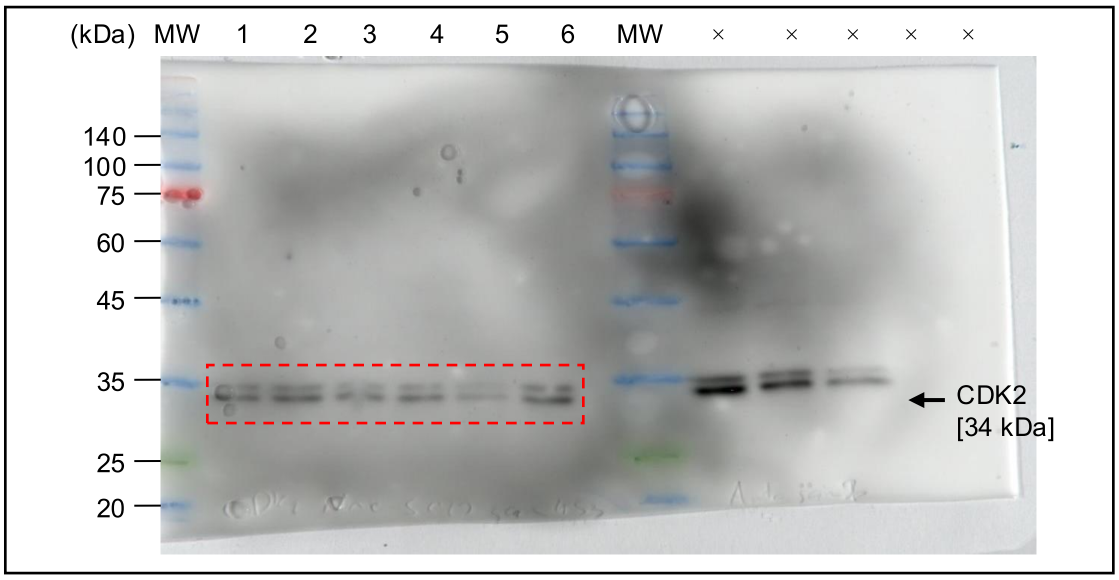

Lane 1: CHX+MOCK 0h
Lane 2: CHX+MOCK 4h
Lane 3: CHX+MOCK 8h
Lane 4: CHX+EPOX 0h
Lane 5: CHX+EPOX 4h
Lane 6: sCHX+EPOX 8h
MW: molecular weight

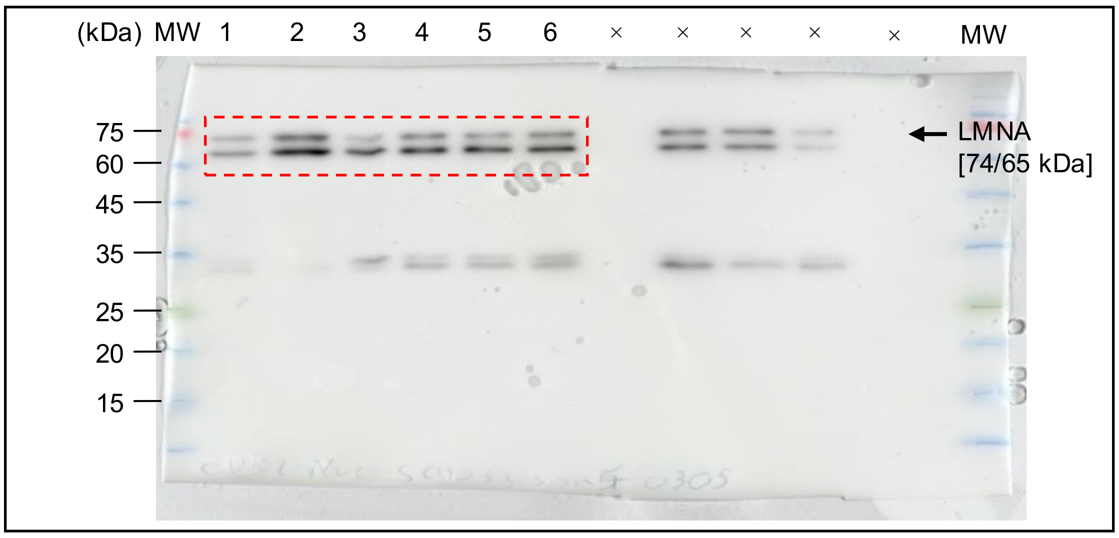

Lane 1: CHX+MOCK 0h
Lane 2: CHX+MOCK 4h
Lane 3: CHX+MOCK 8h
Lane 4: CHX+EPOX 0h
Lane 5: CHX+EPOX 4h
Lane 6: sCHX+EPOX 8h
MW: molecular weight

Fig. S19. Full western blot images of Fig. 6B (continued 2)

Journal of Cell Science • Supplementary information

Fig. 6D

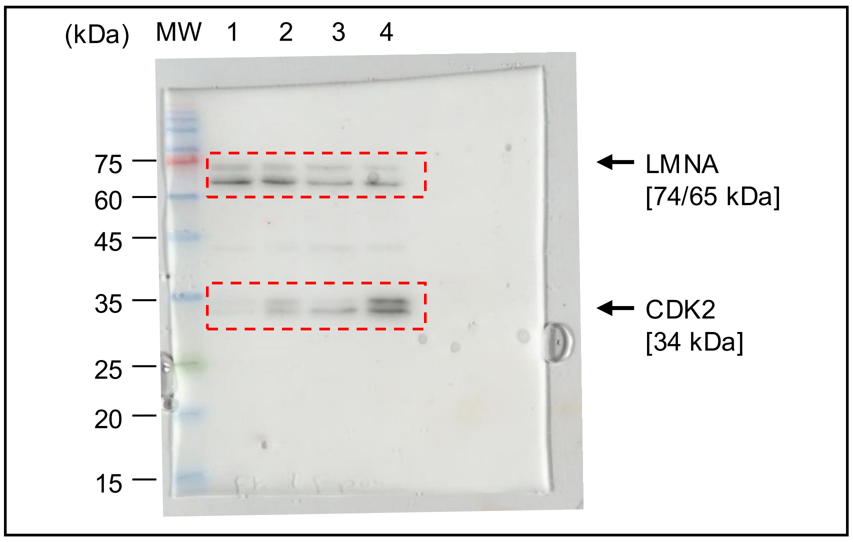

Lane 1: siSCN1A-1+MOCK 8h
Lane 2: siSCN1A-1+Epox 8h
Lane 3: siSCN1A-2+MOCK 8h
Lane 4: siSCN1A-2+Epox 8h
MW: molecular weight

Fig. S20. Full western blot images of Fig. 6D

J. Cell Sci.: doi:10.1242/jcs.261732: Supplementary information

Fig. 6F

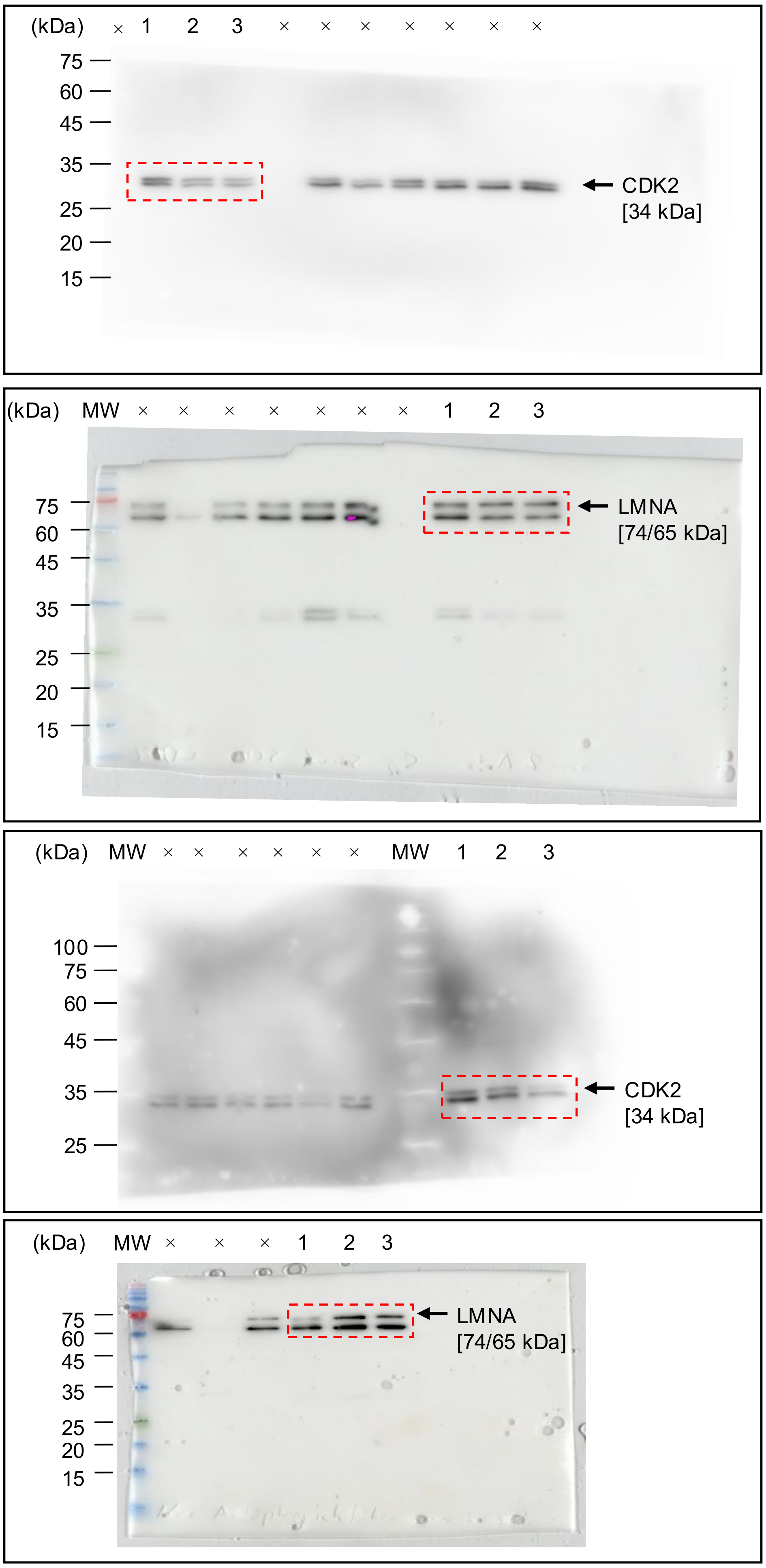

Lane 1: siSCN1A-2+MOCK 6h
Lane 2: siSCN1A-2+BAF 6h
Lane 3: siSCN1A-2+NH_4Cl 6h

Lane 1: siSCN1A-1+MOCK 6h
Lane 2: siSCN1A-1+BAF 6h
Lane 3: siSCN1A-1+NH_4Cl 6h
MW: molecular weight

Lane 1: siSCN1A-2+MOCK 6h
Lane 2: siSCN1A-2+BAF 6h
Lane 3: siSCN1A-2+NH_4Cl 6h
MW: molecular weight

Lane 1: siSCN1A-2+MOCK 6h
Lane 2: siSCN1A-2+BAF 6h
Lane 3: siSCN1A-2+NH_4Cl 6h
MW: molecular weight

Fig. S21. Full western blot images of Fig. 6F

Journal of Cell Science • Supplementary information

Fig. 7A

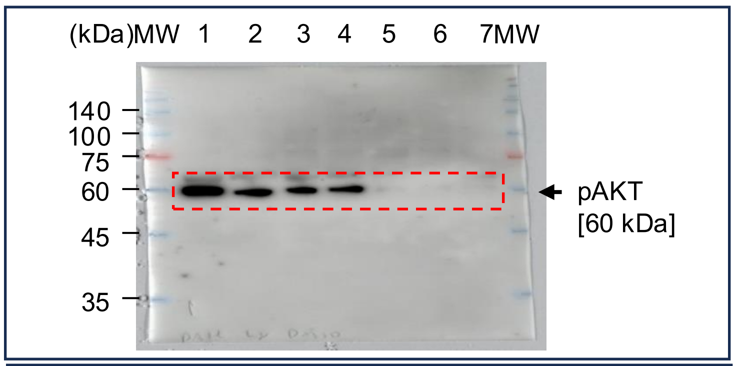

Lane 1: DMSO 0 min
Lane 2: DMSO 5 min
Lane 3: DMSO 10 min
Lane 4: DMSO 20 min
Lane 5: LY 5 min
Lane 6: LY 10 min
Lane 7: LY 20 min
MW: molecular weight

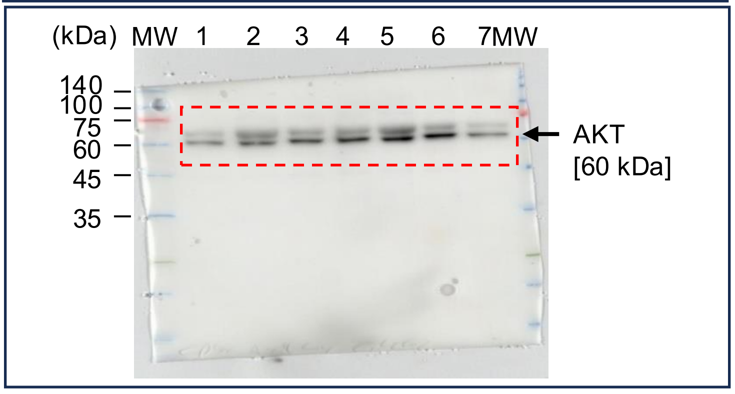

Lane 1: DMSO 0 min
Lane 2: DMSO 5 min
Lane 3: DMSO 10 min
Lane 4: DMSO 20 min
Lane 5: LY 5 min
Lane 6: LY 10 min
Lane 7: LY 20 min
MW: molecular weight

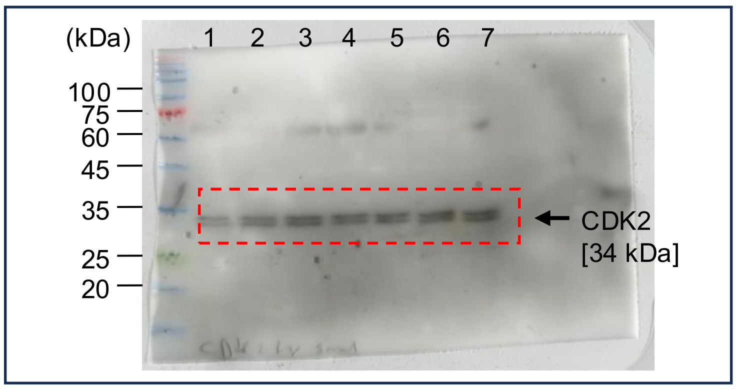

Lane 1: DMSO 0 min
Lane 2: DMSO 5 min
Lane 3: DMSO 10 min
Lane 4: DMSO 20 min
Lane 5: LY 5 min
Lane 6: LY 10 min
Lane 7: LY 20 min
MW: molecular weight

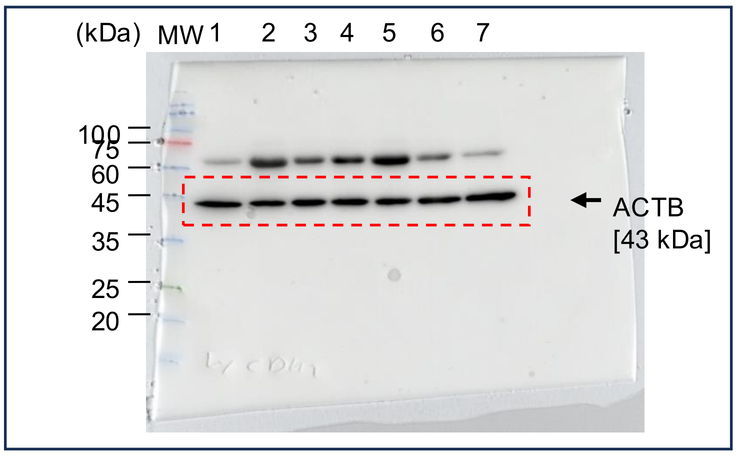

Lane 1: DMSO 0 min
Lane 2: DMSO 5 min
Lane 3: DMSO 10 min
Lane 4: DMSO 20 min
Lane 5: LY 5 min
Lane 6: LY 10 min
Lane 7: LY 20 min
MW: molecular weight

Fig. S22. Full western blot images of Fig. 7A

Journal of Cell Science • Supplementary information

Fig. 7C

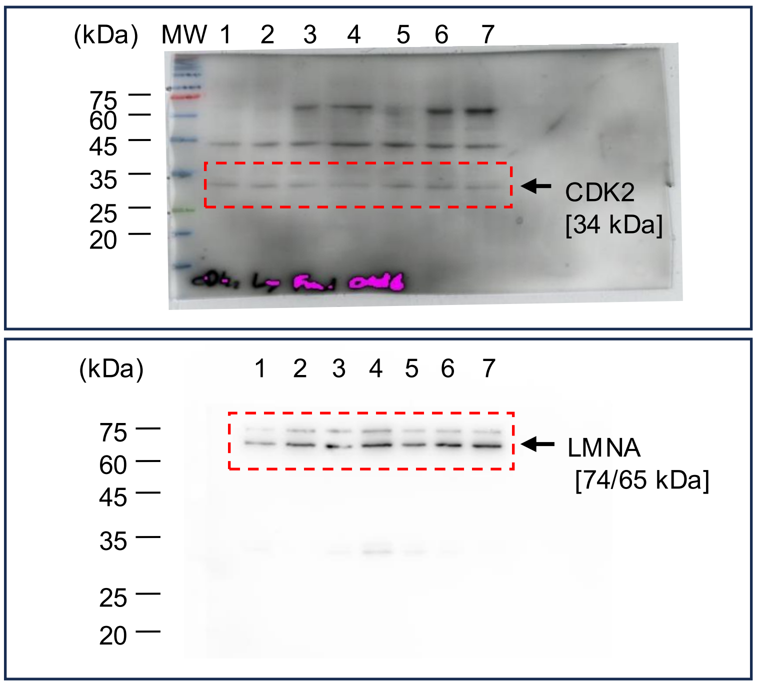

Lane 1: DMSO 0 min
Lane 2: DMSO 5 min
Lane 3: DMSO 10 min
Lane 4: DMSO 20 min
Lane 5: LY 5 min
Lane 6: LY 10 min
Lane 7: LY 20 min
MW: molecular weight

Lane 1: DMSO 0 min
Lane 2: DMSO 5 min
Lane 3: DMSO 10 min
Lane 4: DMSO 20 min
Lane 5: LY 5 min
Lane 6: LY 10 min
Lane 7: LY 20 min

Fig. S23. Full western blot images of Fig. 7C

Fig. 8A

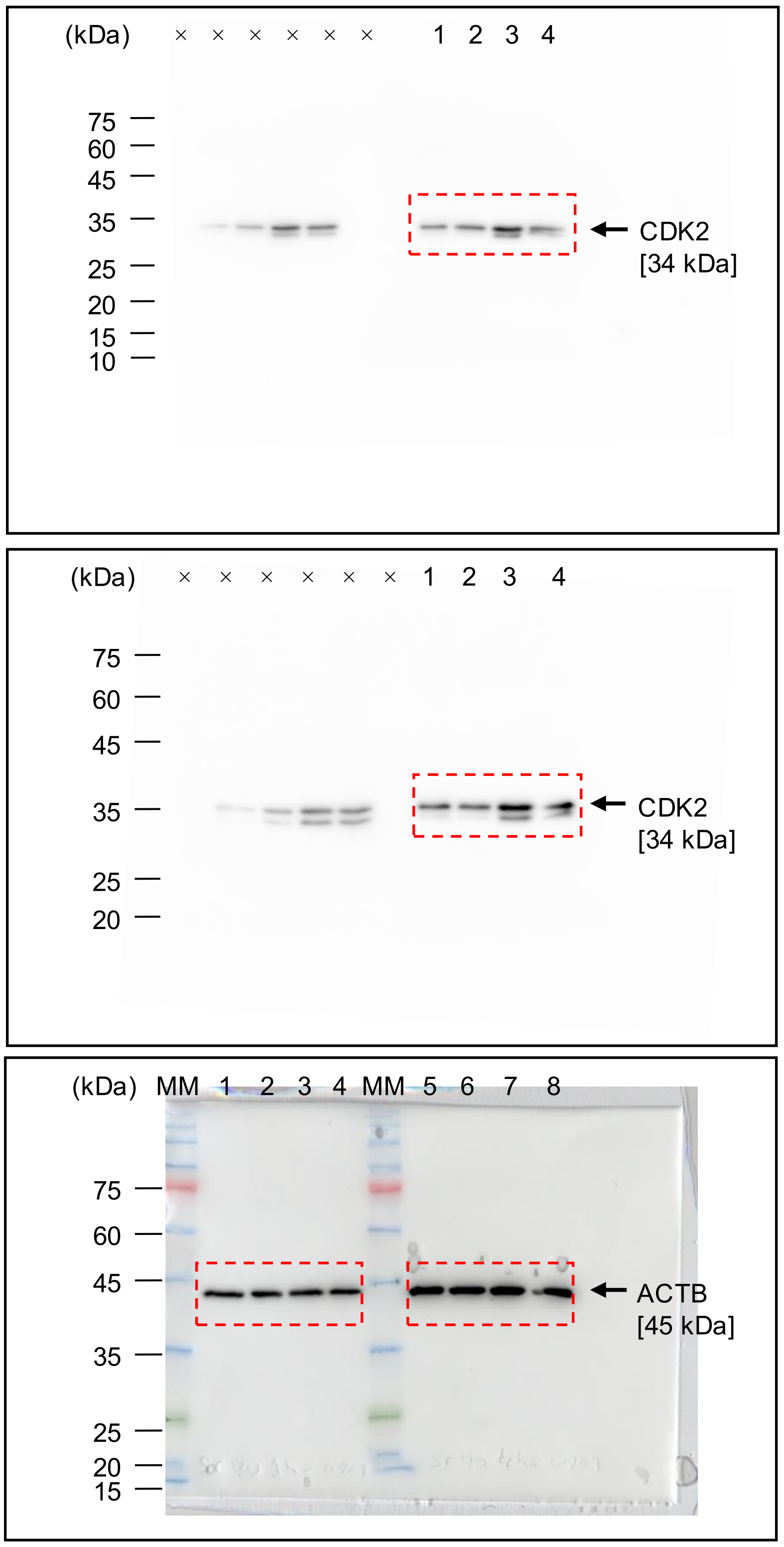

Lane 1: siSCN1A-1 MOCK 1h
Lane 2: siSCN1A-1 SC79 1h
Lane 3: siSCN1A-2 MOCK 1h
Lane 4: siSCN1A-2 SC79 1h

Lane 1: siSCN1A-1 MOCK 4h
Lane 2: siSCN1A-1 SC79 4h
Lane 3: siSCN1A-2 MOCK 4h
Lane 4: siSCN1A-2 SC79 4h

Lane 1: siSCN1A-1 MOCK 1h
Lane 2: siSCN1A-1 SC79 1h
Lane 3: siSCN1A-2 MOCK 1h
Lane 4: siSCN1A-2 SC79 1h
Lane 5: siSCN1A-1 MOCK 4h
Lane 6: siSCN1A-1 SC79 4h
Lane 7: siSCN1A-2 MOCK 4h
Lane 8: siSCN1A-2 SC79 4h
MW: molecular weight

Fig. S24. Full western blot images of Fig. 8A

Journal of Cell Science • Supplementary information

Fig. 8C

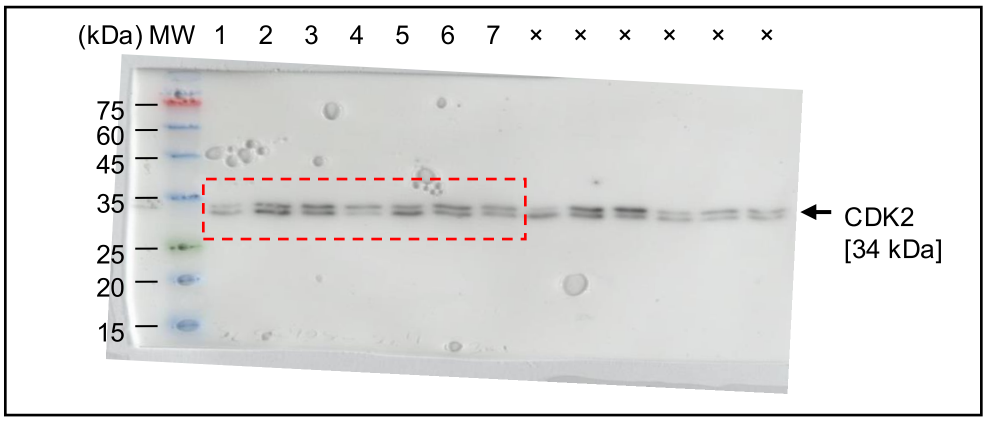

Lane 1: siSCN1A-1 DMSO 0h
Lane 2: siSCN1A-1 DMSO 1h
Lane 3: siSCN1A-1 DMSO 4h
Lane 4: siSCN1A-1 DMSO 24h
Lane 5: siSCN1A-1 SC79 1h
Lane 6: siSCN1A-1 SC79 4h
Lane 7: siSCN1A-1 SC79 24h
MW: molecular weight

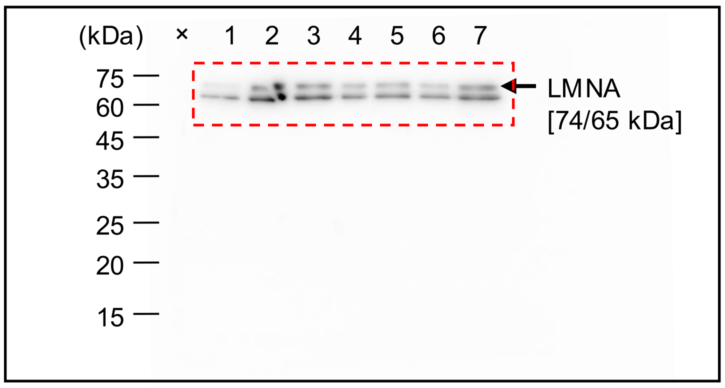

Lane 1: siSCN1A-1 DMSO 0h
Lane 2: siSCN1A-1 DMSO 1h
Lane 3: siSCN1A-1 DMSO 4h
Lane 4: siSCN1A-1 DMSO 24h
Lane 5: siSCN1A-1 SC79 1h
Lane 6: siSCN1A-1 SC79 4h
Lane 7: siSCN1A-1 SC79 24h

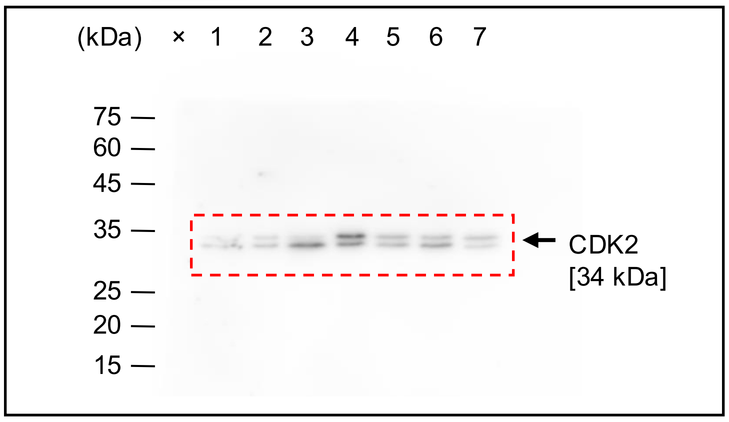

Lane 1: siSCN1A-2 DMSO 0h
Lane 2: siSCN1A-2 DMSO 1h
Lane 3: siSCN1A-2 DMSO 4h
Lane 4: siSCN1A-2 DMSO 24h
Lane 5: siSCN1A-2 SC79 1h
Lane 6: siSCN1A-2 SC79 4h
Lane 7: siSCN1A-2 SC79 24h

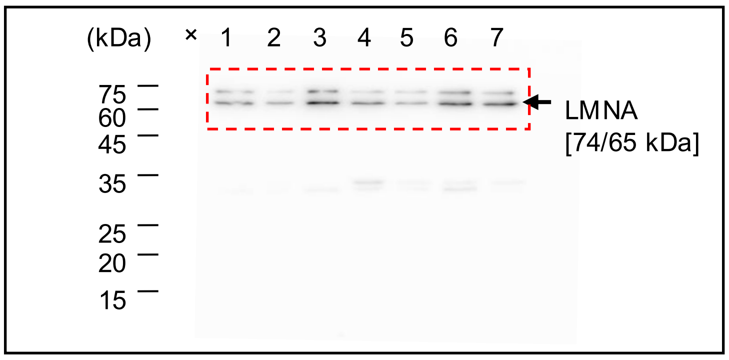

Lane 1: siSCN1A-2 DMSO 0h
Lane 2: siSCN1A-2 DMSO 1h
Lane 3: siSCN1A-2 DMSO 4h
Lane 4: siSCN1A-2 DMSO 24h
Lane 5: siSCN1A-2 SC79 1h
Lane 6: siSCN1A-2 SC79 4h
Lane 7: siSCN1A-2 SC79 24h

Fig. S25. Full western blot images of Fig. 8C

Journal of Cell Science • Supplementary information

Fig. S4

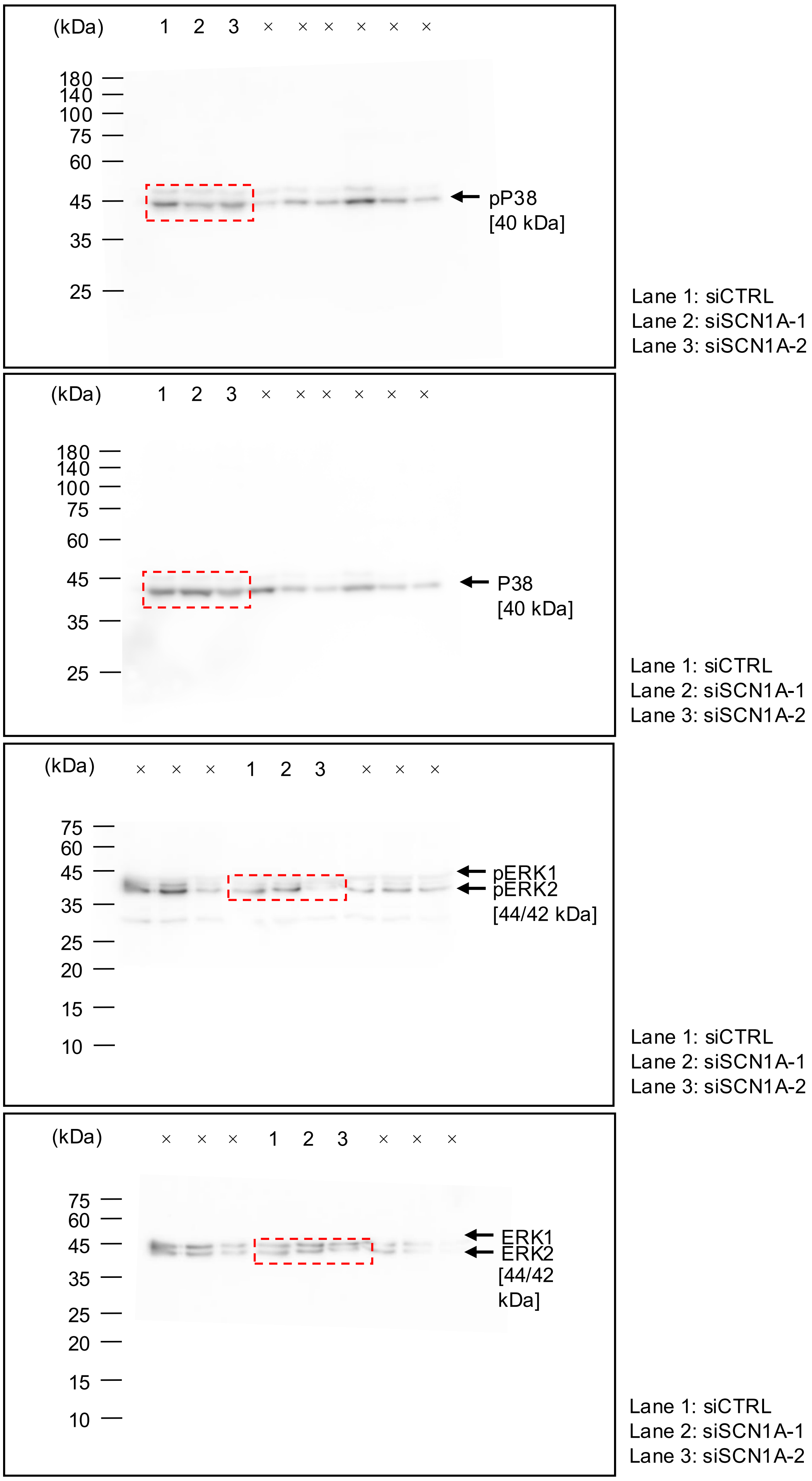

Fig. S26. Full western blot images of Fig. S4

Journal of Cell Science • Supplementary information

Fig. S4 continued 1

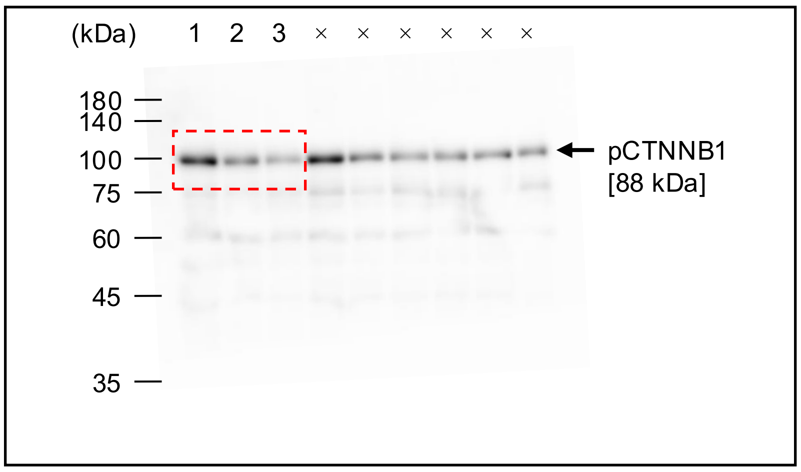

Lane 1: siCTRL
Lane 2: siSCN1A-1
Lane 3: siSCN1A-2

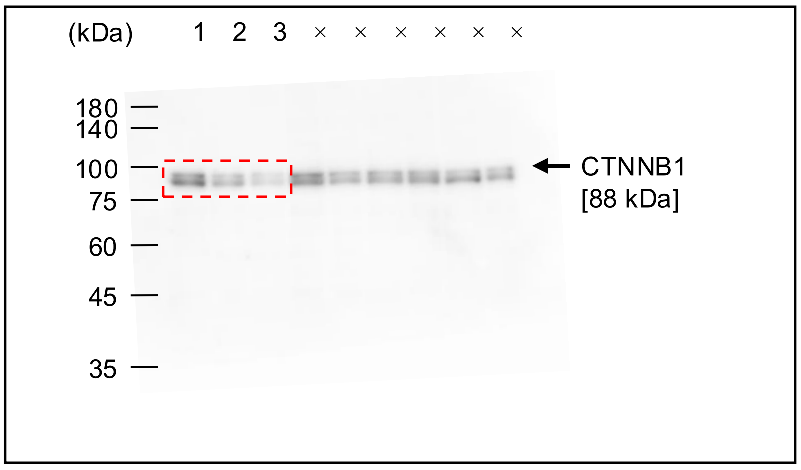

Lane 1: siCTRL
Lane 2: siSCN1A-1
Lane 3: siSCN1A-2

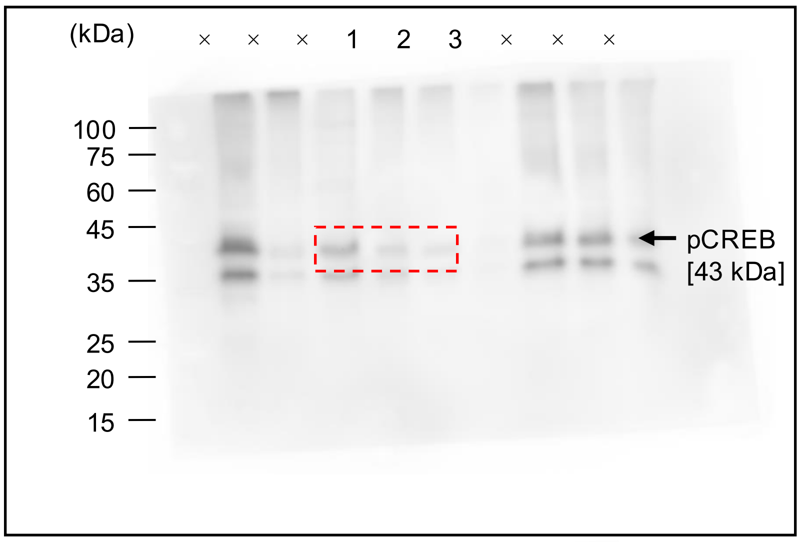

Lane 1: siCTRL
Lane 2: siSCN1A-1
Lane 3: siSCN1A-2

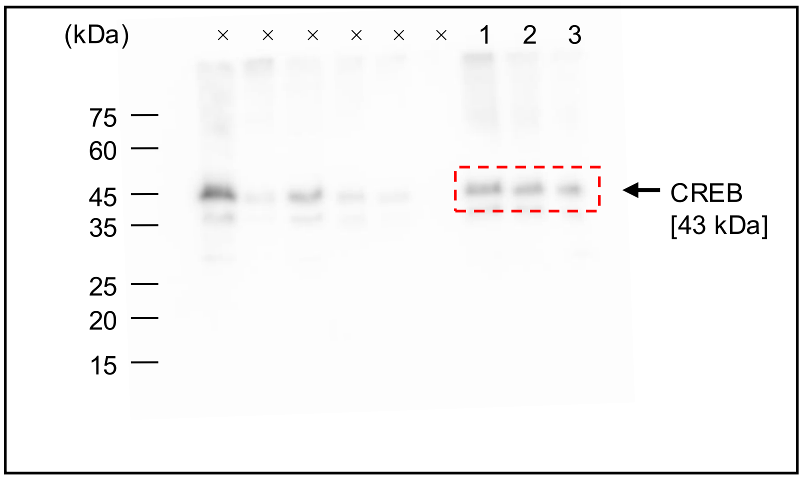

Lane 1: siCTRL
Lane 2: siSCN1A-1
Lane 3: siSCN1A-2

Fig. S27. Full western blot images of Fig. S4 (continued 1)

Journal of Cell Science • Supplementary information

Fig. S4 continued 2

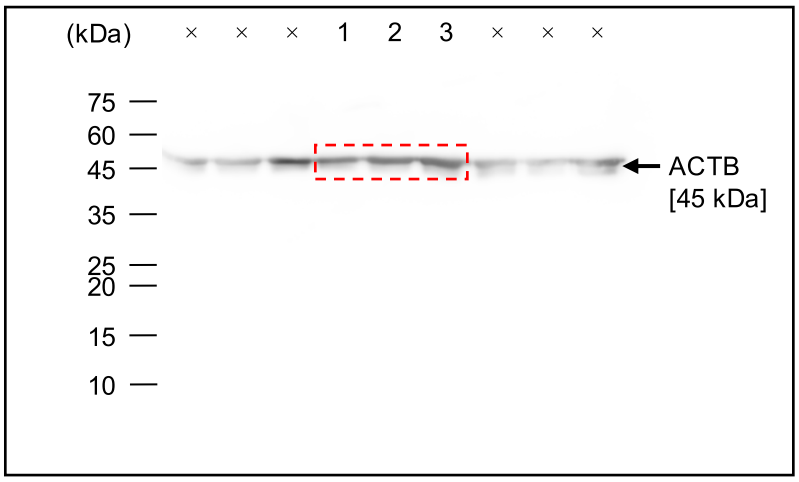

Lane 1: siCTRL
Lane 2: siSCN1A-1
Lane 3: siSCN1A-2

Fig. S28. Full western blot images of Fig. S4 (continued 2)

Table S1. Antibodies used for flow cytometry analysis

Target, Specify	Host, Isotype, Conjugate	Clone	Dilution	Catalog #	Manufacturer (City, State, Country)
CD14, Human	Mouse, IgG1 kappa, R-PE	61D3	1:100	12-0149-42	eBioscience (Waltham, MA, USA)
CD19, Human	Mouse, IgG1 kappa, R-PE	47 G	1:100	302207	Biolegend (San Diego, CA, USA)
CD34, Human	Mouse, IgG2a kappa, R-PE	4H11	1:100	12-0349-42	eBioscience (Waltham, MA, USA)
CD45, Human	Mouse, IgG1 kappa, R-PE	HI30	1:100	12-0459-42	eBioscience (Waltham, MA, USA)
CD73, Human	Mouse, IgG1 kappa, R-PE	AD2	1:100	550257	BD (Franklin Lakes, NJ, USA)
CD90, Human	Mouse, IgG1 kappa, R-PE	5E10	1:100	328110	Biolegend (San Diego, CA, USA)
CD105, Human	Mouse, IgG1 kappa, R-PE	SN6	1:100	12-1057-42	eBioscience (Waltham, MA, USA)
CD146, Human	Mouse, IgG1 kappa, R-PE	P1H112	1:100	550315	BD (Franklin Lakes, NJ, USA)
HLA-DR, Human	Mouse, IgG2a kappa, R-PE	L243	1:100	307606	Biolegend (San Diego, CA, USA)
SSEA-4, Human	Mouse, IgG3 kappa, R-PE	MC-813-70	1:100	FAB1435P	R&D Systems (Minneapolis, MN, USA)
IgG1 Isotype, Mouse	Mouse, IgG1 kappa, R-PE	P3.6.2.8.1	1:100	12-4714-42	eBioscience (Waltham, MA, USA)
IgG2a Isotype, Mouse	Mouse, IgG2a kappa, R-PE	eBM2a	1:100	12-4724-42	eBioscience (Waltham, MA, USA)
IgG3 Isotype, Mouse	Mouse, IgG3 kappa, R-PE	B10	1:100	12-4742-42	eBioscience (Waltham, MA, USA)

HLA-DR, human leukocyte antigen DR; SSEA-4, stage specific antigen4; R-PE, R-Phycoerythin.

Journal of Cell Science • Supplementary information

Table S2. Antibodies used for Western blotting and immunofluorescence

Target	Host, Isotype, Conjugate	Dilution	Clone	Catalog #	Manufacturer (City, State, Country)
Primary Ab					
ACTB	Mouse, mAb, Non	1:5000	AC-15	A5441	Merck (Darmstadt, Germany)
AKT	Rabbit, pAb, Non	1:1000		9272	CST (Danvers, MA, USA)
AKT, Phospho (Ser473)	Rabbit, mAb, Non	1:1000	D9E	4060	CST (Danvers, MA, USA)
CANX	Rabbit, mAb, Non	1:1000	C5C9	2679	CST (Danvers, MA, USA)
CANX	Mouse, mAb, Non	1:100	E-10	sc-46669	SCBT (Dallas, TX, USA)
CD90	Mouse, mAb, Non,	1:100	5E10	328101	BioLegend (San Diego, CA, USA)
CDK2	Rabbit, mAb, Non	1:1000	78B2	2546	CST (Danvers, MA, USA)
CREB	Rabbit, mAb, Non	1:1000	48H2	9197	CST (Danvers, MA, USA)
CREB, Phospho (Ser133)	Rabbit, mAb, Non	1:1000	87G3	9198	CST (Danvers, MA, USA)
CTNNB1	Rabbit, pAb, Non	1:4000		C2206	Merck (Darmstadt, Germany)
CTNNB1, Phospho (Ser675)	Rabbit, mAb, Non	1:1000	D2F1	4176	CST (Danvers, MA, USA))
ERK1/2	Rabbit, pAb, Non	1:1000		9102	CST (Danvers, MA, USA)
ERK1/2, Phospho (Thr202/Tyr204)	Mouse, mAb, Non	1:2000	E10	9106	CST (Danvers, MA, USA)
JAG1	Rabbit, mAb, Non	1:1000	28H8	2620	CST (Danvers, MA, USA)
Nav1.1	Rabbit, pAb, Non	1:1000		ASC001	Alomone (Jerusalem, IL, USA)
P38	Rabbit, pAb, Non	1:1000		9212	CST (Danvers, MA, USA)
P38, Phospho (Thr180/Tyr182)	Rabbit, pAb, Non	1:1000		9211	CST (Danvers, MA, USA)
RB	Rabbit, mAb, Non	1:1000	4H1	9309	CST (Danvers, MA, USA)
RB, Phospho (Ser780)	Rabbit, mAb, Non	1:1000	D59B7	8180	CST (Danvers, MA, USA)
SRC	Rabbit, mAb, Non	1:1000	32G6	2123	CST (Danvers, MA, USA)
SRC, Phospho (Try529)	Rabbit, mAb, Non	1:5000	Y232	ab32078	Abcam (Cambridge, UK)
LMNA	Rabbit, pAb, Non	1:1000		2032	CST (Danvers, MA, USA)
Secondary Ab					
Mouse IgG, for WB	Goat, pAb, HRP	1:5000		sc-2005	SCBT (Dallas, TX, USA)
Mouse IgG, for IF	Goat, pAb, AF488	1:500		AB_2338840	JIR (West Grove, PA, USA)
Mouse IgG, for IF	Goat, pAb, AF594	1:500		AB_2338871	JIR (West Grove, PA, USA)
Rabbit IgG, for WB	Goat, pAb, HRP	1:5000		sc-2004	SCBT (Dallas, TX, USA)
Rabbit IgG, for IF	Goat, pAb, AF488	1:500		AB_2338046	JIR (West Grove, PA, USA)
Rabbit IgG, for IF	Goat, pAb, AF594	1:500		AB_2632469	JIR (West Grove, PA, USA)

Ab antibody, mAb, monoclonal antibody, pAb, polyclonal antibody. ACTB, actin beta; CANX, calnexin; CDK, cyclin dependent kinase, CDKN, cyclin dependent kinase inhibitor; CREB, cAMP response element binding protein; CTNNB1, catenin beta1; ERK1/2, extracellular signal related kinase 1 and 2; JAG1, Jagged canonical Notch ligand 1; Nav1.1, sodium voltage-gated channel alpha subunit 1; RB, retinoblastoma protein; SRC, proto-oncogene, non-receptor tyrosine kinase; LMNA, lamin A/C. Ser, serine; Thr, threonine; Tyr, tyrosine; Y, tyrosine. IF, immunofluorescence; WB, Western blotting. AF, Alexa Fluor; HRP, horse radish peroxidase.
CST, Cell Signaling Technology; JIR, Jackson ImmunoResearch; SCBT, Santa Cruz Biotechnology.

Journal of Cell Science • Supplementary information

Table S3. Primer sequences used for quantitative polymerase chain reaction

Gene	Forward primer	Reverse Primer	Manufacturer (City, State, Country)
ADIPOQ	5' CCCTCTCTTACAAGCCCATCA 3'	5' GAGCCAGTCTGGTAGTACATCA 3'	TMO (Waltham, MA, USA)
AKT1	5' ACTGTCATCGAACGCACCTT 3'	5' TTCAGCCCCTGAGTTGTCAC 3'	TMO (Waltham, MA, USA)
ALP	5' ACGTGGCTAAGAATGTCATC 3'	5' CTGGTAGGCGATGTCCTTA 3'	TMO (Waltham, MA, USA)
BGLAP	5' AARCCGGACTGTGACGAGTT 3'	5' CAGCAGAGCGACACCCTAGA 3'	TMO (Waltham, MA, USA)
CDK2	5' TGGCGCTTCATGGAGAACTT 3'	5' CAGTGAGAGCAGAGGCATCC 3'	TMO (Waltham, MA, USA)
CEBPA	5' TTCACATTGCACAAGGCACT 3'	5' GAGGGACCGGAGTTATGACA 3'	TMO (Waltham, MA, USA)
FABP4	5' GGATGATAAACTGGTGGTGGAATG 3'	5' CAGAATGTTGTAGAGTTCAATGCGA 3'	TMO (Waltham, MA, USA)
RPL13A	5' GCTGTGAAGGCATCAACATTT 3'	5' CATCCGCTTTTTCTTGTCGTA 3'	TMO (Waltham, MA, USA)
RUNX2	5' CAGTTCCCAAGCATTTCATCC 3'	5' TCAATATGGTCGCCAAACAG 3'	TMO (Waltham, MA, USA)
SCN1A	5' GCAATGGTGTGGTTTCCTTGGT 3'	5' TCGTTGCCTTTGGGAAGGATCT 3'	TMO (Waltham, MA, USA)

ADIPOQ, *adiponectin, C1Q and collagen domain containing*; *ALP*, *alkaline phosphatase*; *BGLAP*, *bone gamma-carboxyglutamate protein*; *CDK2*, *cyclin dependent kinase 2*; *CEBPA*, *˜CCAAT enhancer binding protein alpha*; *FABP4*, *fatty acid binding protein 4*; *RPL13A*, *ribosomal protein 13A*; *RUNX2*, *RUNX family transcription factor 2*; *SCN1A*, *sodium voltage-gated channel alpha subunit 1*.
TMO, Thermo Fisher Scientific.

Table S4. TaqMan probes used for quantitative polymerase chain reaction

Gene	TaqMan number	Manufacturer (City, State, Country)
ACAN	Hs00153936_m1	TMO (Waltham, MA, USA)
COL1A1	Hs00164004_m1	TMO (Waltham, MA, USA)
SOX9	Hs01001343_g1	TMO (Waltham, MA, USA)
18S rRNA	Hs99999901_s1	TMO (Waltham, MA, USA)

ACAN, *aggrecan*; *COL2A1*, *collagen type II alpha 1 chain*; *SOX9*, *SRY-box transcription factor 9*.
TMO, Thermo Fisher Scientific.

Journal of Cell Science • Supplementary information
