# Supplementary material for: NaV1.1 contributes to the cell cycle of human mesenchymal stem cells by regulating AKT and CDK2
Source: J Cell Sci. 2024 Oct 10;137(19):jcs261732. doi: 10.1242/jcs.261732 (PMC11491812; doi:10.1242/jcs.261732)
Supplement: Supplementary file 1 [file joces-137-261732_review_history.pdf]

Journal of Cell Science

$Na_V1.1$ contributes to the cell cycle of human mesenchymal stem cells by regulating AKT and CDK2

Mohammed Fouad Zakaria, Hiroki Kato, Soichiro Sonoda, Kenichi Kato, Norihisa Uehara, Yukari Kyumoto-Nakamura, Mohammed Majd Sharifa, Liting Yu, Lisha Dai, Haruyoshi Yamaza, Shunichi Kajioka, Fusanori Nishimura and Takayoshi Yamaza
DOI: 10.1242/jcs.261732

Editor: Renata Basto

Review timeline

Original submission:	23 October 2023
Editorial decision:	12 January 2024
First revision received:	29 May 2024
Editorial decision:	1 July 2024
Second revision received:	17 August 2024
Accepted:	28 August 2024

Original submission

First decision letter

MS ID#: JOCES/2023/261732

MS TITLE: Sodium voltage-gated channel ± subunit 1 contributes to the cell cycle of human mesenchymal stem cells by regulating AKT and CDK2

AUTHORS: Mohammed Fouad Zakaria, Hiroki Kato, Soichiro Sonoda, Kenichi Kato, Norihisa Uehara, Yukari Kyumoto-Nakamura, Mohammed Majd Sharifa, Liting Yu, Lisha Dai, Haruyoshi Yamaza, Shunichi Kajioka, Fusanori Nishimura, and Takayoshi Yamaza

We have now reached a decision on the above manuscript.

To see the reviewers' reports and a copy of this decision letter, please go to: https://submit-jcs.biologists.org and click on the 'Manuscripts with Decisions' queue in the Author Area. (Corresponding author only has access to reviews.)

As you will see, the reviewers raise a number of substantial criticisms that prevent me from accepting the paper at this stage. They suggest, however, that a revised version might prove acceptable, if you can address their concerns. If you think that you can deal satisfactorily with the criticisms on revision, I would be pleased to see a revised manuscript. We would then return it to the reviewers.

Please ensure that you clearly highlight all changes made in the revised manuscript. Please avoid using 'Tracked changes' in Word files as these are lost in PDF conversion.

I should be grateful if you would also provide a point-by-point response detailing how you have dealt with the points raised by the reviewers in the 'Response to Reviewers' box. Please attend to all of the reviewers' comments. If you do not agree with any of their criticisms or suggestions please explain clearly why this is so.

© 2024. Published by The Company of Biologists under the terms of the Creative Commons Attribution License (https://creativecommons.org/licenses/by/4.0/).

Reviewer 1

Advance summary and potential significance to field

These conclusions, if correct, offer a novel functionality for voltage-gated sodium channels distinct from classical ion conduction. Substantiation of these conclusions shall require additional experiments.

Comments for the author

This manuscript presents research examining the proliferation of mesenchymal stem cells (MSCs) and claims to define a requirement for intracellular Nav1.1 in this process. Using Nav1.1 silencing vectors, the authors show a slowing of cell doubling and progression through S phase, and further show that channel silencing reduces CDK2 protein levels and AKT phosphorylation. These conclusions, if correct, offer a novel functionality for sodium channels distinct from classical ion conduction. However, in the opinion of this reviewer, the strong and exclusive localization of Nav1.1 to the endoplasmic reticulum is not definitively established, nor is it conclusively established that the effect of silencing vectors is strictly due to reduction in Nav1.1 expression. Substantial revision, including new and repeat experiments, is required.
Major Points:
Exclusive localization of Nav1.1 to the ER: The Na current recordings in Fig. 1G,H purporting to show absence of sodium current in MSCs are flawed because there are no inhibitors of other voltage-gated currents. The large voltage-gated outward current in MSCs (presumably K+ current) could well have masked small inward sodium current. Secondly, the SCN1A immunofluorescence assay is not properly controlled. Many primary and secondary antibodies can give background, often appearing as an ER pattern. Immunofluorescence with SCN1A antibody should be controlled by parallel assay that includes SCN1A blocking peptide. Additionally or alternatively, is ER immunofluorescence signal reduced in si-SCN1A transfected cells?
Off-Target effects of silencing vectors: Any silencing system can have off-target effects, necessitating employment of multiple vectors. The authors employed two distinct si-SCN1a constructs and only one control construct. This does not appear adequate to rule out off-target effects. Employing two control constructs provides more confidence in specificity.
Other Points:
line 65: Grammar, should say "mutations in SCN1A have been reported ...."
Line 119: Why was SCN1A expression in MSCs investigated? Was there prior data pointing to some relevance of SCN1A in MSC function? Was SCN1A of interest because of RNAseq? As presented, the decision to explore SCN1A in MSCs seems random.
Line 133. The membrane potential assay does not provide any information relevant to this study, or if it does, this relevance has not been expounded.
The DiBAC4(3) experiment should be deleted.
Lines 446-458. The Na current recording method is faulty, since the bath and pipet solutions lack inhibitors of non-sodium voltage-gated currents specifically lacking Cd+2 to block VGCCs and TEA/4-AP to block VGKCs.. The MSCs have such strong outward (K+) current as to obscure potentially small sodium currents.
Fig2A. This quantification of SCN1A protein is questionable as shown because the ACTB reference signal is overexposed and not within range for densitometric scanning. In fact, the greater thickness of the overexposed ACTB signal in the siCTRL sample suggests that siSCN1A treatment may have less of a silencing effect than is reported.
Fig 2B: The effect of siSCN1A constructs on population doublings is much more prominent from days 0-3 than thereafter, particularly for construct 2, which showed no doubling until after day 3. Why might this be?

Reviewer 2

Advance summary and potential significance to field

This manuscript addresses a non-canonical role of a major neuronal voltage-gated sodium channel particularly in cell cycle progression. By western blot and immunofluorescence using specific

© 2024. Published by The Company of Biologists under the terms of the Creative Commons Attribution License (https://creativecommons.org/licenses/by/4.0/).

antibodies the authors show that $Na_V1.1$ is also expressed, although not functional, in mesenchymal stem cells. The question on whether it actually reaches the cell surface remains unanswered.

A ~70% siRNA depletion of endogenous $Na_V1.1$ is associated to reduced cell cycle progress, in particular, at the S phase. Cdk2 and Akt levels appear decreased as a result of the KD effect, with concomitant redistribution out of the nucleus of Cdk2. While some tendency may be obvious from the blots presented, the authors claim that the changes are linked neither to proteasomal nor to autophagy (lysosomal) activities. Instead, pharmacological inhibition of PI3K and activation of phospho-Akt led to implicate the Akt signaling pathway. How $Na_V1.1$ may be connected to the proposed mechanism, and ultimately responsible of its role in DNA synthesis, remains unsolved.

Comments for the author

While the whole hypothesis is worth considering, more supportive evidence is needed. In this regard, the following are suggestions to improve the manuscript:

1. Introduction – line 104: Why exploring $Na_V1.1$? Where is the reasoning? (See the Additional comments below.)
2. Results – line 128: Isn't $Na_V1.1$ actually being studied here as a potential instead of a "typical cell surface" protein?
3. Fig. 1C: can we exclude that NaV1.1 does not reach the cell surface and thus can't conduct Na^+ into the cytosol? – To ensure, one should do cell surface biotinylation.
4. Fig. 1B, C: do we have a negative control for anti-NaV1.1 blotting and IF staining on cells? – For instance, what about cells not expressing $Na_V1.1$? This is crucial to give certainty to the KD effect (Fig. 2A).
5. Results – line 154: the "Cell cycle analysis" with "(PI) staining" needs to be explained in more detail in the Methodology (line 483) to understand the graphs on cell number displayed in Fig. 2C – upper part.
6. Fig. 3C: evidence of Cdk2 reduction is provided by its decreased nuclear staining – could we have a subcellular nuclear fraction in which such decrease becomes also manifested thus biochemically? The same applies to the images in Fig. 5 and Fig. 6, with a single nucleus displayed in each panel.
7. While both bafilomycin A1 and the weak base ammonium chloride inhibit phagosome-lysosome fusion, and thus autophagy as the authors underline (line 186 and following), they both act by inhibiting lysosomal acidification. Therefore it is important to mention that the endolysosomal pathway is, together with the proteasome, the one whose implication is actually addressed in Fig. 4 and 5.
8. Fig. 6: while the effects attempted to analyze are pretty acute, incubation time of the LY reagent is 24 h. Yet, its inhibitory effects can be seen within minutes in cells (Vlahos et al. JBC 269: 5241 1994). One way to provide specificity here would be to show inhibition on Akt phosphorylation, by simply displaying parallel blots against Akt-P on Ser473 over total Akt in a time-course treatment and use the reagent at the shortest possible time.
9. Blots in Fig. 3, 4 and 6, while accompanied with bar graphs with quantitative data, will end up confusing the reader as to whether a real effect is observed or simply a statistically non-significant trend, therefore to be considered irrelevant. For instance, it is difficult to discriminate between Akt-P and total Akt decrease in KD cells, that is, whether the phosphorylated form is actually decreasing or simply because total Akt decreases. Analogously, in Fig. S4, isn't Phospho-Src decreasing substantially? Why? An approach that may help, while more time-consuming too, would be to immunoprecipitate (IP) each protein, i.e. Cdk2, Akt, etc., and assess more precisely, in the IP, the ratio of phosphorylated over total protein in each case.
10. Along these lines, the blots in Fig. 6C appear really hard to follow, especially because the samples arrangement does not parallel that in the bar graph; thus, contrary to what the graph shows, and judging from what we see in the blot it seems obvious that the Akt activator SC79 caused a decrease of Cdk2 levels in KD cells, but not a tendency to increase (line 234).
11. Discussion: in the first paragraph, and elsewhere, please edit the sentences for proper reading. For instance, "knockdown" affected (or slowed) "cell proliferation" instead of "damaged" (line 250). Revise also sentences in line 297 321, etc.

Additional minor comments:

© 2024. Published by The Company of Biologists under the terms of the Creative Commons Attribution License (https://creativecommons.org/licenses/by/4.0/).

1. Please use $_V$ instead of SCN1A when referred to the protein instead of the gene, for instance, in the Abstract – line 39. The same applies, in the Discussion, when talking about 1.5, 1.8 and the beta 3 subunit.
2. Introduction – line 66: “associated with” instead of “responsible for”
3. Introduction – line 81: the long sentence beginning as “Cyclin E-mediated CDK2 activation...” should be broken down into at least two sentences and rewritten for proper understanding.
4. Introduction – line 86: not clear, at this point, why there should be a link between Cdk2 and NaV channels; perhaps moving earlier the sentence on “Voltage-gated potassium channels…” (Line 92) would help.
5. Graphs are displayed as mean ± SEM, while mean ± SD is preferred to get a better idea of experimental variability around the average value or mean.
6. Supplementary Table 2: Please add the abbreviation for CREB; and, by looking at the text, it should be CANX for calnexin.
7. Please review References: in line 269, “Lee et al.”, as opposite to “Lee and Desiderio” (line 712); and, in line 277, “Zhang et al.” ‘a’ and ‘b’ to be specified in the Reference section (line 861).

First revision

Author response to reviewers' comments

RE: Point-by-Point Response to Reviewers' Comments

RE: Responses to Reviewer 1

Reviewer 1 Advance Summary and Potential Significance to Field
These conclusions, if correct, offer a novel functionality for voltage-gated sodium channels distinct from classical ion conduction. Substantiation of these conclusions shall require additional experiments.

Reviewer 1 Comments for the Author
This manuscript presents research examining the proliferation of mesenchymal stem cells (MSCs) and claims to define a requirement for intracellular Nav1.1 in this process. Using Nav1.1 silencing vectors, the authors show a slowing of cell doubling and progression through S phase, and further show that channel silencing reduces CDK2 protein levels and AKT phosphorylation. These conclusions, if correct, offer a novel functionality for sodium channels distinct from classical ion conduction. However, in the opinion of this reviewer, the strong and exclusive localization of Nav1.1 to the endoplasmic reticulum is not definitively established, nor is it conclusively established that the effect of silencing vectors is strictly due to reduction in Nav1.1 expression. Substantial revision, including new and repeat experiments, is required.

Response: We thank Reviewer 1 for your deep concerns and suggestive comments on our manuscript. We have added new and repeated data according to your suggestions and responded to your concerns/questions in this revised manuscript. Please find our point- by-point response, as well.

Reviewer 1 Major Points
Point 1. Exclusive localization of Nav1.1 to the ER
The Na current recordings in Fig. 1G,H purporting to show absence of sodium current in MSCs are flawed because there are no inhibitors of other voltage-gated currents. The large voltage-gated outward current in MSCs (presumably K+ current) could well have masked small inward sodium current.

Response: We appreciate Reviewer 1 to your deep concerns about our electrophysiological recording in mesenchymal stem cells (MSCs). In the present recording, we used a cerium (Cs) - based solution (140 mM CsCl, 10 mM EGTA,1.5 mM $MgCl_2$, 10 mM glucose, and 10 mM HEPES, pH

© 2024. Published by The Company of Biologists under the terms of the Creative Commons Attribution License (https://creativecommons.org/licenses/by/4.0/).

7.2) as an intracellular (pipet) solution.
The Cs-based solution can block almost all K^+ current in a voltage-dependent manner [Hagiwara et al., 1976; Clay and Shlesinger, 1983, 1984]. Therefore, the outward currents observed in the submitted manuscript were presumably Cl^- currents or pure leak currents. To prevent misinterpretation of the outward currents, we replaced the figure with one where the leak current is minimized.

We additionally investigated another recording in the presence of a sodium channel blocker, tetrodotoxin (TTX). We clearly demonstrate that the induced current is a voltage- gated Na^+ current, which is blocked by TTX.

In this revised manuscript, we have added a new section, "Recording buffers", in the Materials and Methods section. We have also represented all the recording data as an independent figure with new recording data, Fig. 2 and Fig. S2, and explained more detail in the Results section in this revised manuscript.

Point 2. SCN1A immunofluorescence assay
Secondly, the SCN1A immunofluorescence assay is not properly controlled. Many primary and secondary antibodies can give background, often appearing as an ER pattern. Immunofluorescence with SCN1A antibody should be controlled by parallel assay that includes SCN1A blocking peptide. Additionally or alternatively, is ER immunofluorescence signal reduced in si-SCN1A transfected cells?

Response: Thank you for the critical suggestions for the negative control tests and your concern about the ER pattern in SCN1A siRNA-transferred cells by immunofluorescence (IF). The primary monoclonal antibody of SCN1A (NaV1.1) for immunofluorescence in the submitted manuscript recognized C-terminal amino acids (1989-2009) of rat NaV1.1. However, it was very difficult for us to obtain the corresponding long 80 amino acid peptide before the deadline for this revision (90 days). Thus, in this revision, to fulfill the Reviewer's opinion, we employed another antibody, which was already used in the western blot assay in the submitted manuscript, and obtained the neutralized antibody, which was reacted with the commercially available blocking peptide corresponding to the epitope of the antibody, amino acid residues 465-481 of NaV1.1 (Accession NP_001159435). We then showed the similar localization of NaV1.1 in MSCs and verified the immunological specificity of the primary antibody. We have explained in the Results and Materials and Methods sections and added Fig. 1B and 1C in this revised manuscript.

Point 3. Off-Target effects of silencing vectors

Any silencing system can have off-target effects, necessitating employment of multiple vectors. The authors employed two distinct si-SCN1a constructs and only one control construct. This does not appear adequate to rule out off-target effects. Employing two control constructs provides more confidence in specificity.

Response: We thank Reviewer 1's suggestion for our gene silencing experiment. According to the suggestion, we employed another control siRNA, siCTRL#, in this revision. As shown in the attached Point-by-Point Fig. 1-3, we could rule out the off- target effects, verifying the specificity of our gene-silencing results in the present siRNA experiment. However, we could not add these new results and figures, because of limitation and space for total words and figure numbers.

Reviewer 1 Minor Points

Point 1. line 65: Grammar, should say "mutations in SCN1A have been reported "

Response: Thank you for your attention to the sentence on line 65. We have corrected the sentence according to the Reviewer's suggestion in this revised manuscript.

Point 2. Line 119: Why was SCN1A expression in MSCs investigated? Was there prior data pointing to some relevance of SCN1A in MSC function? Was SCN1A of interest because of RNAseq? As presented, the decision to explore SCN1A in MSCs seems random.

© 2024. Published by The Company of Biologists under the terms of the Creative Commons Attribution License (https://creativecommons.org/licenses/by/4.0/).

Response: As Reviewer 1 pointed out, the submitted manuscript did not contain an explanation of the reason why we explore SCN1A (NaV1.1) in mesenchymal stem cells (MSCs). Because the divided progenies of MSCs, which are defined as self-renew and multipotency, give rise to tissue maintenance and repair throughout human life, it is considered that the regulation of asymmetric and symmetric mitosis in MSCs is crucial for the life-long maintenance of humans. A recent study reported electrical stimulation regulates osteogenic differentiation of MSCs through voltage-gated sodium (NaV) and potassium (KV) channels [Zhang et al., 2015]. KVs participate in the cell cycle progression and DNA synthesis of proliferative MSCs [Zhang et al., 2014]. However, the involvement of NaV1.1 in the proliferation, cell cycle progression, and DNA synthesis of MSCs remains unclear, even though mTORC1-induced NaV1.1 participates in the regulation of senescence via Ca^{2+}influx-controlled NFAT/ATF3/p53 signaling in MSC-committed preosteoblasts [Chen et al., 2022]. Therefore, in this study, we focused on the role of NaV1.1 in regulating the cell cycle of normal human MSCs. However, our submitted manuscript did not contain a sufficient explanation for Reviewer 1's pointed matter. We have added the reason on in the Introduction section of this revised manuscript as the following sentence: "However, the involvement of Nav1.1 in the proliferation, cell cycle progression, and DNA synthesis of MSCs remains unclear (Chen et al., 2022).".

Point 3. Line 133. The membrane potential assay does not provide any information relevant to this study, or if it does, this relevance has not been expounded. The DiBAC4(3) experiment should be deleted.

Response: We thank you for your suggestion. We have deleted the points in this revised manuscript.

Point 4. Lines 446-458. The Na current recording method is faulty, since the bath and pipet solutions lack inhibitors of non-sodium voltage-gated currents, specifically lacking Cd+2 to block VGCCs and TEA/4-AP to block VGKCs. The MSCs have such strong outward (K+) current as to obscure potentially small sodium currents.

Response: We thank Reviewer 1 for your deep concerns about our electrophysiological assay in the submitted manuscript, as pointed out above. Please refer to the response to Point 1.

Point 5. Fig2A. This quantification of SCN1A protein is questionable as shown because the ACTB reference signal is overexposed and not within range for densitometric scanning. In fact, the greater thickness of the overexposed ACTB signal in the siCTRL sample suggests that siSCN1A treatment may have less of a silencing effect than is reported.

Response: Thank you for your deep concern about the actin beta signals by western blot analysis in siRNA experiment of the submitted manuscript. We have corrected the results and added on Fig. 3A in this revised manuscript.

Point 6. Fig 2B: The effect of siSCN1A constructs on population doublings is much more prominent from days 0-3 than thereafter, particularly for construct 2, which showed no doubling until after day 3. Why might this be?

Response: Response: We thank you for your great concern to the results of our population doubling test. We think that the reason for this concern depends on the different cell colony-derived origins of MSCs and different transfection efficacy of siRNA as below: In this experiment, we transfected siRNAs into MSCs every passage. Since MSCs are a heterogeneous colony-derived cell population (multi-colony-derived population) [Miura et al., 2003, Li et al., 2023], each colony-derived cell/cell population may show a different transfection efficacy after cell passage, resulting in causing a different cell proliferation kinetics compared to the parent cell population. It is also hard to control the transfection efficacy constantly in multi-colony-derived cell population at each passage, even though our siRNAs effectively suppressed the proliferation of MSCs.

References in the responses to Reviewer 1

1. Hagiwara S, Miyazaki S, Rosenthal NP. Potassium current and the effect of cesium on this current during anomalous rectification of the egg cell membrane of a starfish. J Gen Physiol.

© 2024. Published by The Company of Biologists under the terms of the Creative Commons Attribution License (https://creativecommons.org/licenses/by/4.0/).

1976;67:621–638. doi: 10.1085/jgp.67.6.621.
2. Clay JR, Shlesinger MF. Effects of external cesium and rubidium on outward potassium currents in squid axons. Biophys J. 1983;42:43-53. doi: 10.1016/S0006- 3495(83)84367-7.
3. Clay JR, Shlesinger MF. Analysis of the effects of cesium ions on potassium channel currents in biological membranes. J Theor Biol. 1984;107:189–201. doi: 10.1016/s0022-5193(84)80021-1.
4. Plumereau Q, Ebdalla A, Poulin H, Appendino JP, Scantlebury MH, Au PYB, Chahine M. De novo Y1460C missense variant in NaV1.1 impedes the pore region and results in epileptic encephalopathy. Sci Rep. 2022;12:17182. doi: 10.1038/s41598-022-22208-x.
5. Fu W, Vasylyev D, Bi Y, Zhang M, Sun G, Khleborodova A, Huang G, Zhao L, Zhou R, Li Y, Liu S, Cai X, He W, Cui M, Zhao X, Hettinghouse A, Good J, Kim E, Strauss E, Leucht P, Schwarzkopf R, Guo EX, Samuels J, Hu W, Attur M, Waxman SG, Liu CJ. Nav1.7 as a chondrocyte regulator and therapeutic target for osteoarthritis. Nature. 2024;625:557–565. doi: 10.1038/s41586-023-06888-7.
6. Zhang J, Li M, Kang ET, Neoh KG. Electrical stimulation of adipose-derived mesenchymal stem cells in conductive scaffolds and the roles of voltage-gated ion channels. Acta Biomater. 2016 Mar 1;32:46-56. doi: 10.1016/j.actbio.2015.12.024.
7. Zhang Y, Yue J, Che H, Sun H, Tse H, Li G. BKCa and hEAG1 channels regulate cell proliferation and differentiation in human bone marrow-derived mesenchymal stem cells. J Cell Physiol. 2014;229:202–212. doi: 10.1002/jcp.24435.
8. Chen A, Jin J, Cheng S, Liu Z, Yang C, Chen Q, Liang W, Li K, Kang D, Ouyang Z, Yao C, Bai X, Li Q, Jin D, Huang B. mTORC1 induces plasma membrane depolarization and promotes preosteoblast senescence by regulating the sodium channel Scn1a. Bone Res. 2022;10:25. doi: 10.1038/s41413-022-00204-1
9. Miura M, Gronthos S, Zhao M, Lu B, Fisher WL, Robey GP, Shi S. SHED: stem cells from human exfoliated deciduous teeth. Proc Natl Acad Sci U. S. A., 2003; 100; 5807-5812. doi: 10.1073/pnas.0937635100
10. Li J, Wu Z, Zhao L, Liu Y, Su Y, Gong X, Liu F, Zhang L. The heterogeneity of mesenchymal stem cells: an important issue to be addressed in cell therapy. Stem Cell Res Ther. 2023;14, 381. doi: 10.1186/s13287-023-03587-y

RE: Responses to Reviewer 2

Reviewer 2 Advance Summary and Potential Significance to Field
This manuscript addresses a non-canonical role of a major neuronal voltage-gated sodium channel, particularly in cell cycle progression. By western blot and immunofluorescence using specific antibodies, the authors show that NaV1.1 is also expressed, although not functional, in mesenchymal stem cells. The question on whether it actually reaches the cell surface remains unanswered. A ~70% siRNA depletion of endogenous NaV1.1 is associated to reduced cell cycle progress, in particular, at the S phase. Cdk2 and Akt levels appear decreased as a result of the KD effect, with concomitant redistribution out of the nucleus of Cdk2. While some tendency may be obvious from the blots presented, the authors claim that the changes are linked neither to proteasomal nor to autophagy (lysosomal) activities. Instead, pharmacological inhibition of PI3K and activation of phospho-Akt led to implicate the Akt signaling pathway. How NaV1.1 may be connected to the proposed mechanism, and ultimately responsible of its role in DNA synthesis, remains unsolved.

Reviewer 2 Comments for the Author
While the whole hypothesis is worth considering, more supportive evidence is needed. In this regard, the following are suggestions to improve the manuscript:

Response: We thank you for your suggestive comments on our manuscript. We have added new and repeated data according to your suggestions and responded to your concerns in this revised manuscript. Please find our point-by-point response, as well.

Point 1. Introduction - line 104: Why exploring NaV1.1? Where is the reasoning? (See the Additional comments below.)

© 2024. Published by The Company of Biologists under the terms of the Creative Commons Attribution License (https://creativecommons.org/licenses/by/4.0/).

Response: As Reviewer 2 pointed out, the submitted manuscript did not contain an explanation of the reason why we explore NaV1.1 in mesenchymal stem cells (MSCs). Because the divided progenies of MSCs, which are defined as self-renew and multipotency, give rise to tissue maintenance and repair throughout human life, it is considered that the regulation of asymmetric and symmetric mitosis in MSCs is crucial for the life-long maintenance of humans. A recent study showed electrical stimulation regulates osteogenic differentiation of MSCs through voltage-gated sodium (NaV) and potassium (Kv) channels [Zhang et al., 2015]. Potassium ion channels participate in the cell cycle progression and DNA synthesis of proliferative MSCs [Zhang et al., 2014]. However, the involvement of NaV1.1 in the proliferation, cell cycle progression, and DNA synthesis of MSCs remains unclear, even though mTORC1-induced NaV1.1 participates in the regulation of senescence via Ca^{2+}influx-controlled NFAT/ATF3/p53 signaling in MSC-committed preosteoblasts [Chen et al., 2022]. Therefore, in this study, we focused on the role of NaV1.1 in regulating the cell cycle of normal human MSCs. However, our submitted manuscript did not contain a sufficient explanation for Reviewer 2's pointed matter. We have added the reason on in the Introduction section of this revised manuscript as the following sentence: "However, the involvement of Nav1.1 in the proliferation, cell cycle progression, and DNA synthesis of MSCs remains unclear (Chen et al., 2022).".

Point 2. Results - line 128: Isn't NaV1.1 actually being studied here as a potential instead of a "typical cell surface" protein?

Response: Thank you for your helpful suggestion. The current explanation was insufficient. We have corrected the part in the Results section of this revised manuscript as the following sentences: "CD90 is known as a potential cell surface marker of MSCs and neuroblastomas (Ferreira-Facio et al., 2013; Sonoda et al., 2021).".

Point 3. Fig. 1C: can we exclude that NaV1.1 does not reach the cell surface and thus can't conduct Na^+ into the cytosol? - To ensure, one should do cell surface biotinylation.

Response: Thank you for your critical concern and helpful suggestion about the surface localization of NaV1.1. We agree with your idea to evaluate the cell surface expression of NaV1.1. Although we already showed the related data by the present protease K protection assay on Fig 1D, in this revised manuscript, we additionally employed the other biochemical method, a density gradient centrifugation, instead of a cell surface biotinylation assay, to analyze the expression of NaV1.1 in plasma membrane and endoplasmic reticulum fractions of MSCs and SH-SY5Y cells by Western blot analysis. The new biochemical results clearly showed the localization in cell surface and endoplasmic reticulum of NaV1.1. We have explained the results and method and have added Fig1. E in this revised manuscript.

Point 4. Fig. 1B, C: do we have a negative control for anti-NaV1.1 blotting and IF staining on cells? - For instance, what about cells not expressing NaV1.1? This is crucial to give certainty to the KD effect (Fig. 2A).

Response: Thank you for your critical question and helpful suggestion. In this revised manuscript, we used the blocking peptide specific to anti-SCN1A antibody for negative control tests in western blot analysis and immunofluorescence. We have explained the results and method and have added Fig. 1B and 1C in this revised manuscript.

Point 5. Results - line 154: the "Cell cycle analysis" with "(PI) staining" needs to be explained in more detail in the Methodology (line 483) to understand the graphs on cell number displayed in Fig. 2C - upper part.

Response: We appreciate Reviewer 2 to the point. Our explanation about propidium iodide (PI) staining was insufficient in the submitted manuscript. The PI dye can bind to double-strand DNA. Therefore, the PI staining allows quantifying cellular DNA content (2n, 2n-4n, and 4n) in cells. Each cell cycle phase of G0/G1, S, and G2/M phase contains 2n, 2n-4n, and 4n of DNA contents in cells, respectively. Thus, the histograms of flow cytometry represent two independent peaks; the G2/M phase population shows double fluorescence intensity compared to the G0/G1 phase

© 2024. Published by The Company of Biologists under the terms of the Creative Commons Attribution License (https://creativecommons.org/licenses/by/4.0/).

population. The intermediate population between the peaks represents the cells in the S phase. We have added detailed explanations about PI staining in the Results section of this revised manuscript as the following sentence: "Cell cycle analysis using FCM and propidium iodide (PI) staining revealed that siSCN1A-MSCs had an increased S-phase (2n-4n DNA content) population and a decreased G2/M-phase (4n DNA content) population, but a similar G0/G1 phase (2n DNA content) population compared to siCTRL-MSCs (**Fig. 3D**).". We have also added the detailed labeling on Fig. 3D in this revised manuscript.

Point 6. Fig. 3C: evidence of Cdk2 reduction is provided by its decreased nuclear staining - could we have a subcellular nuclear fraction in which such decrease becomes also manifested thus biochemically? The same applies to the images in Fig. 5 and Fig. 6, with a single nucleus displayed in each panel.

Response: We thank Reviewer 2 for the critical suggestion. According to the suggestion, we have examined the nuclear expression of CDK2 by Western blot analysis using nuclear fraction and added the new results and Fig. 6B, 6D, 6F, 7C, and 8C in this revised manuscript.

Point 7. While both bafilomycin A1 and the weak base ammonium chloride inhibit phagosome-lysosome fusion, and thus autophagy, as the authors underline (line 186 and following), they both act by inhibiting lysosomal acidification. Therefore it is important to mention that the endolysosomal pathway is, together with the proteasome, the one whose implication is actually addressed in Fig. 4 and 5.

Response: We thank Reviewer 2 for your helpful suggestion to mention the implication of the endolysosomal system together with proteasome and autophagy in this study. We have corrected the parts throughout this revised manuscript.

Point 8. Fig. 6: while the effects attempted to analyze are pretty acute, incubation time of the LY reagent is 24 h. Yet, its inhibitory effects can be seen within minutes in cells (Vlahos et al. JBC 269: 5241 1994). One way to provide specificity here would be to show inhibition on Akt phosphorylation, by simply displaying parallel blots against Akt-P on Ser473 over total Akt in a time-course treatment and use the reagent at the shortest possible time.

Response: We appreciate Reviewer 2's deep concern and helpful suggestion regarding the phosphorylated AKT (pAKT) expression in LY2949002 (LY)-treated MSCs. According to the suggestion, we employed a time-course experiment 0, 5, 10, and 20 minutes after LY treatment. We then analyzed the expression of nuclear CDK2 by immunofluorescence and Western blot analysis using nuclear fraction, as well as total CDK2 by Western blot analysis between LY- and MOCK-treated MSCs. However, there was no difference in nuclear and total CDK2 between LY- and MOCK-treated MSCs after LY treatment. We have added the results and Fig. 7 of this revised manuscript. We have also discussed these results in the Discussion part of this revised manuscript.

Point 9. Blots in Fig. 3, 4 and 6, while accompanied with bar graphs with quantitative data, will end up confusing the reader as to whether a real effect is observed or simply a statistically non-significant trend, therefore to be considered irrelevant. For instance, it is difficult to discriminate between Akt-P and total Akt decrease in KD cells, that is, whether the phosphorylated form is actually decreasing or simply because total Akt decreases. Analogously, in Fig. S4, isn't Phospho-Src decreasing substantially? Why? An approach that may help, while more time-consuming too, would be to immunoprecipitate (IP) each protein, i.e. Cdk2, Akt, etc., and assess more precisely, in the IP, the ratio of phosphorylated over total protein in each case.

Response: We thank reviewer 2 for the critical points in Fig. 3, 4, 6, and 4S. As you pointed out, the explanations of Fig. 3, 4, 6, and 4S bothered you and the other readers in the submitted manuscript. So, we have addressed the results more statistically in this revised manuscript. Phospho-SRC tended to decrease in siSCN1A-MSC, however, it was not statistically significant. It was therefore not studied in the present study.

To discriminate which reduction in total and phosphorylated pAKT plays an important role in SCN1A-silencing MSCs, as Reviewer 2 suggested, we have employed more time-consuming tests for LY treatment in short periods (0, 5, 10, and 20 min) in this revised manuscript. We have also

© 2024. Published by The Company of Biologists under the terms of the Creative Commons Attribution License (https://creativecommons.org/licenses/by/4.0/).

analyzed nuclear CDK2 expression under LY and SC79 treatments. As we showed in Fig. 8B and 8C, we found the activity of AKT did not participate in the CDK2 localization.

Point 10. Along these lines, the blots in Fig. 6C appear really hard to follow, especially because the samples arrangement does not parallel that in the bar graph; thus, contrary to what the graph shows, and judging from what we see in the blot, it seems obvious that the Akt activator SC79 caused a decrease of Cdk2 levels in KD cells, but not a tendency to increase (line 234).

Response: We appreciate Reviewer 2 to his/her points. We have aligned the order of the western blot data with the order of the graph in Fig. 8A (Fig. 6C in submitted manuscript). We also reexamined the effect of SC79 on CDK2 and confirmed that SC79 did not affect the CDK2 levels in siSCN1A-MSCs.

Point 11. Discussion: in the first paragraph, and elsewhere, please edit the sentences for proper reading. For instance, "knockdown" affected (or slowed) "cell proliferation" instead of "damaged" (line 250). Revise also sentences in line 297, 321, etc.

Response: Thank you for your attention to the points. We have edited the points in this revised manuscript.

Additional minor comments

Point 1. Please use V instead of SCN1A when referred to the protein instead of the gene, for instance, in the Abstract - line 39. The same applies, in the Discussion, when talking about 1.5, 1.8 and the beta 3 subunit.

Response: We appreciate Reviewer 2's suggestion to use NaV1.1 instead of SCN1A. We have corrected all the points when referring to the protein instead of the gene in all contents of this revised manuscript.

Point 2. Introduction - line 66: "associated with" instead of "responsible for"

Response: Thank you for your attention to the point. We have corrected the point in this revised manuscript.

Point 3. Introduction - line 81: the long sentence beginning as "Cyclin E-mediated CDK2 activation..." should be broken down into at least two sentences and rewritten for proper understanding.

Response: We appreciate Reviewer 2's attention and helpful suggestion for the long sentence from line 81. In this revised manuscript, we have rewritten the sentence to the following sentences: "Cyclin E-mediated CDK2 activation can lead to the phosphorylation of the substrate, retinoblastoma protein (RB), which is associated with E2F, to suppress transcriptional activity. The phosphorylation of RB relieve E2F to allow the transcriptional activity of genes for the promotion of essential cell cycle processes, such as DNA replication and mitosis (Seo et al., 2006; Pennycook et al., 2020).".

Point 4. Introduction - line 86: not clear, at this point, why there should be a link between Cdk2 and NaV channels; perhaps, moving earlier the sentence on "Voltage- gated potassium channels…" (Line 92) would help.

Response: Thank you for your helpful suggestion for the sentence on line 86. We have corrected in this revised manuscript according to the Reviewer 2 's suggestion.

Point 5. Graphs are displayed as mean ± SEM, while mean ± SD is preferred to get a better idea of experimental variability around the average value or mean.

© 2024. Published by The Company of Biologists under the terms of the Creative Commons Attribution License (https://creativecommons.org/licenses/by/4.0/).

Response: We appreciate Reviewer 2’s suggestion to the variability. All the graphs of this revised manuscript have been corrected to display as mean ± SD.

Point 6. Supplementary Table 2: Please add the abbreviation for CREB; and, by looking at the text, it should be CANX for calnexin.

Response: Thank you for your attention to the Supplementary Table 2. We have added and corrected the abbreviations in the Supplementary Table 2 of this revised manuscript.

Point 7. Please review References: in line 269, “Lee et al.”, as opposite to “Lee and Desiderio” (line 712); and, in line 277, “Zhang et al.” ‘a’ and ‘b’ to be specified in the Reference section (line 861).

Response: Thank you for your attention to the References. We have corrected the points in this revised manuscript.

References in the responses to Reviewer 2

1. Zhang J, Li M, Kang ET, Neoh KG. Electrical stimulation of adipose-derived mesenchymal stem cells in conductive scaffolds and the roles of voltage-gated ion channels. Acta Biomater. 2016 Mar 1;32:46-56. doi: 10.1016/j.actbio.2015.12.024.
2. Zhang Y, Yue J, Che H, Sun H, Tse H, Li G. BKCa and hEAG1 channels regulate cell proliferation and differentiation in human bone marrow-derived mesenchymal stem cells. J Cell Physiol. 2014;229:202–212. doi: 10.1002/jcp.24435.
3. Chen A, Jin J, Cheng S, Liu Z, Yang C, Chen Q, Liang W, Li K, Kang D, Ouyang Z, Yao C, Bai X, Li Q, Jin D, Huang B. mTORC1 induces plasma membrane depolarization and promotes preosteoblast senescence by regulating the sodium channel Scn1a. Bone Res. 2022;10:25. doi: 10.1038/s41413-022-00204-1

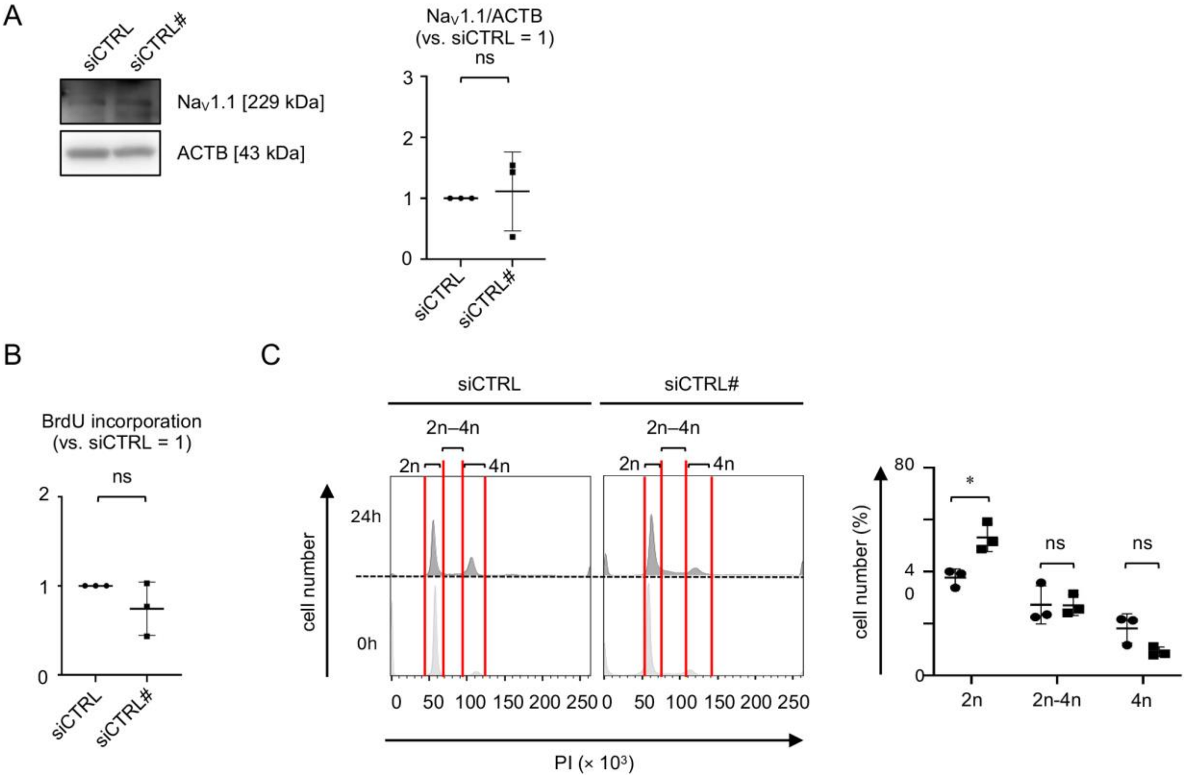

Point-by-Point Figure 1. Cell cycle analysis in control small interferent RNA-treated MSCs. MSCs were transfected with small interferent RNA (siRNA) of control siRNA, siCTRL and siCTRL#. (A) Representative WB images of the expression of sodium voltage-gated channel alpha subunit 1 protein (Na$_V$1.1) in MSCs. The graph shows the ratio of Na$_V$1.1 expression in MSCs based on WB analysis. The results are presented as the ratio of Na$_V$1.1 to ACTB expression in siCTRL-treated

© 2024. Published by The Company of Biologists under the terms of the Creative Commons Attribution License (https://creativecommons.org/licenses/by/4.0/).

MSCs (vs. siCTRL = 1). (B) Bromodeoxyuridine (BrdU) incorporation into MSCs. The results are presented as the ratios of the BrdU incorporation rate in siCTRL-MSCs (vs. siCTRL = 1). (C) MSCs were maintained for 24 h under serum- depleted conditions and analyzed 0 (0 h) and 24 (24 h) h after serum stimulation using flow cytometry (FCM) with propidium iodide (PI) staining. Representative histograms of the DNA contents (2n, 2n-4n, and 4n) in MSCs. The graph shows the rate (%) of MSCs in the DNA contents based on FCM with PI staining. (A–C) siCTRL, siCTRL-treated group; siCTRL#, siCTRL#-treated group. Data are presented as mean ± SD. *n* = 3/group. Significance was determined using independent two-tailed Student's *t*-test. ns, no significance.

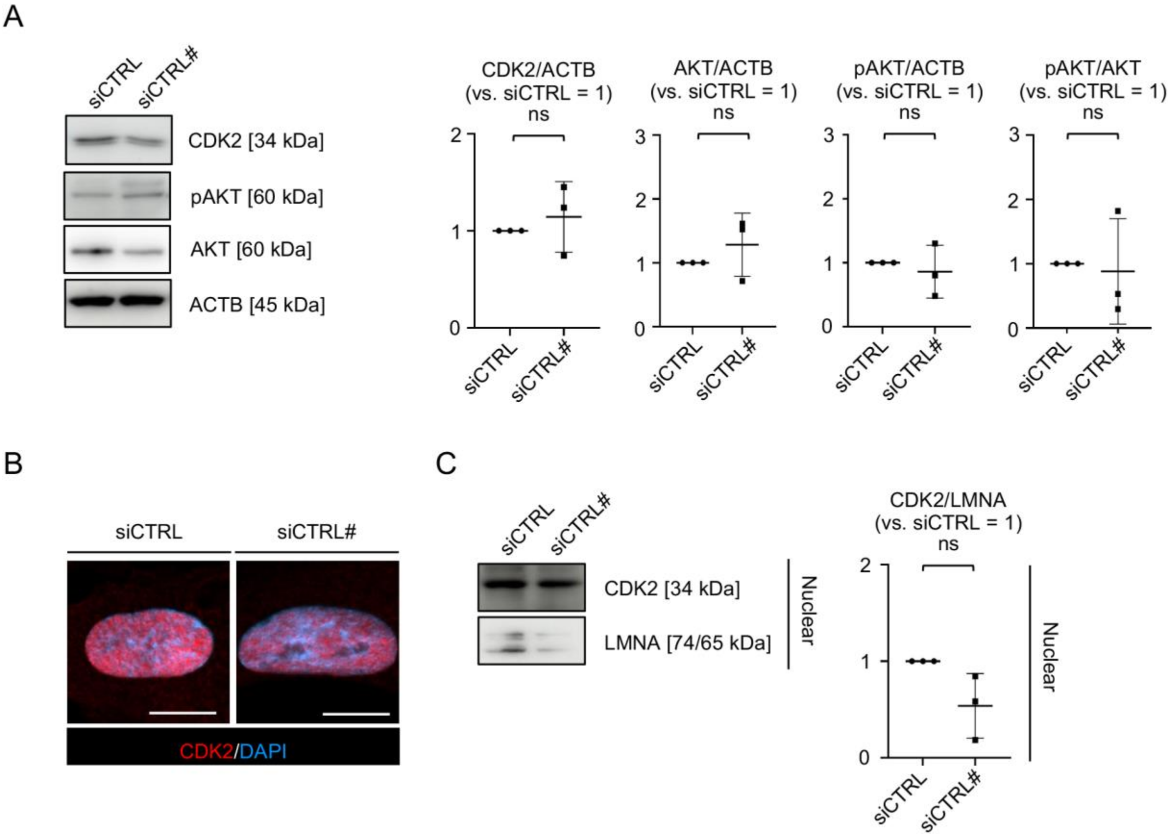

Point-by-Point Figure 2. The expression of cyclin-dependent kinase 2 and AKT in control siRNA-treated MSCs. MSCs were transfected with siCTRL and siCTRL#. (A) Representative WB images of the expression of cyclin-dependent kinase 2 (CDK2), AKT and phosphorylated AKT (pAKT) in MSCs. The graphs show the ratios of CDK2, AKT, and pAKT in MSCs based on WB analysis. The results are presented as the ratios of the expression of CDK2, AKT, and pAKT to that of Actin beta (ACTB) in siCTRL-MSCs (vs. siCTRL = 1). (B) Representative immunofluorescence images of the localization of CDK2 in MSCs based on immunofluorescence analysis. The nuclei are stained with DAPI. Scale bars, 10 □m. (C) Representative WB images of the expression of nuclear CDK2 in MSCs. The graphs show the ratios of CDK2 in MSCs based on WB analysis. Lamin A/C (LMNA) was used as the internal control. The results are presented as the ratios of the expression of CDK2 to that of LMNA in siCTRL-MSCs (vs. siCTRL = 1). (A–C) siCTRL, siCTRL-treated group; siCTRL#, siCTRL#-treated group. (A, C) Data are presented as mean ± SD. *n* = 3/group. Significance was determined using independent two-tailed Student's *t*-test. ns, no significance.

© 2024. Published by The Company of Biologists under the terms of the Creative Commons Attribution License (https://creativecommons.org/licenses/by/4.0/).

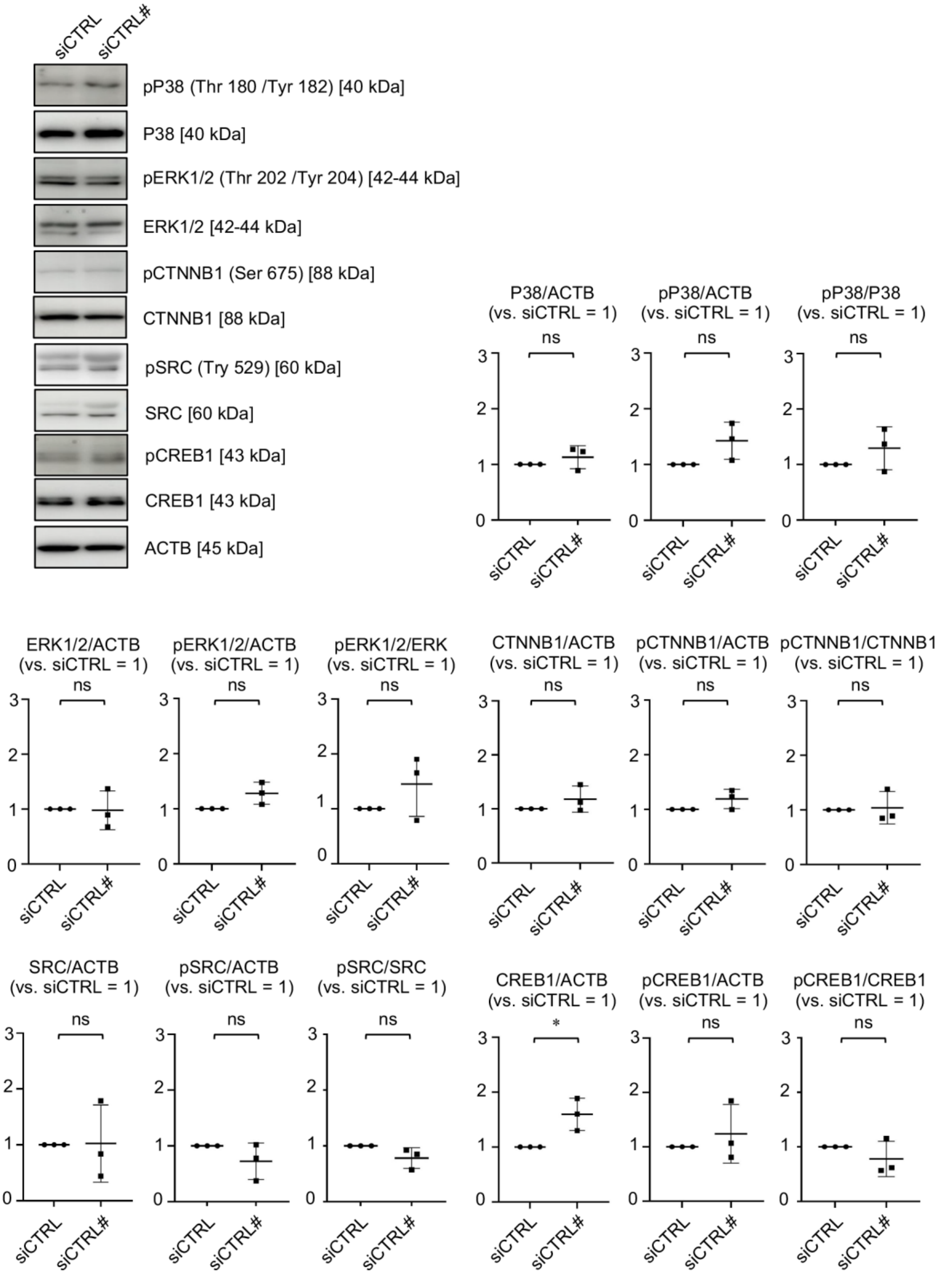

Point-by-Point Figure 3. Expression of intercellular signal molecules in control siRNA-treated MSCs. MSCs were transfected with siCTRL and siCTRL#. Representative WB images of the expression of P38, phosphorylated P38 (pP38), extracellular signal-regulated kinase (ERK), phosphorylated ERK (pERK), catenin beta 1 (CTNNB1), and phosphorylated CTNNB1 (p CTNNB1), SRC, phosphorylated SRC (pSRC), cyclic AMP-responsive element-binding protein 1 (CREB), and phosphorylated CREB (pCREB) in MSCs. The graphs show the ratios of the expression of P38, pP38, ERK, pERK, CTNNB1, pCTNNB, SRC, pSRC, CREB1, and pCREB1 in MSCs based on WB. Actin beta (ACTB) was used as the internal control. The results are shown as the ratios of the expression of P38, pP38, ERK, pERK, CTNNB1, pCTNNB, SRC, pSRC, CREB1, and pCREB1 to the expression of ACTB in siCTRL-

© 2024. Published by The Company of Biologists under the terms of the Creative Commons Attribution License (https://creativecommons.org/licenses/by/4.0/).

treated MSCs (vs. siCTRL = 1). Data are presented as mean ± SD. *n* = 3/group. Significance was determined using independent two-tailed Student's *t*-test. ns, no significance. siCTRL, siCTRL-treated group; siCTRL#, siCTRL#-treated group.

Second decision letter

MS ID#: JOCES/2023/261732

MS TITLE: $Na_V1.1$ contributes to the cell cycle of human mesenchymal stem cells by regulating AKT and CDK2

AUTHORS: Mohammed Fouad Zakaria, Hiroki Kato, Soichiro Sonoda, Kenichi Kato, Norihisa Uehara, Yukari Kyumoto-Nakamura, Mohammed Majd Sharifa, Liting Yu, Lisha Dai, Haruyoshi Yamaza, Shunichi Kajioka, Fusanori Nishimura, and Takayoshi Yamaza

We have now reached a decision on the above manuscript.

To see the reviewers' reports and a copy of this decision letter, please go to: https://submit-jcs.biologists.org and click on the 'Manuscripts with Decisions' queue in the Author Area. (Corresponding author only has access to reviews.)

As you will see, the reviewers still raise a number of criticisms that prevent me from accepting the paper at this stage. They suggest, however, that a revised version might prove acceptable, if you can address their concerns. If you think that you can deal satisfactorily with the criticisms on revision, I would be pleased to see a revised manuscript. We would then return it to the reviewers.

Please ensure that you clearly highlight all changes made in the revised manuscript. Please avoid using 'Tracked changes' in Word files as these are lost in PDF conversion.

I should be grateful if you would also provide a point-by-point response detailing how you have dealt with the points raised by the reviewers in the 'Response to Reviewers' box. Please attend to all of the reviewers' comments. If you do not agree with any of their criticisms or suggestions please explain clearly why this is so.

Reviewer 1

Advance summary and potential significance to field

There are ample studies documenting the role of voltage-gated sodium channels (VGSCs) in tumor progression. This manuscript makes two further advances:
1) It defines a signal transduction pathway underlying VGSC control of cell cycling.
2) It posits that a VGSC is acting in a vesicular compartment independent of its sodium conducting properties However, this second crucial conclusion is not adequately supported by the data and further experiments are warranted, as delineated in the suggestions to authors.

Comments for the author

Significant revision in the form of confirmatory experiments is necessary to make this manuscript acceptable for publication:

A central thesis of this revised manuscript from Zakaria et al. is that Nav1.1's effects on mesenchymal stem cell proliferation are via a sodium current-independent pathway. Their supporting evidence for this claim is that plasma membrane voltage-clamped sodium currents could not be detected in the MSC cells.
However, small sodium currents may be masked by outward leak and voltage-gated K currents, which are evident in the current traces in Figure 2. The experiments on SH-SY5Y cells, where

© 2024. Published by The Company of Biologists under the terms of the Creative Commons Attribution License (https://creativecommons.org/licenses/by/4.0/).

sodium current is readily apparent and sensitive to TTX does not resolve the problem with the MSC cells.

1) Recordings should be repeated: a) in presence of tetraethylammonium choride and 4-aminopurine to suppress voltage-gated K+ currents, and b) by applying P/N leak subtraction during recording (available in Clampex) to eliminate leak and capacitive currents.
2) Additionally, if the role of Nav1.1 on cycling is independent of sodium currents, the cycling effects should be resistant to TTX. Alternatively, if sodium currents contribute to cycling, the role of Nav1.1 should be TTX-sensitive. This is a simple experiment that the authors should undertake.

Reviewer 2

Advance summary and potential significance to field

In this revised version, the authors have addressed all the issues requested by the reviewers, involving the addition of some data, subsequently making several changes to the manuscript, which can be seen as considerably improved. The polished presentation now offered in both text and data is greatly appreciated.

Comments for the author

At this point, I can only point out some comments that – in my opinion – should be taken into account:

1. In general, the text should be checked for proper clarity – take a look, for instance, at the Abstract. As an example when one talks about gene expression, referring to the process of converting genetic information into either RNA or protein, it sounds strange to say that “cells express” the SCN1A gene. Then, toward the end, “AKT inhibitor LY294002 did not... On the contrary, AKT activator SC79 did not…” If both “did not”, one can’t say “On the contrary”, maybe “Likewise”.

Therefore, authors must ensure proper usage of conjunctive adverbs – throughout the whole text – to avoid confusion and ensure smooth reading.

2. The non-canonical role of NaV1.1 described in this work provides added evidence for the involvement of voltage-gated ion channels in other functions. However, the subcellular location of these channels is an aspect that should not be overlooked since it will certainly influence the conclusions reached. The data added by the authors in this regard are only somewhat convincing, although I understand it is not easy to go further. Virtually all NaV1.1 is intracellular in the cell model studied (just look at Fig. 1D and 1E) and, for the most part, that’s why we don’t see current.

3. Finally, undoubtedly, the knockdown effect achieved is not dramatic (Fig. 3A). Therefore, subsequent data can be expected to lack robustness. Compare, for instance, the nuclear localization of Cdk2 still observed in siRNA-treated cells (Fig. 6E) contrasting to a visible reduction shown earlier in the manuscript (Fig. 4C). Nonetheless, overall, it seems quite convincing that the Akt/Cdk2 pathway is downstream of NaV1.1, to induce G1/S transition, albeit under an unknown mechanism.

Some minor points, for clarity:

1. Line 199: “whereas that of AKT was similar…” refers to mRNA.

2. Line 211: should refer to mock-treated cells, or similar (perhaps, it’s sufficient to indicate this in the figure legend, or simply in Methods), instead of “its MOCK”.

3. Line 340: Please use the correct verb in “The present study evaluated that SCN1A knockdown…” Perhaps, “We showed that…”

© 2024. Published by The Company of Biologists under the terms of the Creative Commons Attribution License (https://creativecommons.org/licenses/by/4.0/).

4. Line 463: Note that OptiPrepTM is a density gradient medium containing 60% (w/v) iodixanol, so it seems confusing the way it is written now.

Second revision

Author response to reviewers' comments

RE: Response to the comments from Reviewer 1

Comments from Reviewer 1

Reviewer 1 Advance Summary and Potential Significance to Field:

There are ample studies documenting the role of voltage-gated sodium channels (VGSCs) in tumor progression. This manuscript makes two further advances:
1) It defines a signal transduction pathway underlying VGSC control of cell cycling.

Response: We are very thankful to Reviewer 1 for the positive comment on our findings that the NaV1.1-mediated AKT/CDK2 pathway controls the cell cycle of mesenchymal stem cells.

2) It posits that a VGSC is acting in a vesicular compartment independent of its sodium conducting properties. However, this second crucial conclusion is not adequately supported by the data, and further experiments are warranted, as delineated in the suggestions to authors.

Response: Thank you for your concern about the action of VGSC. We respond to this point in the following part of "Reviewer 1 Comments for the Author".

Reviewer 1 Comments for the Author:
Significant revision in the form of confirmatory experiments is necessary to make this manuscript acceptable for publication:

A central thesis of this revised manuscript from Zakaria et al. is that Nav1.1's effects on mesenchymal stem cell proliferation are via a sodium current-independent pathway. Their supporting evidence for this claim is that plasma membrane voltage-clamped sodium currents could not be detected in the MSC cells. However, small sodium currents may be masked by outward leak and voltage-gated K currents, which are evident in the current traces in Figure 2. The experiments on SH-SY5Y cells, where sodium current is readily apparent and sensitive to TTX, does not resolve the problem with the MSC cells.

1) Recordings should be repeated: a) in presence of tetraethylammonium choride and 4-aminopurine to suppress voltage-gated K+ currents, and b) by applying P/N leak subtraction during recording (available in Clampex) to eliminate leak and capacitive currents.

2) Additionally, if the role of Nav1.1 on cycling is independent of sodium currents, the cycling effects should be resistant to TTX. Alternatively, if sodium currents contribute to cycling, the role of Nav1.1 should be TTX- sensitive. This is a simple experiment that the authors should undertake.

Response: We appreciate Reviewer 1 to the above two comments on our model that the sodium current-independent pathway controls the proliferation of mesenchymal stem cells. Your point, that voltage-gated potassium channel-mediated current-masked small voltage-gated sodium channel-mediated current may act on our model, makes us so excited. So, we repeated our tests following Reviewer 1's suggestion and showed the results in this re-revision.

Comments from Reviewer 2

© 2024. Published by The Company of Biologists under the terms of the Creative Commons Attribution License (https://creativecommons.org/licenses/by/4.0/).

Reviewer 2 Advance Summary and Potential Significance to Field:
In this revised version, the authors have addressed all the issues requested by the reviewers, involving the addition of some data, subsequently making several changes to the manuscript, which can be seen as considerably improved. The polished presentation now offered in both text and data is greatly appreciated.

Response: We are thankful to your effort to our revised manuscript.

Reviewer 2 Comments for the Author:
At this point, I can only point out some comments that - in my opinion - should be taken into account:
1. In general, the text should be checked for proper clarity - take a look, for instance, at the Abstract. As an example, when one talks about gene expression, referring to the process of converting genetic information into either RNA or protein, it sounds strange to say that "cells express" the SCN1A gene. Then, toward the end, "AKT inhibitor LY294002 did not... On the contrary, AKT activator SC79 did not…" If both "did not", one can't say "On the contrary", maybe "Likewise".

Therefore, authors must ensure proper usage of conjunctive adverbs - throughout the whole text - to avoid confusion and ensure smooth reading.

Response: Thank you for your point. We have corrected the parts in the re-revised version.

2. The non-canonical role of NaV1.1 described in this work provides added evidence for the involvement of voltage-gated ion channels in other functions. However, the subcellular location of these channels is an aspect that should not be overlooked since it will certainly influence the conclusions reached. The data added by the authors in this regard are only somewhat convincing, although I understand it is not easy to go further.
Virtually all NaV1.1 is intracellular in the cell model studied (just look at Fig. 1D and 1E) and, for the most part, that's why we don't see current.

Response: We appreciate your deep concern about our findings in this manuscript. We can agree with your opinion about the influence of the subcellular localization of NaV1.1 on the voltage-gated sodium current-independent function in mesenchymal stem cells. As you mentioned, if we can employ a model in which all NaV1.1 molecules localize intracellularly, not on the cell surface, it will be possible to improve the function of intracellular NaV1.1. In this re-revision, we added the results of tests suggested by Reviewer 1, which aim to examine whether voltage-gated potassium current (VGPC)- masked small VGSC participates in cell proliferation of mesenchymal stem cells. The results may support, at least partially, to clarify your concern.

3. Finally, undoubtedly, the knockdown effect achieved is not dramatic (Fig. 3A). Therefore, subsequent data can be expected to lack robustness. Compare, for instance, the nuclear localization of Cdk2 still observed in siRNA-treated cells (Fig. 6E) contrasting to a visible reduction shown earlier in the manuscript (Fig. 4C). Nonetheless, overall, it seems quite convincing that the Akt/Cdk2 pathway is downstream of NaV1.1, to induce G1/S transition, albeit under an unknown mechanism.

Response: We are thankful for your points on the visible matter. The reason why the visible discrepancy occurred between the two groups as raised by Reviewer 2 is as blow; To refuse optical bias in each experiment, we captured the fluorescent images under an optimal exposure time which was standardized to each reference in each experiment: for example, in Figure 4C, the reference was siCTRL-MSCs and in Figure 6E, the reference was MOCK-treated cells, which were almost equally to the reference in Figure 4C, siCTRL-MSCs. In the re-revised manuscript, we added the explanation of the image- capturing condition in the Methods parts.

Some minor points, for clarity:

1. Line 199: "whereas that of AKT was similar…" refers to mRNA.

© 2024. Published by The Company of Biologists under the terms of the Creative Commons Attribution License (https://creativecommons.org/licenses/by/4.0/).

2. Line 211: should refer to mock-treated cells, or similar (perhaps, it's sufficient to indicate this in the figure legend, or simply in Methods), instead of "its MOCK".

3. Line 340: Please use the correct verb in "The present study evaluated that SCN1A knockdown…" Perhaps, "We showed that…"

4. Line 463: Note that OptiPrepTM is a density gradient medium containing 60% (w/v) iodixanol, so it seems confusing the way it is written now.

Response: Thank you for the four minor points. We have corrected all the minor points raised by Reviewer 2 in the re-revised version.

Third decision letter

MS ID#: JOCES/2023/261732

MS TITLE: $Na_V1.1$ contributes to the cell cycle of human mesenchymal stem cells by regulating AKT and CDK2

AUTHORS: Mohammed Fouad Zakaria, Hiroki Kato, Soichiro Sonoda, Kenichi Kato, Norihisa Uehara, Yukari Kyumoto-Nakamura, Mohammed Majd Sharifa, Liting Yu, Lisha Dai, Haruyoshi Yamaza, Shunichi Kajioka, Fusanori Nishimura, and Takayoshi Yamaza

I am happy to tell you that your manuscript has been accepted for publication in Journal of Cell Science, pending standard publication integrity checks.

Reviewer 1

Advance summary and potential significance to field

This research describes a second intracellular function of a voltage-gated sodium channel as a participant in cell cycle progression.

Comments for the author

The authors have satisfactorily addressed my previous concerns, now convincingly showing that Nav1.1's effect on MSC cell cycling is independent of Nav1.1 sodium currents at the plasma membrane.

Reviewer 2

Advance summary and potential significance to field

The authors have addressed all issues requested by the reviewers.

This work explores, in non-excitable cells, a signaling pathway potentially activated by a NaV channel that leads to cell cycle progress. The key question is clear, and the data generated should lead to an important progress in the field.

Comments for the author

There is no doubt that the major location of NaV1.1 in MSCs is intracellular likely retained in the ER, which leads to no current measured. Taking into account its apparently reduced expression (compared to that in neural cells), whether actually the major role of NaV1.1, in these cells, is on

© 2024. Published by The Company of Biologists under the terms of the Creative Commons Attribution License (https://creativecommons.org/licenses/by/4.0/).

cell division or is a consequence of ER retention, is an open question. Similarly, it is unclear whether intracellular retention is regulated, or is rather caused by an unsuitable environment in the MSCs cytosol, along with a lack of the right NaV1.1 partners to ensure proper folding, including some necessary posttranslational modifications, such as glycosylation.

Other than these thought, I personally have nothing more to add.

© 2024. Published by The Company of Biologists under the terms of the Creative Commons Attribution License (https://creativecommons.org/licenses/by/4.0/).
